# Time‐specific and pleiotropic quantitative trait loci coordinately modulate stem growth in *Populus*

**DOI:** 10.1111/pbi.13002

**Published:** 2018-09-17

**Authors:** Qingzhang Du, Xiaohui Yang, Jianbo Xie, Mingyang Quan, Liang Xiao, Wenjie Lu, Jiaxing Tian, Chenrui Gong, Jinhui Chen, Bailian Li, Deqiang Zhang

**Affiliations:** ^1^ Beijing Advanced Innovation Center for Tree Breeding by Molecular Design Beijing Forestry University Beijing China; ^2^ National Engineering Laboratory for Tree Breeding College of Biological Sciences and Technology Beijing Forestry University Beijing China; ^3^ Key Laboratory of Genetics and Breeding in Forest Trees and Ornamental Plants Ministry of Education College of Biological Sciences and Technology Beijing Forestry University Beijing China; ^4^ Department of Forestry North Carolina State University Raleigh NC USA

**Keywords:** adaptive selection, epistasis, height‐diameter allometry, landscape genomics, *Populus*, QTL‐association mapping

## Abstract

In perennial woody plants, the coordinated increase of stem height and diameter during juvenile growth improves competitiveness (i.e. access to light); however, the factors underlying variation in stem growth remain unknown in trees. Here, we used linkage‐linkage disequilibrium (linkage‐LD) mapping to decipher the genetic architecture underlying three growth traits during juvenile stem growth. We used two *Populus* populations: a linkage mapping population comprising a full‐sib family of 1,200 progeny and an association mapping panel comprising 435 unrelated individuals from nearly the entire natural range of *Populus tomentosa*. We mapped 311 quantitative trait loci (QTL) for three growth traits at 12 timepoints to 42 regions in 17 linkage groups. Of these, 28 regions encompassing 233 QTL were annotated as 27 segmental homology regions (SHRs). Using SNPs identified by whole‐genome re‐sequencing of the 435‐member association mapping panel, we identified significant SNPs (*P *≤* *9.4 × 10^−7^) within 27 SHRs that affect stem growth at nine timepoints with diverse additive and dominance patterns, and these SNPs exhibited complex allelic epistasis over the juvenile growth period. Nineteen genes linked to potential causative alleles that have time‐specific or pleiotropic effects, and mostly overlapped with significant signatures of selection within SHRs between climatic regions represented by the association mapping panel. Five genes with potential time‐specific effects showed species‐specific temporal expression profiles during the juvenile stages of stem growth in five representative *Populus* species. Our observations revealed the importance of considering temporal genetic basis of complex traits, which will facilitate the molecular design of tree ideotypes.

## Introduction

To adapt to environmental conditions, plants undergo dynamic developmental changes that are induced by complex external and internal stimuli (Chitwood *et al*., 2014; Yang *et al*., 2013). For example, many species exhibit rapid increases in stem height and root length during the juvenile stage to reach light and water, thus helping the juvenile plants quickly establish themselves in a competitive environment (Grattapaglia *et al*., 2009). Consistent with the importance of growth for adaptation, plant growth is orchestrated in a precise, quantitative manner, involving the interplay of myriad tightly regulated genes, some that play essential roles at a specific growth phase, and others that play a more general role during development (Bac‐Molenaar *et al*., 2015; Lauter *et al*., 2005).

Growth from cell division in plants occurs in meristems and studies of herbaceous model species have improved our understanding of the temporal changes controlling the shoot and root apical meristems throughout the annual life cycle (Chuck *et al*., 2007; Geng *et al*., 2013; Lauter *et al*., 2005; Schmid *et al*., 2005). Genes underlying dynamic growth patterns form a robust regulatory network that enables the herbaceous plant to complete its life cycle under changing conditions (Bac‐Molenaar *et al*., 2015).

Although growth in annual plants has been extensively studied, we lack an understanding of the regulatory networks underlying allometry of primary and secondary meristems in perennial woody species. Perennial woody species, especially *Populus*, are excellent experimental systems for understanding stem growth dynamics. In particular, the allocation of the growth of stem height and diameter during the juvenile phase of trees reflects their capacity to respond to changing conditions and is vital to ensure survival in natural and planted forests (Hulshof *et al*., 2015). Stem growth in trees is controlled by cell division and expansion in the apical and cambial meristems (Grattapaglia *et al*., 2009). The apical meristems are mainly responsible for overall plant height and the secondary meristems contribute to radial wood accumulation, but they share overlapping regulatory systems (Mizrachi *et al*., 2017). In this study, we explore the genetic factors underlying stem height and diameter at multiple timepoints during juvenile growth of *Populus* at the population level.

The identification of genetic factors affecting the development of complex traits in perennial species requires innovative genetic methods. Recently, joint linkage‐linkage disequilibrium (linkage‐LD) mapping approaches, including parallel mapping (independent linkage and LD analysis) and integrated mapping (datasets analysed in combination), have been developed (Motte *et al*., 2014; Sterken *et al*., 2012). These methods can overcome the inherent limitations of quantitative trait loci (QTL) and association mapping approaches, such as low mapping resolution in QTL mapping and the presence of rare alleles and population structure in LD mapping, and thus have enabled a closer examination of the allelic variants responsible for a phenotype in several model plant species (Du *et al*., 2016; Motte *et al*., 2014; Sterken *et al*., 2012). However, the application of linkage‐LD mapping approach has not yet been attempted for dissecting the dynamic processes of complex quantitative traits in perennial plants, such as poplar species.

Herein, we used the linkage‐LD method to decipher the dynamic genetic interactions (under additive, dominant and epistatic effects) affecting three stem growth traits at dynamic timepoints throughout the juvenile growth phase, in a *Populus* full‐sib family population and an association mapping panel of 435 unrelated individuals. We also uncovered the potential signatures of selection within segmental homology regions (SHRs) of known QTL among three climatic regions, and finally dissected the biological function of potential time‐specific genes and the favourable alleles underlying stem growth.

## Results

### Phenotypic variation in stem growth at multiple timepoints in the *Populus* linkage population and association mapping panel

To examine the genetic basis for growth traits, we first measured the phenotypic variation in our populations. For the linkage population, we measured tree height (H), basal diameter (BD), and stem volume (V) during growth of 1,200 juvenile *Populus* progeny at 12 timepoints over the course of the first growing season (180 days). This revealed remarkable phenotypic variation in stem growth during the juvenile growth phase (Table S1 and Figure S1a–c). The growth patterns of the three traits among progeny at this stage were more similar to the growth of ‘LM50’, the male parent used to establish the population (Figure S1a‐c). The two parents of the population displayed a dramatic difference in the height‐to‐diameter ratio (H/D) over the juvenile growth phases, and the average trunk diameter of the progeny was more slender than those of the two parents (Figure S2a). Considerable variation in the H/D trajectory among progeny (Figure S2a) reveals possible involvement of multiple QTL that controls juvenile growth.

We next examined the phenotypic variation in the association panel of 435 unrelated individuals of *P. tomentosa*, including three sub‐populations from different climatic zones, the southern (S), north‐western (NW) and north‐eastern (NE) climatic regions (Du *et al*., 2012). We measured H, BD and V at five timepoints over the course of the first growing season (180 days) and thereafter for four annual measurements. In the association mapping panel, more extensive phenotypic variation was observed at each timepoint than that observed in the linkage population (Table S1 and Figure S1d‐f). The H/D decreased rapidly over the first growing season and then gradually levelled off for the total population and three sub‐populations (Figure S2b).

### Dynamic QTL associated with stem growth in the linkage population

**Figure 1:**
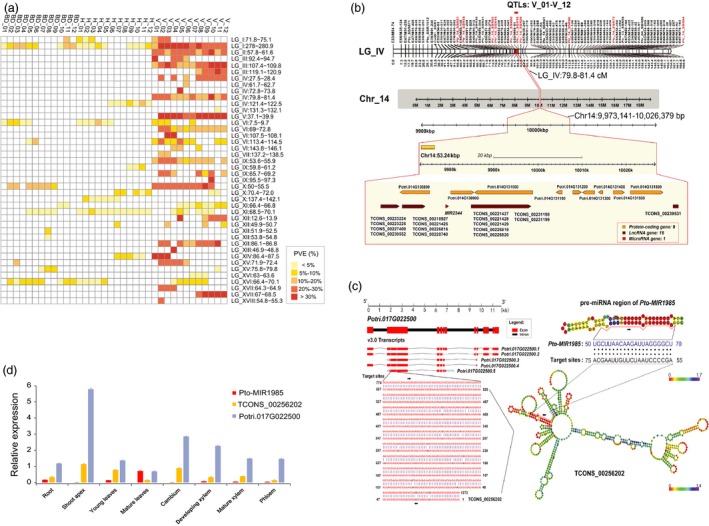
Identification and annotation of quantitative trait loci (QTL) for three stem growth traits over 12 growth timepoints in *Populus*. (a) Significant QTL (the logarithm of odds, LOD score ≥ 3.0) were detected for the basal diameter (BD), tree height (H) and stem volume (V) at 12 growth timepoints during the first growing season, which can be categorised into 42 discrete pleiotropic regions across 17 linkage groups (LGs). Each QTL explained 2.8%–38.1% of the phenotypic variance. (b) A major discrete pleiotropic region (79.8–81.4 cM) on LG_IV encompassing significant QTL hotspots for V over all 12 timepoints strongly corresponds with the segmental homology region 18 (SHR18, 9.97–10.03 Mb) on Chromosome 14 (Chr_14), defined using comparative linkage mapping and the *Populus* reference genome. (c) *Potri.017G022500* is predicted as the target of lncRNA *TCONS_00256202*, and *TCONS_00256202* is the potential target of miRNAs *Pto‐MIR1985*. (d) A negative tissue‐specific expression profile was observed between *Pto‐MIR1985* and its target lncRNA *TCONS_00256202*, whereas a positive expression pattern was observed between *TCONS_00256202* and its potential target *Potri.017G022500*.

Next, we used the phenotypic measurements for the linkage population to uncover the genetic loci that affect stem growth in this population, using a QTL mapping approach. We used 929 amplified fragment length polymorphism (AFLPs), 309 simple sequence repeat (SSRs) and 32 insertion‐deletions (InDels) on the linkage map (Du *et al*., 2016) to conduct QTL analysis. This detected 311 significant QTL (logarithm of odds, LOD ≥ 3.0, Table S2) for the three stem growth traits across all 12 timepoints during the first growing season, and each QTL gave phenotypic variance explained (PVE) values of 2.8%–38.1%. A region that contained multiple co‐localised QTL within the same confidence interval was defined as a discrete overlapping region. All 311 QTL could be categorised into 42 discrete overlapping regions across 17 linkage groups (LGs), and each overlapping region contained 1–30 QTL (Figure 1a and Table S3).

Examination of the dynamic QTL profile over time (Figure 1a) showed that some overlapping regions associated with all three traits were identified in a continuous set of timepoints, some were identified at multiple discontinuous timepoints, and some were specific for either a trait or a timepoint. This likely reflects the specific or pleiotropic roles of these QTL underlying stem growth at different stages. We focused on the discrete overlapping regions that affected a particular trait at continuous timepoints (Figure 1a). The individual PVE of some dynamic QTL remained at similar level, but for others the PVE changed substantially across the continuous timepoints (Figure 1a). Ten significant QTL hotspots were determined with the permutation thresholds (QTL number per cM ≥ 7.0, α < 0.05, Table S3).

### Annotation of candidate genes within genomic segmental homology regions (SHRs) of the QTL

To fine dissect the discrete regions that contain QTL for stem growth, we next annotated the genes within those discrete regions by using the reference genome of *P*. *trichocarpa*. Comparison of the positions of anchoring gene‐derived markers on the linkage map (Du *et al*., 2016) and the annotated *P*. *trichocarpa* reference genome v3.0, allowed us to identify the putative genomic SHRs of the dynamic QTL. We calculated the relative genomic size of each SHR that corresponded to the confidence interval of each QTL (Table S3), by a comparison of the map distances (cM) and genomic sequence length (Mb) between at least three anchoring gene‐derived markers within or near the QTL (Du *et al*., 2016). Twenty‐seven putative SHRs were identified on 14 chromosomes of the *P. trichocarpa* reference genome v3.0, representing the 28 corresponding discrete regions distributed among the 14 LGs, and encompassing 75.1% (233) dynamic QTL (Table S3).

We next examined 640 unique protein‐coding genes (PCGs), 430 genes encoding long noncoding RNA (lncRNA) from 121 overlapping genomic loci and 28 genes for precursors of microRNA (pre‐miRNA), covering all genomic sequences of the 27 putative SHRs (Figure S3a and Data S1–S3). For instance, a discrete overlapping region (79.8–81.4 cM) on LG_IV, as a significant QTL hotspot for V over 12 continuous timepoints (12 QTL, average PVE = 23.1%), was the syntenic genomic region of SHR18 (9.97–10.03 Mb) on Chr_14 (Figure 1b).

Gene ontology (GO) analysis of the 640 PCGs revealed that the significantly enriched categories (FDR ≤ 0.05) mainly include ‘transferase activity, transferring glycosyl groups’ and ‘transcription regulator activity’ (Data S4). The actions of glycosyltransferases are critical for polysaccharide synthesis of plant cell walls (Keegstra and Raikhel, 2001). In particular, 38 transcription factor (TF) genes annotated within 15 SHRs, mostly enriched in stress‐responsive *WRKY*,* AP2/ERF*,* bZIP* and *MYB* TF families (Data S1), suggesting these TFs enable plants to respond rapidly to stress and ensure survival during early growth.

Our predictions further determined 1,154 lncRNA–target gene pairs within all 27 SHRs, and 68.4% (789) were considered as potential *cis*‐target pairs (Figure S3b and Data S5). Also, 48 PCGs and 129 lncRNA genes were predicted to be potential target genes of 26 miRNAs (Figure S3b and Data S6). For example, *Potri.017G022500* within SHR24, encoding a zinc finger BED domain‐containing protein (ZBED), was predicted as the *cis*‐target of lncRNA *TCONS_00256202* and *TCONS_00256202* is the target of *Pto‐MIR1985* (Figure 1c), which was consistently correlated with their tissue expression patterns examined in 1‐year‐old *P. tomentosa* (Figure 1d).

### SNP annotation and LD estimation within the SHRs in the association mapping panel

To validate these dynamic QTL across populations, we next identified SNPs within the SHRs in the association mapping panel. Analysis of the re‐sequencing data of the association mapping panel identified 52 949 high‐quality SNPs from the 27 SHRs (Table S4 and Data S7). The SNPs were not evenly distributed within each SHR, with an average SNP density per SHR ranging from 45 to 219 bp^−1^ (Table S4). 64.9% SNPs were annotated into the intergenic regions, including 1,699 intergenic SNPs also located within the noncoding RNA genes (Table S4 and Data S7). The nucleotide diversity (π) in the noncoding sequences of PCGs was twofold higher than in the coding sequences (Table S4), where nonsynonymous diversity was one‐third (*d*_N_/*d*_S_ = 0.34) of the synonymous diversity.

Using the entire association mapping panel, we pooled estimates of the squared allele‐frequency correlations (*r* ^2^) for all pairwise SNP combinations within each SHR. This found 684 high‐LD blocks (*r* ^2^ ≥ 0.75, *P *≤* *1.0E‐03, Figure S4), with a block size of 12–2,154 bp. The high‐LD blocks were not evenly distributed within all 27 SHRs and mostly occurred within PCGs or noncoding RNA genes. Some haplotypes spanned lncRNAs and their predicted *cis*‐target PCGs, and had higher *r* ^2^ and lower π than the average values of all haplotypes across the SHRs. The patterns of LD decay varied extensively across the SHRs, with the *r* ^2^ values declining to 0.2 at distances of 0.3–60 kb (Figure S5). No significant correlation was identified between the extent of LD and the genomic size of the SHR.

### Identification of potential signatures of selection within the SHRs in the association mapping panel

**Figure 2:**
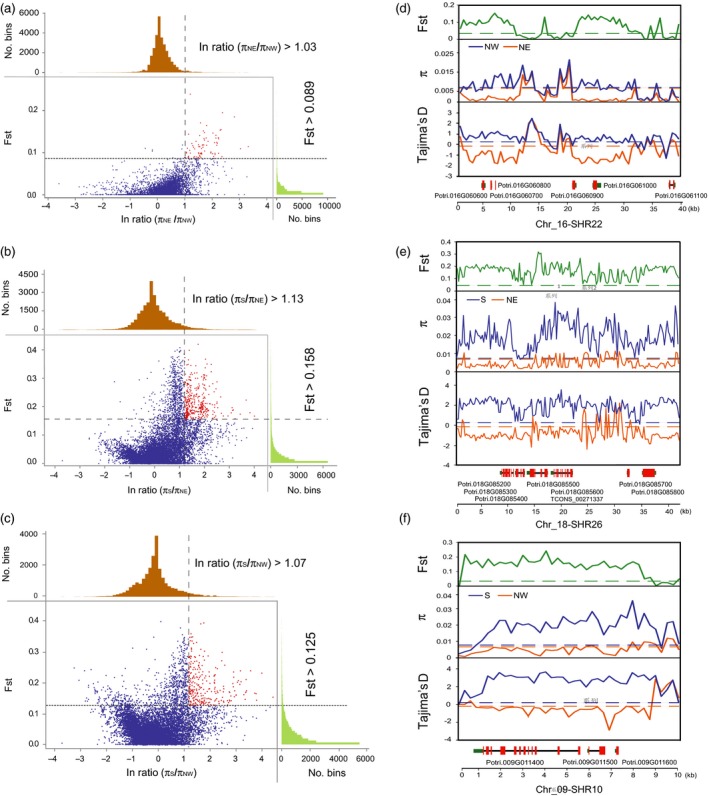
Adaptive selection signals within 27 segmental homology regions (SHRs) among the three natural climatic regions of *Populus tomentosa*. The empirical distribution of log‐ratios (π_region_1_/π_region_2_) and Fst values within all 27 candidate SHRs with 250‐bp steps of each pairwise climatic regions. Red dots represent windows fulfilling the significant selection between each pairwise regions, (a) north‐western (NW) relative to north‐eastern (NE), (b) NE relative to southern (S), and (c) NW relative to the S climatic regions of *Populus tomentosa*. The latter is the reference population and the former is the objective population. Example of genes with adaptive selection signals within SHR22 (d), SHR26 (e) and SHR10 (f) between each pairwise comparison. Fst, π and Tajima's D values are plotted using a 250‐bp sliding window. Objective population (orange), reference population (blue). Horizontal dashed lines represent corresponding mean values among all SHRs. Genes are shown at the bottom (red rectangle, coding sequences; black line, introns; green rectangle, 5′ and 3′ untranslated regions).

To determine whether the SHRs show genomic signatures of selection in the association mapping panel, we looked for evidence of selection in the sub‐populations from different climatic regions, which presumably have adapted to their differing environments. We computationally detected potential signals of selection in 250‐bp non‐overlapping sliding windows within all 27 SHRs between the three climatic regions of *P. tomentosa* (Table S5 and Data S8). In total, 84 (NW vs. NE), 629 (NW vs. S) and 534 (NE vs. S) significant sliding windows (Figure 2a–c and Table S5) were detected based on the top 1% of the empirical distribution of log‐ratios (π_region_1_/π_region_2_) and the population‐differentiation statistic (Fst) values of each pairwise comparison between climatic regions (Table S5). These sliding windows were confirmed by their significantly lower Tajima's *D* values in the objective population compared to the reference population (Figure 2d–f and Data S8). We further identified 40 (NW vs. NE), 133 (NW vs. S) and 89 (NE vs. S) genomic signatures of selection (104 unique regions in total, ranging from 0.25 to 41.2 kb with an average size of 4.3 kb) within the 27 SHRs (Table S5 and Data S8).

These 104 potential selective regions harboured 132 PCGs, 128 lncRNA genes from 35 overlapping genomic amplicons, and a pre‐miRNA gene (Table S5 and Data S9). Functional categories for these PCGs suggest that 16 TF genes were overrepresented in all pairwise comparisons (NW vs. S, NE vs. S or NW vs. NE; Table S6). Moreover, 29 PCGs with functional annotations related to cell wall biosynthesis, such as sugar transporter, UDP‐glucosyl transferase and cellulose synthase, were repeatedly overrepresented in S vs. NW and S vs. NE groups (Table S6).

We also found some genes related to response to stimuli and metabolic processes (Table S6). For example, five PCGs (*Potri.016G060600* to *Potri.016G061100*) located on SHR22, encoding zinc finger proteins and disease resistance‐responsive family proteins, which showed very strong selection signals in the NE vs. NW comparison (Figure 2d). Another seven PCGs and one lncRNA gene (*TCONS_00271337*), located on SHR26, showed significant signals of selection in the NE vs. S comparison (Figure 2f). Indeed, SHR‐derived PCGs related to cell wall, transcriptional regulation and stress response were over‐represented in the different comparisons. The selected signatures might be involved in the regulation of growth for adaptation to specific climatic regions.

We identified 25 PCGs and 32 lncRNA genes from seven overlapping genomic amplicons that showed strong selective signals for at least two sets of climatic regions (Table S6 and Data S9). For example, three PCGs within SHR10 were identified as subjected to selection for the NW vs. S comparison (Figure 2f), and the NE vs. S comparison (Data S9). Furthermore, we screened 109 representative SNPs within the selective genes that were highly differentiated in allele frequency among the three climatic regions (Data S10). Among these, 45 (~41.3%) SNPs were nearly fixed (with a minor allele frequency (MAF) < 0.05 in one or two climatic regions) and 14 (~12.8%) SNPs were completely fixed in specific regions (no allele observed in one climatic regions, MAF = 0) (Data S10).

### The genetic effects of allelic variants within SHRs underlying stem growth revealed by association mapping

We next aimed to dissect the genetic effects (additive, dominance and epistasis) of allelic variants within SHRs of QTL for the same three growth traits over a longer period of growth (nine timepoints, a 5‐year period) in the association mapping panel.

#### Additive and dominance of natural variants for stem growth over nine timepoints

We performed a mixed linear model (MLM) association mapping approach to examine the additive/dominant effect between 52 949 high‐quality SNPs and 27 phenotypic sets (three traits, nine timepoints). As shown in the quantile‐quantile and Manhattan plots for the 27 phenotypic data (Figures S6–S8), a range of 15–766 unique SNPs for each trait at each timepoint (Table S7 and Data S11) were identified at a significant threshold of *P *=* *1.9 × 10^−5^ (1/n, suggestive *P*‐value, Figure S9). We found 53% of the associated SNPs were annotated within PCGs or noncoding RNA genes to construct the complex lncRNA‐miRNA–PCG networks for each trait over nine timepoints (Figure S10), where 25 (6.3%) PCGs and 34 (4.1%) lncRNAs were identified as association hotspots (repeatedly associated with at least 12 phenotypic sets, α < 0.05) (Figure S11).

Even when considering a more stringent Bonferroni‐adjusted threshold of *P *=* *9.4 × 10^−7^ (0.05/n, significant *P*‐value), we detected 652 (BD), 179 (H) and 1,758 (V) associations among the nine timepoints, representing 411 unique SNPs for BD, 156 for H and 985 for V (Data S11). In these, only 12% of associations were detected for the first growth season (five timepoints, T1‐01 to T1‐05). Approximately, 29.2% of SNPs were annotated in the 311 PCGs and/or 87 overlapping lncRNA genes, where 11 PCGs and nine lncRNAs, encoding proteins such as WRKY03, Glutamate receptor 2.7 (GLR2.7), and a protein with a domain of unknown function 594 (DUF594), were the possible association hotspots (repeatedly associated with at least eight phenotypic sets at significant *P*‐value, α < 0.05) (Figure S11).

In summary, we found that 60.9% of the associations were exclusively related to dominant effects (the mean phenotypic value of the heterozygous genotypic class is significant difference with the value across both homozygous classes, based on least square means), and 12.0% were related to both additive and dominant effects (*P *<* *0.001, Data S11) over the growth processes. For all of the pleiotropic SNPs over the entire nine timepoints, we summarised four patterns of genetic effects exerted by a single SNP: (1) additive effect for one trait monotonically, (2) dominant effect for one trait monotonically, (3) additive and dominant effects for one trait jointly, and (4) either additive or dominant effect for all traits. We further found four common patterns of the PVE by a locus over the entire nine timepoints: (1) contributions increase consistently, (2) contributions decrease consistently, (3) contributions level off consistently over time and (4) contributions increase rapidly and then gradually level off. The diverse patterns of genetic effects and PVE for SNPs Chr04_11343447 and Chr16_5505403 were exemplified in Figure S11.

#### Pairwise epistasis of natural variants for stem growth over nine timepoints

To test for possible epistatic interactions during stem growth, we calculated the pairwise epistatic interactions between all common SNPs within these SHRs against BD, H and V traits across the nine timepoints respectively (*P *<* *0.001, Table S8 and Data S12). Diverse patterns of pairwise interactions were predicted for each trait over the nine timepoints, where no significant epistatic interaction was detected for V at the fourth timepoint in the first growth season (V1‐04). These overall epistatic SNP pairs were located in 2,709 intergenic variants, 1,587 PCG loci and 160 unique variants from noncoding RNA genes among 26 SHRs (except SHR05). We then identified 10 791 epistatic SNP pairs (16.2%) in which both loci derived from the annotated PCGs and/or the miRNA and lncRNA genes, representing a wide range of 10 (H1‐03, stem height at the third timepoint) to 3,442 (V1‐01, the first timepoint measured for V) overlapping locus–locus interactions (Table S8 and Figures S13–S15).

**Figure 3:**
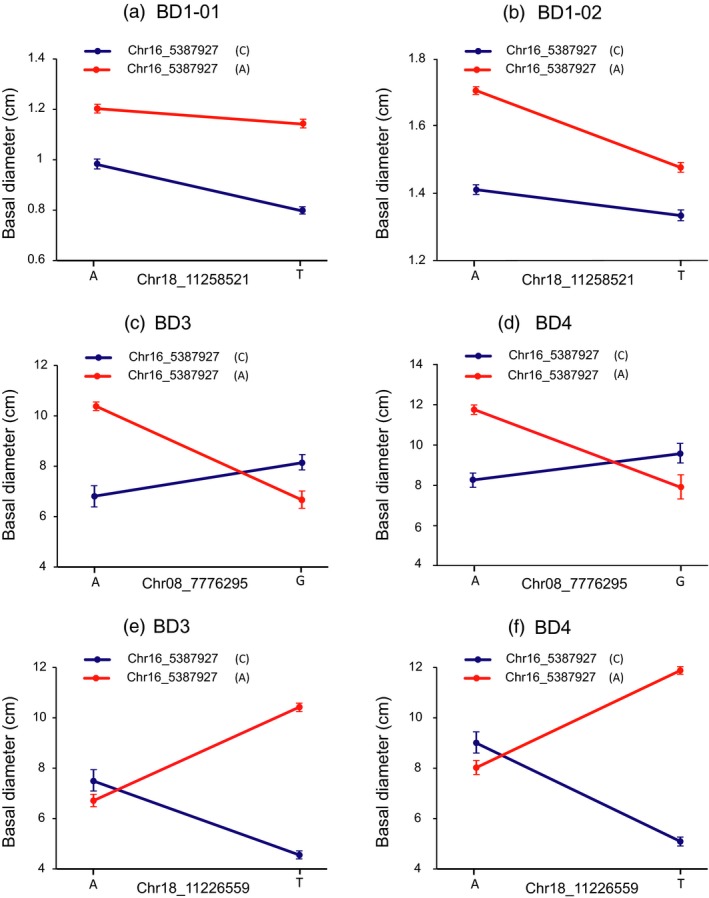
Allelic interactions between significant pairwise SNPs showed considerable, unexpected epistasis for BD over the growth timepoints. (a, b) Different types of interactions identified between the two major SNP loci (Chr16_5387927 and Chr18_11258521) underlying BD1‐1 and BD1‐2. The T allele (Chr18_11258521) depends on the C allele (Chr16_5387927) for decreasing BD1‐1, whereas the T allele (Chr18_11258521) depends on the A allele (Chr16_5387927) for decreasing BD1‐2. (c,d) A genotypic combination of A and A at SNP Chr16_5387927 (A or C) and SNP Chr08_7776295 (A or G) loci confers the highest variation in BD3 and BD4, but the other genotypic combinations between both loci accumulate either much lower or median variations at the BD3 and BD4 timepoints. (e,f) The pairwise interactions between SNP Chr16_5387927 (A or C) and SNP Chr18_11226559 (A or T) control BD3 and BD4 with different genotypic combinations at the two loci.

We focused on the pairwise epistatic interactions between these SNPs with significant additive and/or dominance within SHRs. A total of 86 significant interactions (*P *<* *0.001) were detected for V and BD at multiple timepoints (Data S13). As exemplified by a major associated SNP (Chr16_5387927) that significantly interacted with three other significant SNPs (Chr08_7776295, Chr18_11258521, and Chr18_11226559) for BD at different timepoints (Figure 3), we found that different allelic combinations between these SNPs showed unexpected and considerable non‐additive effects over time. The significant interaction between the two major SNPs (Chr16_5387927 and Chr18_11258521) showed a different scenario for BD at the first timepoint (BD1‐01, Figure 3a) and BD at the second timepoint (BD1‐02, Figure 3b). Also, we found a genotypic combination of A (Chr16_5387927) and A (Chr08_7776295) at the pairwise interaction contained the highest BD3 (3‐year‐old basal diameter, Figure 3c) and BD4 (4‐year‐old basal diameter, Figure 3d). Similarly, SNP Chr16_5387927 (A/C) and SNP Chr18_11226559 (A/T) had a pairwise interaction with BD3 and BD4, where the highest BD3 and BD4 were observed in genotypes with a combination of A and T, and the lowest BD3 and BD4 were found in genotypes with a combination of C and T alleles. Therefore, it is possible that these two loci act in parallel to determine BD (Figure 3e,f).

### Identification of possible causative SNPs that underlie the stem growth phases in *Populus*

Our association mapping studies helped us to further uncover the most strongly associated loci within QTL underlying stem growth, including the lead SNPs (the SNP with the most significant *P*‐value) or high‐LD clusters of multiple top‐ranked associated SNPs underlie the stem growth phases in *Populus*. In total, 22 lead SNPs were detected within nine SHRs that may represent the possible causative signals for each trait at the nine timepoints (Table 1). Of these, 16 lead SNPs (genic variants) were located directly within 15 candidate PCGs (that overlap with four lncRNA genomic amplicons, Table 1) that have potential roles in cell metabolism, growth and differentiation. For example, SNP Chr08_7687813, in the 3′UTR of *Potri.008G119100* (encoding a protein kinase superfamily protein, PKSP), was the peak associated variant for BD1‐03 (1‐year‐old basal diameter at the third timepoint, *P *=* *7.11 × 10^−8^) and BD1‐04 (1‐year‐old basal diameter at the fourth timepoint, *P *=* *2.66 × 10^−7^) (Figure S16b).

**Table 1:** Major SNPs linked to candidate genes significantly associated with three growth traits over nine timepoints in *Populus* association mapping panel

Trait^a^	SHR	*P*‐value^b^	Possible causative SNP^c^	Alleles	Protein‐coding genes^d^	Position^e^	Selective loci/genes^f^	Description^g^	Noncoding RNA gene^h^
BD1‐01	21	1.02E‐07	Chr16_677430	A:G	Potri.016G012300	3′down	Y	*LRR‐RLK*	
BD1‐02	21	4.24E‐07	Chr16_677430	A:G	Potri.016G012300	3′down	Y	*LRR‐RLK*	
BD1‐03	8	7.11E‐08	Chr08_7687813	G:T	Potri.008G119100	3′UTR	Y	*PKSP*	TCONS_00140766‐TCONS_00153166
BD1‐04	8	2.66E‐07	Chr08_7687813	G:T	Potri.008G119100	3′UTR	Y	*PKSP*	TCONS_00140766‐TCONS_00153166
BD1‐05	3	5.52E‐10	Chr04_4039502	C:T	Potri.004G051900	Intron3	Y	*GDSL‐like Lipase/Acylhydrolase*	
BD2	8	1.26E‐11	Chr08_7290478	G:A	Potri.008G114200	Intron8	Y	*PPC4*	TCONS_00141932
BD3	21	1.48E‐13	Chr16_680030	T:C	Potri.016G012400	Exon1	Y	*DUF594*	
BD4	4	1.28E‐13	Chr04_11343447	T:C	Potri.004G120800	Exon4	N	*WRKY3*	
BD5	8	4.07E‐14	Chr08_7880811	G:A	Potri.008G121200	Intron3	Y	*JAG*	
H1‐01	6	6.96E‐07	Chr06_26424501	G:A	Potri.006G261000	Downstream	Y	*MYB‐like HTH*	
H1‐02	4	8.43E‐08	Chr04_10812886	G:T	Potri.004G116700	3′down	N	*PCI*	
H1‐03	22	5.73E‐08	Chr16_4232061	G:A	Potri.016G060600	Promoter	Y	*ZFP8*	
H1‐04	4	3.15E‐08	Chr04_11364096	G:A	Potri.004G120900	Downstream	N	*DUF3598*	
H1‐05	8	1.75E‐07	Chr08_7871772	A:T	Potri.008G121100	Promoter	Y	*RPL15*	
H2	21	1.40E‐08	Chr16_680796	A:T	Potri.016G012400	Exon1	Y	*DUF594*	
H3	21	7.05E‐09	Chr16_680796	A:T	Potri.016G012400	Exon1	Y	*DUF594*	
H4	8	7.14E‐09	Chr08_7239399	C:A	Potri.008G113300	3′down	N	*ZIP7*	
H5	8	3.25E‐11	Chr08_7834452	T:C	Potri.008G120600	3′down	Y	*LOB42*	
V1‐01	3	1.24E‐17	Chr04_4102141	C:T	Potri.004G052500	Intron4	Y	*GLR2.7*	TCONS_00078983‐TCONS_00082961
V1‐02	7	1.36E‐09	Chr08_5973087	C:T	Potri.008G095300	3′down	N	*PDCB3*	
V1‐03	6	2.71E‐07	Chr06_26424832	C:T	Potri.006G261000	Downstream	Y	*MYB‐like HTH*	
V1‐04	4	1.19E‐08	Chr04_11364074	T:A	Potri.004G120900	Downstream	N	*DUF3598*	
V1‐05	2	8.48E‐08	Chr03_14634651	C:A	Potri.003G125200	3′down	N	*DUF567*	
V2	21	1.03E‐10	Chr16_736028	G:A	Potri.016G013200	Intron2	N	Unknown	TCONS_00138008‐TCONS_00146931
V3	21	2.01E‐15	Chr16_736028	G:A	Potri.016G013200	Intron2	N	Unknown	TCONS_00138008‐TCONS_00146931
V4	23	1.79E‐15	Chr16_5505403	G:T	Potri.016G073400	Upstream	Y	Unknown	
V5	23	1.47E‐16	Chr16_5505403	G:T	Potri.016G073400	Upstream	Y	Unknown	

Segmental homology regions (SHRs), Basal diameter (BD), Tree height (H), Stem volume (V), untranslated region (UTR). ^a^Each growth trait over nine timepoints in *Populus*. ^b^The most significant *P*‐value of the SNP (lead SNP^c^) for each trait only. ^d^A plausible biological candidate gene in the locus or the nearest annotated gene to the lead SNP. ^e^Position for the lead SNP according to the annotation genes in the *Populus* genome V.3.0; ^f^The candidate gene located in the significant selection intervals among pairwise climatic regions of *Populus* (Y), not located in any significant selection region (N); ^g^Each candidate gene was annotated according to Popgenie (http://popgenie.org/), The full name of each gene is shown in the Date S1. The overlapped long noncoding RNA genes^h^ (lncRNA, TCONS*_*code) in the locus or the nearest noncoding RNA genes, more information is listed in Data S2.

In addition, we screened six intergenic SNPs that located close to (approximately 0.3–7 kb) the four PCGs, which were the peak associated variants for growth traits at multiple timepoints (Table 1). For example, a noncoding SNP (Chr08_7239399), located 452 bp downstream of *Potri.008G113300* (encoding a zinc transporter 7 precursor protein, ZIP7, Figure S16g), was the peak signal for H4 (4‐year‐old stem height, *P *=* *7.14 × 10^−9^, Figure S16b). No significant epistatic interactions (*P *<* *0.001) in pairwise combinations were observed for the lead SNPs and top‐ranked associated SNPs.

We next identified 12 representative PCGs with three lncRNA genes that overlapped with several significant signals of selection between the climatic regions of *P. tomentosa* (Table 1). The allelic distributions of 12 lead SNP sites exhibited frequency differences between the three climatic regions (Figure S17). For example, *LATERAL ORGAN BOUNDARIES 42* (*LOB42*), which had the most significant SNP for H5 (5‐year‐old stem height*, P *=* *3.25 × 10^−11^, Figure S16b–d), showed obvious regional differentiation, as the mutant allele (C) increased in frequency from the S to the NW or NE regions (Table 1 and Figure S17). The regional differentiation was potentially similar to the patterns of *GLR2.7*,* LRR‐RLK* (encoding a leucine‐rich repeat transmembrane protein kinase), *JAG* (encoding a C2H2 and C2HC zinc fingers superfamily protein), *PPC4* (encoding phosphoenolpyruvate carboxylase 4 protein) and an unknown PCG (*Potri.016G073400*), where the major SNPs with presumably mutant alleles were more likely to be found at high frequency in the high latitude northern China (Figure S17).

### Allelic interpretation of the biological function of potential genes and SHRs

Combining the above association analysis, regional selection, QTL mapping and re‐sequencing of these candidate genes, we were able to potentially update functional annotation of the most likely causal genes, including many (approximately one‐third) novel genes. We next chose several SHRs and candidate genes involved in specific growth phases to investigate the potential functional polymorphisms capable of affecting the stem growth processes.

In SHR04, SNP Chr04_11343447 (T/C), a nonsynonymous variant within exon 4 of a key TF gene, *WRKY3* (*Potri.004G120800*), which produces a Lys > Arg substitution, is the lead SNP for BD4 (4‐year‐old basal diameter, *P *=* *1.28 × 10^−13^, Table 1), and is also the top‐ranked significant variant for BD5 (5‐year‐old basal diameter, *P *=* *1.96 × 10^−12^, Data S11). Additional analysis showed that this nonsynonymous variant is the second most strongly associated variant for V4 (4‐year‐old stem volume, *P *=* *3.78 × 10^−14^) and V5 (5‐year‐old stem volume, *P *=* *3.01 × 10^−16^) with similar genetic effect (Data S11). The wild‐type allele (T) confers slow stem growth and the minor allele (C) confers fast stem growth at the fourth and fifth year growth stage in natural populations. The expression level of *WRKY3* was higher in the vascular cambium than in the shoot apical meristem (SAM) in the 4‐ to 5‐year‐old *P. tomentosa* clones (Data S14), suggesting that *WRKY3* is temporally involved in stem growth, mostly in radial secondary growth rather than in the apical meristems of *P. tomentosa*. Similarly, we detected the lead SNP Chr16_5505403 (G/T) in SHR23 that simultaneously associated with the V4 (*P *=* *1.79 × 10^−15^) and V5 (*P *=* *1.47 × 10^−16^) (Table 1 and Figure S18). The SNP variant is located 7.1 kb downstream of the stop codon of a novel gene (*Potri.016G073400*), with significant regional selective signals (Figure S17). This pleiotropic SNP also had the top‐ranked associated signals for BD4 (*P *=* *2.11 × 10^−13^), BD5 (*P *=* *1.02 × 10^−13^), H4 (*P *=* *4.10 × 10^−8^), H5 (*P *= 5.46 × 10^−10^) and the expression of *Potri.016G073400* (*P *=* *2.78 × 10^−6^) (Figure S18 and Data S11), suggesting that this locus may be involved in the transcriptional regulation of *Potri.016G073400* to control the natural variation during the fourth and fifth years of growth of poplar.

**Figure 4:**
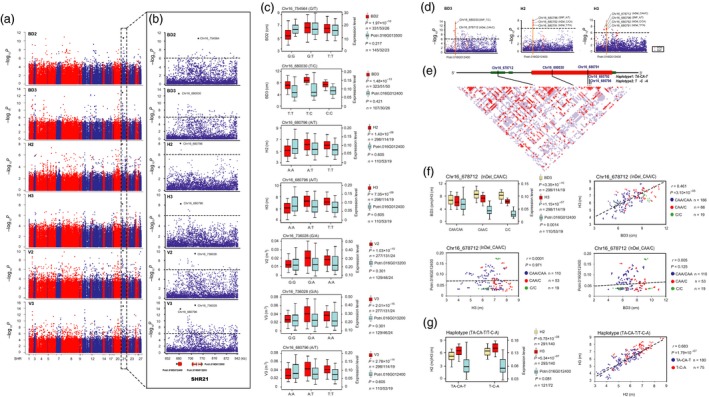
Several major SNPs linked to novel genes in SHR21 may underlie stem growth in the second and third years of growth (a) Manhattan plots displaying the association results between all SNPs of 27 SHRs and growth traits at the second and third years of growth. The *x*‐axis shows the SHR positions and the *y*‐axis shows the significance expressed as −log_10_ *P*‐value. (b) The lead SNP (the SNP with the lowest *P*‐value) for each trait, whose position is indicated in the SHR21 locus by a larger black dot. The dashed horizontal line depicts the Bonferroni‐adjusted significance threshold (9.4 × 10^−7^). Three linked genes are shown at the bottom (red rectangle, coding sequences; black line, introns; green rectangle, 5′ and 3′ untranslated regions). (c) Box plot for each growth trait (red) and expression of linked gene (sky blue) is plotted as an effect of genotypes at the lead SNP. The horizontal line represents the mean and the vertical lines mark the range from the 5th and 95th percentile of the total data. (d) In each plot, the most significantly associated SNPs (larger blue dots) and InDels (larger red dots) across the *Potri.016G012400* locus for BD3, H2 or H3 are indicated by a vertical orange solid line. (e) A linkage disequilibrium (LD) representation of the pair‐wise *r* ^2^ value among all polymorphic sites (SNPs and InDels) across the *Potri.016G012400* locus, where the deeper red color of each box corresponds to the higher *r* ^2^ value. (f) Genotypic effect of the significant InDel locus (Chr16_678712) on BD3 (orange box plot), H3 (red) and the expression of *Potri.016G012400* (sky blue). Plots of correlation between each pair of BD3, H3 and the *Potri.016G012400* expression level among the genotype classes. The *r* value is based on a Pearson correlation coefficient. The *P*‐value is calculated using the *t* approximation. (g) Genotypic effect of the significant haplotype (TA‐CA‐T/T‐C‐A) on H2 (orange box plot), H3 (red) and *Potri.016G012400* expression (sky blue). Plots of correlation between H2 and H3 is drawn among the haplotype classes.

Within the upstream region of SHR21, a nonsynonymous SNP, Chr16_680030 (T/C, Leu > Pro), in exon 1 of *Potri.016G12400* (*DUF594*), is the lead SNP for BD3 (3‐year‐old basal diameter, *P *=* *1.48 × 10^−13^). This SNP shows obvious regional differentiation, and the mutant allele (C) was mainly distributed in S climatic regions in southern China (Figure S17), to confer preferentially higher radial growth of stem at the third years of growth stage (Figure 4c). Re‐sequencing found that an InDel polymorphism (Chr16_678712, CAA/C) within the 5′UTR of *DUF594* is most likely a causal variant, or is in high LD with the causal variants responsible for the natural variation of BD3 and H3 (3‐year‐old stem height), as well as *DUF594* expression (Figure 4d–f). Meanwhile, a synonymous variant in exon 1 of *DUF594*, Chr16_680796 (A/T), is the lead SNP for H2 (2‐year‐old stem height, *P *=* *1.40 × 10^−8^) and H3 (*P *=* *7.05 × 10^−9^) (Figure 4a–c). Further investigation showed SNP Chr16_680796 was in high LD (*r* ^2^ = 0.87–0.96, Figure 4e) with two adjacent InDels (Chr16_680791 and Chr16_680792, jointly caused the deletion of an amino acid) in exon 1, and were significantly associated with H2 and H3 (Figure 4b–d). Accordingly, a haplotype (TA‐CA‐T/T‐C‐A, Figure 4e) in exon 1 was significantly associated with H2 and H3, but did not cause differences in the expression of *DUF594* (Figure 4g). Our spatial expression patterns showed that *DUF594* is strongly expressed in the SAM, followed by the woody vascular cambium during the second and third years of growth in *P. tomentosa* clones (Data S14).

We also identified other significant variants linked to both novel genes in SHR21 that may underlie stem growth in the second and third year. For example, Chr16_736028 (G/A), a noncoding SNP variant located in intron 2 of a novel gene (*Potri.016G013200*), and partially overlapped with a cluster of lncRNA genes (*TCONS_00138008*), had the most significant association signals for V2 (2‐year‐old stem volume, *P *=* *1.03 × 10^−10^) and V3 (3‐year‐old stem volume, *P *=* *2.01 × 10^−15^) (Table 1 and Figure 4a,b). A similar scenario was observed for another novel gene *Potri.016G013500* (Chr16_754564 associated with BD2, *P *=* *1.97 × 10^−10^; Figure 4a,b)

**Figure 5:**
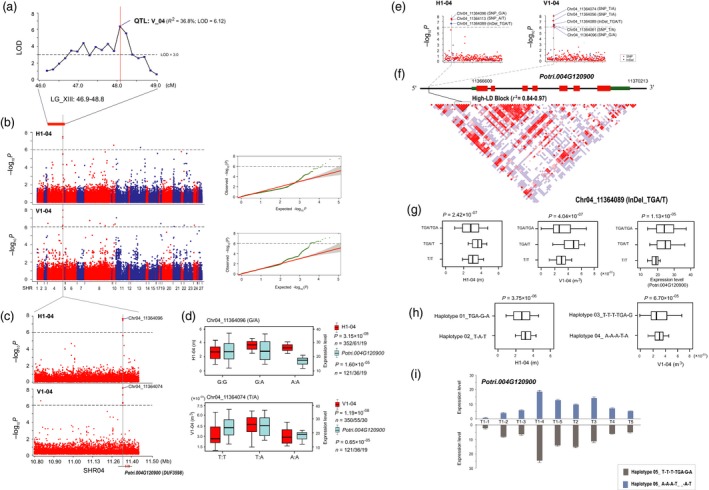
*DUF3598* variants in SHR04 may be growth‐stage‐specific loci in *Populus*. (a) The peak of a major QTL (LGXIII: 46.9‐48.8, *R* ^2^ = 36.8%), particularly for V_04 (the stem volume at the fourth timepoint), in the linkage population is indicated by a vertical red solid line. The dashed horizontal line depicts a uniform threshold of logarithm of odds (LOD) score for significant QTL (LOD score ≥ 3.0). (b) Manhattan and quantile‐quantile plots resulting from the association results between all SNPs of 27 SHRs and the H1‐04 and V1‐04 traits. The most significant locus is indicated by a vertical grey solid line. The *x*‐axis shows the SHR positions and the *y*‐axis shows the significance expressed as −log_10_ *P*‐value. The dashed horizontal line depicts the Bonferroni‐adjusted significance threshold (9.4 × 10^−7^). (c) The lead SNP (the SNP with the lowest *P*‐value) for each trait, whose position is indicated in the SHR04 locus by a larger red dot. *Potri.004G120900* (*DUF3598*) is shown at the bottom (red rectangle, coding sequences; black line, introns; green rectangle, 5′ and 3′ untranslated regions). (d) Box plot for H1‐04 (red), V1‐04 (red), and *DUF3598* expression (sky blue) is plotted as an effect of genotypes at the lead SNP. The horizontal line represents the mean and the vertical lines mark the range from the 5th and 95th percentile of the total data. (e) In the *DUF3598* locus, the most significantly associated SNPs (larger blue dots) and InDels (larger red dots) for H1‐04 and V1‐04 is indicated by a vertical grey solid line. (f) A linkage disequilibrium (LD) representation of the pair‐wise *r* ^2^ value among all polymorphic sites (SNPs and InDels) across the *DUF3598* locus, where the deeper red color of each box corresponds to the higher *r* ^2^ value. (g) Box plot for H1‐04, V1‐04, and *DUF3598* expression is plotted as an effect of genotypes at the potential InDel variant (Chr04_11364089, TGA/T) (h) Box plot for H1‐04 and V1‐04 is plotted as an effect of haplotypes at haplotypes 01‐02 (an InDel and two SNPs) and haplotypes 03‐04 (four SNPs and an InDel). (i) Bar plot for the expression level of haplotype 05 (grey) and haplotype 06 (sky blue) classes at the nine timepoints in shoot apex tissue of the poplar clones. Vertical lines represent the standard error.

For a major QTL (LGXIII: 46.9‐48.8) for V_04 (stem volume at the fourth timepoint) in the linkage population (*R* ^2^ = 36.8%, LOD = 6.12; Figures 1a and 5a), we further detected the corresponding SHR04 locus that significantly associated with H1‐04 (1‐year‐old stem height at the fourth timepoint) and V1‐04 during the first growing season in the association mapping panel (Figure 5b). A high‐LD block with five top‐ranked SNPs (*P *=* *3.16 × 10^−6^−1.19 × 10^−8^) was detected in the 2.5‐kb region upstream of *Potri.004G120900* (*DUF3598*) that significantly associated with V1‐04 (the lead SNP was Chr04_11364074, *P *=* *1.19 × 10^−8^) and/or H1‐04 (the lead SNP was Chr04_11364096, *P *=* *3.15 × 10^−8^) (Table 1 and Figure 5c–e). Both of these SNPs were associated with the expression of *DUF3598* with a similar allelic expression pattern (Figure 5d). Re‐sequencing also identified a potential InDel variant (Chr04_11364089, TGA/T) that was in high‐LD with five top‐ranked SNPs (*r* ^2^ = 0.84–0.97, Figure 5f), and was significantly associated with the natural variation of H1‐04 and V1‐04, as well as the differences in the expression of *DUF3598* (Figure 5g). Further analysis indicated that haplotype 01/02 (an InDel and two SNPs, *r* ^2^ = 0.89–0.95) significantly associated with H1‐04 (*P *=* *3.75 × 10^−06^, Figure 5e–h), and haplotype 03/04 (four SNPs and an InDel, *r* ^2^ = 0.84–0.97) significantly associated with V1‐04 (*P *=* *6.70 × 10^−05^, Figure 5e–h). This finding was verified by the haplotype‐specific expression pattern among the nine timepoints in the SAM of the haplotype 05/06 of poplar clones (Figure 5i), where *DUF3598* was preferentially expressed at the same fourth timepoint during the first growing season. Accordingly, *DUF3598* variants in SHR04 might be time‐specific loci acting in the first growing season in *Populus*.

### Interspecific expression profiles for potential time‐specific genes in the genus *Populus*

To test whether these potential time‐specific *P. tomentosa* genes have a stable temporal and spatial expression among the related species from the same genus, we selected five potential time‐specific genes (*WRKY3, DUF594, DUF3598, Potri.16G013200* and *Potri.16G073400*) from the *P. tomentosa* association panel to investigate the temporal and spatial expression profiles among five representative *Populus* species in the northern hemisphere (*P. euphratica, P. trichocarpa, P. tomentosa*,* P. tremula*, and *P. tremuloides*;* Salix purpurea* as the out‐group). As shown in Figure S19, the orthologs of the four novel genes were divided into three major classes of the phylogenetic tree (monocots, annual herbaceous dicots and perennial dicots) with well‐supported bootstrap values (Figure S19b–e). Five *Populus* orthologs were more closely related to orthologs from *Salix purpurea* in the phylogenetic tree than to each other from the perennial dicot subdivisions, suggesting that these novel genes originated from lineages that split off after Salicaceae speciation. All five genes exhibited distinct expression profiles within the vascular cambium and the SAM at the nine growth timepoints among five *Populus* species (Figure S19), and revealed that these potential time‐specific genes have a species‐specific expression pattern during the juvenile stages of stem growth.

## Discussion

Growth traits change throughout the organism's life cycle, and are excellent targets for understanding the temporal biological nature for growth, development and adaptation at the population levels. Here, we investigated the temporal genetic architecture of stem height and diameter during the juvenile growth stages by a linkage‐LD mapping approach in two well‐studied large populations of *Populus*. Our investigation provides insights into the genetic basis of stem growth in *Populus* (Figures 1, 3 and S12), and also helps to uncover potential time‐specific genes and favourable alleles (Table 1 and Figures S15–19). Henceforth, our work may serve as a rough guideline to drive the dissection of quantitative trait dynamics in trees.

### The linkage‐LD mapping method enables the dissection of the dynamic genetic basis of stem growth in trees

For a long‐lived tree species, the first‐growth season is one of the key fundamental stage of a tree's life cycle, which determines the survival and the spatial advantage of each individual in a population (Hulshof *et al*., 2015). In general, the stem height–diameter growth dynamics over the first‐growth season is a biological predictor of plant growth advantage and fitness. In the linkage population, 1,200 *Populus* progeny showed considerable variation in the stem H/D growth trajectory at the first‐growth‐season (Figure S2a), suggesting that juvenile stem growth involves multiple QTL and numerous genes (Yamaguchi *et al*., 2009).

For our linkage‐LD mapping strategy, linkage mapping benefits from high statistical power due to many individuals sharing the homogeneous genotypic compositions, and can effectively examine all segregating intervals with functional loci associated with variation for target traits (Motte *et al*., 2014; Sterken *et al*., 2012). These dynamic QTL were categorised into 42 discrete overlapping regions (Figure 1a), which presumably result from the joint effects of closely linked minor‐to‐moderate QTL or the pleiotropy of major regulators throughout the growth periods. Some general QTL underlying stem growth traits were detected at all investigated timepoints, whereas others were specific to one or a few particular timepoints (Figure 1a), supporting the idea that the genetic system of stem growth is not static and is involved in the coordinated activity of multiple interacting variants throughout the life cycle (Bac‐Molenaar *et al*., 2015; Meijon *et al*., 2014). Several studies in which mapping of quantitative data collected over time have been reported in herbaceous plants, resulted in the identification of time‐specific QTL for root bending upon a change in the direction of gravity in *Arabidopsis* (Moore *et al*., 2013) and for plant height in wheat (Wurschum *et al*., 2014).

The relatively good collinearity and conserved gene order between the linkage map and the reference genome makes the application of linkage‐LD mapping for high‐resolution allelic dissection of the dynamic QTL very promising (Du *et al*., 2016). Here, this joint approach not only reveals many QTL regions present in species for dynamic processes of complex traits, but also identifies important anchor points within QTL for the observed temporal variation. Our association mapping within QTL provides inter‐population validation and allows dissection of causative genes for these dynamic QTL over a longer growth period (Figures S6–S8), indicating the linkage‐LD mapping strategy is both statistically powerful and biologically relevant for the understanding of dynamic genetics on quantitative traits. Numerous well‐characterised loci (approximately 6.1%–31.4% for a certain trait) over a longer period of growth repeatedly contributed to the other two traits at multiple growth phases. It makes biological sense that stem growth is a product of all the compositional traits combined, and many significant SNPs could play sustaining pleiotropic roles for this process (Bac‐Molenaar *et al*., 2015). The relative effect size of these regulators might change over time (Figure S12) as a result of the dynamic balance between different regulatory components during development pathways (Mizrachi *et al*., 2017). Our results showed that biological dissection of stem growth over time can identify the underlying genetic factors, and is more powerful than the evaluation of growth by single‐point measurements (Bac‐Molenaar *et al*., 2015; Meijon *et al*., 2014).

The rationale behind this linkage‐LD mapping approach is that the colocalisation of significantly associated peaks in SHRs with certain QTL intervals can help to find the positive genes/variants, providing direct evidence that linkage‐LD mapping is not hypothesis‐driven, but rather data‐driven (Motte *et al*., 2014). Some well‐characterised QTL in a linkage population do not co‐localise with the highest association peaks for the same growth phase or the same traits (Figures 1a and S9), owing to the confounding effects between the genetics and the local environments. It is presumed that the functional alleles at these loci occur at a different frequency in linkage and association mapping panels (Figure S11), implying that some QTL/genes affecting quantitative variation might be independent of genetic background (Ogbonnaya *et al*., 2017).

### Local adaptation of candidate genes within overlapping selection and association regions of QTL

The stem height–diameter relationship is a predictor of geographical distribution and local adaptation of unrelated individuals in widespread distributions (Hulshof *et al*., 2015). Considerable differences in stem height and diameter at the beginning of the first growing season occurred among the three climatic regions of *P. tomentosa* (Figure S2b), suggesting that the phenotypic plasticity of stem growth in forest trees varies substantially along spatial and environmental gradients. In our study, stem growth experienced selection pressure responsible for local adaptation among different climatic regions of *P. tomentosa*. For example, the S climatic region (with a high H/D, Figure S2b), located in low latitude and semi‐humid areas of southern China, might represent the centre of origin of this poplar species (Du *et al*., 2012). Local individuals typically exhibited vigorous height growth, which helped the seedlings to quickly reach light above the canopy at an early stage period (Poorter *et al*., 2003). In contrast, poplar trees in the NW region (with a lower H/D, Figure S2b), located in high altitude, arid and semiarid areas that frequently experienced low temperatures and limited annual rainfall (Du *et al*., 2012; Huang, 1992), might have evolved stouter stems and crowns to withstand those extreme climates compared to the same species in lower altitude areas (Feldpausch *et al*., 2011; Hulshof *et al*., 2015). Dynamic variation in stem apical and radial growth among different climatic regions (Table S9 and Figure S2b) supports that a varied number of potential selective signals within the 27 SHRs were detected for three pairwise climatic groups (Figure 2). These genomic regions/genes are likely to be driven by local selection responsible for these adaptive stem growth traits (Wang *et al*., 2011).

Functional categories for 104 potential selective signals (Table S5) that are expected to represent genomic targets of selection for complex biological pathways underlying stem growth, supporting that PCGs within these regions contain a variety of annotations plausibly related to cell wall, transcriptional regulation and stress response. Such genes may provide excellent targets for natural selection and for functional studies aimed at elucidating the drivers of local adaptation in stem growth in *Populus* species. Remarkably, we found that those selective genes had partially stronger association signals associated with dynamic stem growth in the association mapping panel (Figure S16c and Table 1), which was consistent with our observation that the allelic distributions of most potential major SNPs exhibited significant frequency differences between the climatic regions (Figure S17). Overlap between associated signals and selective signatures may suggest that the selected genes and alleles may be involved in the biological adaptation of juvenile stem growth. Similar ‘landscape genomics’ research areas have been reported in the annual plant *Medicago truncatula* (Guerrero *et al*., 2018), *Triticum urartu* (Brunazzi *et al*., 2018) and undomesticated woody species, which can provide further insight into the potential genes and mechanisms of adaptation to the environment.

More specifically, the lead SNPs from *LOB42*,* GLR2.7*,* LRR‐RLK*,* JAG*,* PPC4* and an unknown PCG (*Potri.016G073400*), showed potentially similar regional differentiation patterns, as the mutant allele of each SNP increased in frequency from the S to the NW or NE regions (Figure S17). These mutant alleles are likely to jointly participate in determining dynamic variation in stem apical and radial growth in the high‐latitude, arid environments to maximise trees fitness. This observation suggests that these selective genes perhaps have undergone similar selection in response to environmental change and evolutionary processes. We also found the lead SNP within *DUF594* showed an opposite pattern with the above variants, where the mutant allele T was more likely to found in high frequency in the low latitude S region (Figure S18). This observation supports that the preferential allele of *DUF594* modulates the higher stem radial growth in the third year of growth (Figure 4c), suggesting that more rapid radial stem growth can facilitate the maintenance of competing advantage after individuals obtain a space in the first growth season (Hulshof *et al*., 2015). These SNPs occur at high frequency in the isolated geographical populations, and could be considered as candidates for regional breeding programmes.

### Interpretation of possible causative genetic factors underlying specific growth phases

In our study, one main focus was on the identification of stage‐specific QTL and the favourable variants underlying stem growth. More specifically, we found some candidate genes involved in specific growth phases and investigated their potential temporal effects. None of these candidates have yet been reported to be related to growth dynamics.

In SHR04, we detected a possible time‐specific gene *WRKY3,* in which a nonsynonymous variant of SNP Chr04_11343447 (T/C), is the major SNP for BD and V traits at the four and 5‐year‐old growth stages (Data S11). These findings were supported by the expression profiles showing that *WRKY3* expression was higher in the vascular cambium than in the SAM of the 4‐ to 5‐year‐old *P. tomentosa* clones (Data S14), suggesting that the WRKY3 TF is temporally involved in stem growth, mostly in radial secondary growth rather than in the apical meristems of *P. tomentosa*. Therefore, additional temporal growth and gene expression data could be collected to determine whether the candidate genes play a time‐specific or a general role in plant growth regulation. *WRKY*s, as important regulators of abiotic stress tolerance, might play a pivotal role in plant growth (Rushton *et al*., 2012). Other direct evidence also showed the mutation of *WRKY* initiates pith secondary wall formation and increases stem biomass in dicotyledonous plants (Wang *et al*., 2010). More stage‐specific information can help to prioritise candidate genes for future functional analyses. For example, poplar *WRKY3* has a species‐specific expression pattern during the juvenile stages of stem growth.

In SHR08, we identified a lead SNP Chr08_7871772 (A/T), located 138 bp upstream of *Potri.008G121100* (encoding a ribosomal protein L15, RPL15), which had the highest association signal for H1‐05 (1‐year‐old stem height at the fifth timepoint), and also as a significantly top‐ranked locus for H1‐04 and H1‐03 (Table 1 and Figure S16). *RPL15* encodes a protein component of ribosomes that exhibit various secondary functions in DNA repair, apoptosis, abiotic resistance and cell proliferation (Barakat *et al*., 2001), and may be a key regulator on the biological pathway involving in stem growth. The tissue‐specific expression pattern of *RPL15* in our study showed higher expression in the SAM than in the vascular cambium at the third to fifth timepoints during the first year of growth (Data 14), which supported that the *RPL15* variant significantly associated with 1‐year‐old stem height at three continuous timepoints (Figure S16b). The preferential expression of *RPL15* in the SAM may directly contribute to stem height at the beginning of the first year of growth in poplar. Our knowledge of stem growth dynamics is now improved by our linkage‐LD mapping, largely due to the identification of novel genes without functional annotation based on the *P*. *trichocarpa* genome. For example, we consider three novel genes (*DUF594*,* Potri.016G013200* and *Potri.016G013500*) with associated variants in SHR21 (Figure 4a–c). Re‐sequencing improves our understanding of the causative variants/genes underlying stem growth, potentially supporting our observation that an InDel polymorphism within the 5′UTR of *DUF594* is most likely the causal variant, or is in high LD with the causal variants responsible for the natural variation of BD3 and H3, as well as *DUF594* expression (Figure 4d–f). The spatial expression patterns showed that *DUF594* is strongly expressed in the SAM, followed by the woody vascular cambium during the second and third years of growth in *P. tomentosa*, which was consistent with the interspecific expression profiles in the SAM of several poplar species (Figure S19), suggesting that *DUF594* may regulate the primary growth process specifically associated with tree height during the second and third years of growth.

Functional annotation of orthologs of these novel poplar genes in other model species would improve our understanding of their functions. For example, *Potri.016G013200*, a novel gene in the SHR21 locus, was detected as the most significant association signal for V2 and V3 (Figure 4b,c). This gene has a high sequence identity (53.0% at the amino acid level) with a transmembrane protein in *Arabidopsis*. Functional annotation of this gene may elucidate the genic variants underlying the dynamics of V2 and V3 growth, which was supported by the peak expression identified in the vascular cambium tissue of all five *Populus* species during the second and third years of growth (Figure S19). Additionally, a noncoding SNP located in the 5′UTR of *Potri.016G013500*, another novel gene with an unknown domain in SHR21, had a significant association signal with BD2 (Figure 4a,b). *Potri.016G013500* has a high sequence identity (55.0% at the amino acid level) with an *Arabidopsis* gene (*AT5G20130*). *AT5G20130* encodes an enzyme of the sulphate adenylyltransferase subunit, which is part of a bifunctional polypeptide chain associated with the adenosyl phosphosulphate (APS) kinase that catalyses the primary step of intracellular sulphate activation (Ullrich *et al*., 2001).

### Further perspectives on the dissection of quantitative trait dynamics in perennial woody species

We characterised the epistatic effects of significant loci that exhibit complex allelic epistasis patterns over dynamic growth periods of poplar in a quantitative manner (Figure 3), suggesting that non‐additive epistasis, in addition to main‐effect additive marker loci, co‐ordinately act in a growth phase‐specific manner (Mackay, 2014; Phillips, 2008). Although our analyses indicate that epistasis is a common phenomenon underlying dynamic growth processes, detecting which of the statistically predicted interacting variants are biologically important to specific growth stages using more approaches remains challenge. Epistasis might better explain the minor effects, missing heritability and the absence of replication of causal variants in different populations through genome‐wide association studies (GWAS) (Manolio *et al*., 2009). Additionally, attention should be paid to understanding the functional basis of gene/allele epistasis at a genome‐wide scale in different populations and could improve our understanding of the roles of epistasis in the geographical and climatic adaptation of long‐lived woody species.

Our work using association genetics provides novel insights into the genetic architecture underlying dynamic growth processes of poplar. This approach can be extensively used in future work aimed at understanding tree quantitative traits. However, the conventional phenotyping procedures used for our populations, which are labour‐intensive, time‐consuming and costly for most plant species, represent a ‘phenotyping bottleneck’ (Furbank and Tester, 2011). In the emerging era of phenomics and genomics, an automated tree phenotyping platform is needed for collecting large‐scale phenotypic data for multiple populations under different environments over long periods. The resolution and precision should be higher than the current phenotyping systems developed for rice and *Arabidopsis* (Meijon *et al*., 2014; Slovak *et al*., 2014; Yang *et al*., 2014). Therefore, using a genomics‐to‐phenomics platform could help identify the timing of growth phase changes and quantify the temporal mechanisms of variants to determine when and how a phase change occurs in tree species, to finally use for molecular designing of tree ideotypes.

## Experimental procedures

#### Population materials and DNA extraction

The linkage population consisted of 1,200 hybrid poplar trees, and was established in 2008 by controlled crossing between two closely related *Populus* species, clone ‘YX01’ (*Populus alba *× *P*. *glandulosa*) as the female parent and clone ‘LM50’ (*P. tomentosa*) as the male parent (Du *et al*., 2016). In 2011, the 1,200 progeny were asexually propagated for QTL mapping in the Xiao Tangshan horticultural fields, Beijing, China (40°2′N, 115°50′E), using a randomised complete block design with three replications.

An association mapping panel of 435 5‐year‐old, unrelated individuals, were asexually propagated in 2011 in Guan Xian County, Shandong Province, China (36°23′N, 115°47′E), which were randomly sampled from a collection of 1,047 natural *P*. *tomentosa* individuals in the same fields (Du *et al*., 2014), representing almost the entire natural distribution of *P*. *tomentosa* (30–40°N, 105–125°E). The distribution zone from which these samples were collected was divided into the southern (S, *n *=* *180), north‐western (NW, *n *=* *95), and north‐eastern (NE, *n *=* *160) climatic regions (Huang, 1992). Total genomic DNA was extracted from fresh leaves of each individual using the DNeasy Plant Mini kit (Qiagen, Shanghai, China) following the manufacturer's protocol.

#### Measurement of dynamic growth traits

Three stem growth traits, H, BD and V, were measured at multiple timepoints during field surveys using standard procedures described by Du *et al*. (2014) with at least three measurement replications per sample. For the linkage population, we measured the three growth traits at 12 timepoints (each timepoint was 15 days apart, totalling 180 days, T_01 to T_12) throughout the first growing season. The first measurements were conducted on May 10th, 2011 when the third leaf began growing and the successive measurements were performed once every 15 days thereafter until most individuals in the population reached bud set. For the association mapping panel, measurements were taken at five successive timepoints, T1‐01 to T1‐05 (each timepoint was 36 days apart, totalling 180 days), throughout the first growing season beginning on May 10th, 2011 in the Guan Xian County. Thereafter, these 435 individuals were annually measured for the three growth traits from 2012 to 2015 (T2 to T5). Analysis of variance (ANOVA) was performed with the software SAS for Windows, ver. 8.2 (SAS Institute, Cary, NC, USA).

#### QTL analysis

A high‐resolution linkage map has been constructed from the linkage population, which includes 1,270 markers (929 AFLPs, 309 SSRs and 32 InDels) in 19 LGs with an average marker interval of 2.3 cM (Du *et al*., 2016). The detailed procedure on marker genotyping and linkage map construction was described in Du *et al*. (2016). The number of LGs correspondingly equals the number of haploid chromosomes in *Populus* (Du *et al*., 2016). QTL analysis for three growth traits at twelve timepoints was conducted in MapQTL 6.0 software (Van Ooijen, 2009). To determine a uniform threshold of LOD score for significant QTL, 1,000 permutations tests were used for each trait with a genome‐wide error rate of 5% (Churchill and Doerge, 1994) and the average LOD significant threshold value was 3.0 for all 36 traits at *P *<* *0.05, so we chose a uniform value (LOD = 3) as the cut‐off. The potential QTL were initially detected employing the Interval Mapping algorithm. Then, markers with the highest LOD value were selected as cofactors and the final set of markers associated at *P *<* *0.02 after automatic cofactor selection were further implemented in the Multiple QTL Model (MQM). Co‐factor selection and MQM analysis were repeated until the best possible set of QTL was declared, then each QTL was characterised by its maximal LOD score and PVE. The QTL confidence interval was designated in centiMorgans (cM) and corresponded to a LOD score (>3.0) drop of one on either side of the likelihood peak (Visscher *et al*., 1996). A discrete co‐localisation region was defined if the confidence interval of each QTL overlapped or partially overlapped the same LG, and the position of all co‐localisation QTL peaks was <1.0 cM apart (Tables S2 and S3).

#### Detection of QTL hotspots and analysis

To identify significant QTL hotspots, we permutated the total QTL peak counts per cM interval for three growth traits at 12 timepoints, across a total map distance of 2,758.6 cM of the linkage map 1,000 times (Du *et al*., 2016), and determined the 95th percentile of these permutations. Each interval with a total absolute QTL peak count greater than this permutation threshold was declared a QTL hotspot.

#### Identification of the putative SHRs of the QTL

Comparison of the linkage map and the *P. trichocarpa* reference genome revealed relatively strong conservation of gene order (Du *et al*., 2016). The putative SHRs were identified on the annotated *Populus* reference genome v3.0 (http://popgenie.org/) corresponding to the confidence interval of each QTL, based on a comparison of the anchoring gene‐derived markers on the linkage map with the reference genome using BLASTX with a cut‐off *E*‐value < 1.0e‐10 (Du *et al*., 2016). The corresponding genomic size of each SHR was calculated by the map distances and genomic sequence length of at least three anchoring gene‐derived markers within or near the QTL (Du *et al*., 2016).

#### Annotation of PCGs and GO analysis within the SHRs

The PCGs within the SHRs were annotated on the basis of the gene models of the *Populus* reference genome v3.0. GO terms were determined by AgriGO (http://bioinfo.cau.edu.cn/agriGO/index.php), and the FDR (false discovery rate) < 0.05 was set as threshold to identify significant GO terms.

#### Identification and target prediction of noncoding RNA genes

The pre‐miRNA and lncRNA genes within the SHRs were annotated based on our transcriptome database of predicted lncRNAs and miRNAs in *P. tomentosa* as previously described (Chen *et al*., 2015; Tian *et al*., 2016; Zhou *et al*., 2017). In total, 430 lncRNAs were derived from 121 overlapping genomic amplicons, and include 425 sense lncRNAs and five intergenic lncRNAs (Data S2). Prediction of miRNA target genes was performed by psRNATarget (http://plantgrn.noble.org/psRNATarget/) via complementary base pair interactions, with expectation ≤ 5.0. Two independent algorithms were used to predict the potential target genes of lncRNAs according to their *cis*‐ and *trans*‐ regulatory actions, and the detailed methods are provided in Method S1.

#### SNP genotype calling in the association mapping panel using re‐sequencing

All 435 unrelated individuals were sequenced with an average of 15× raw data coverage using the Illumina GA2 instrument, and the library construction for genome re‐sequencing follows the manufacturer's recommendations (Illumina), which are provided in Method S2. The quality of paired‐end short reads of 100 bp or 150 bp was controlled by removing low‐quality reads (≤50% of nucleotides with a quality score < Q20) (Du *et al*., 2016). The short reads were mapped and aligned to the *Populus* reference genome v3.0 using SOAPaligner (SOAP2, v2.20) with the default options (Li *et al*., 2008).

To obtain high‐quality SNPs, we selected the reads that could be matched to a unique genomic location to perform SNP calling. The genotype likelihood of the genomic site for each *Populus* individual was calculated using SOAPsnp v1.03 with default parameters (Li *et al*., 2009), which generated a data set of all genotypes. UltraEdit software v3.2 (http://www.ultraedit.com/) was used to capture the genotype data of 106 663 candidate SNPs within the 27 SHRs (Table S3) following the SNP calling pipeline. Low‐quality SNPs were removed in TASSEL v5.0 (http://www.maizegenetics.net/tassel) with the selection criteria of missing data > 10% and MAF < 0.05. All high‐quality SNPs within the SHRs were assigned as noncoding RNA‐derived SNPs, genic (noncoding, synonymous and nonsynonymous), or intergenic annotation sets based on their locations relative to our annotated PCGs and noncoding RNA genes. To include SNPs in the promoter or flanking regulatory regions for each gene, we included the full‐length gene sequences 2 kb upstream and downstream of each gene.

#### Estimation of population parameters

The average number of pairwise differences per site between any two sequences, π (Nei, 1987), and the number of segregating sites, θ_w_ (Watterson estimator, Watterson, 1975), were used for nucleotide diversity calculations. Also, Tajima's D (Tajima, 1989) for neutrality tests was estimated using genotypic data after screening missing values. The population‐differentiation statistic (Fst) was measured for each pairwise region differentiation based on genetic polymorphism data throughout the SHRs (Danecek *et al*., 2011). More details on the statistics formula on Fst, θ_w_, π and Tajima's D calculations are provided in Method S3. These population parameters were calculated in 250‐bp sliding windows within the SHRs according to Schmutz *et al*. (2014), and these haplotype‐based estimates can be used for selective signature screening within SHRs.

#### Detection of signatures of putative regions under selection

We used the top 1% of the empirical distribution of Fst and π values within the SHRs as candidates representing signatures of significant divergent selection between each pairwise climatic region (NW vs. NE, NW vs. S and NE vs. S), in which the latter was the reference population and the former was the object population. Selection sweeps were evaluated using an adjusted method for reduction of diversity (ROD) statistics according to Xu *et al*. (2011), by using 250‐bp non‐overlapping windows along all the SHRs and an extensive 10‐kb region upstream and downstream of each SHR. All genes in the selective sweep regions were identified as putative genes under selection.

#### Linkage disequilibrium analysis

The squared allele‐frequency correlations (*r* ^2^) values across pairs of SNP loci within each SHR were calculated in TASSEL v5.0 with 10^5^ permutations. A visual software package, Haploview v4.2 (Barrett *et al*., 2005), was used to detect the high‐LD haplotype blocks within each SHR. When calculated across the entire association panel, the decay of LD with physical distance (bp) between the common SNPs for each SHR was estimated separately by nonlinear regression.

#### Association mapping to detect the additive and dominance effects of allelic SNPs

We carried out a compressed MLM procedure (Yu *et al*., 2006) implemented in TASSEL v5.0, to examine the main effect (additive and/or dominance) of each SHR‐derived SNP (Du *et al*., 2016). The significant thresholds of the association analysis were determined using a modified Bonferroni correction (Li *et al*., 2012), in which the significance was first defined at a uniform threshold of = 1.9 × 10^−5^ (suggestive *P *=* *1/n; *n* = total markers used in association studies, 52 949 SNPs, which is roughly a Bonferroni correction), according to Wen *et al*. (2014). Also, a more stringent threshold of *P *=* *9.4 × 10^−7^ (significant *P *=* *0.05/*n*) was subsequently calculated on the basis of an empirical estimate of the number of independent tests for the entire population, based on a nominal level of 0.05 (Li *et al*., 2012). To uncover the unique candidate gene underlying association signals, we performed LD analysis of SNPs with the lowest *P*‐value (lead SNP) within each significant SHR locus and used a cut‐off of <0.2 for the LD statistic *r* ^2^ (Li *et al*., 2013). Among the unique association signals identified, several candidate genes in or near (within 10 kb up‐ and downstream of the lead SNP) known genes were validated.

#### Epistasis analysis between each SNP pair

The EPISNP v2.0 module integrated in epiSNP windows software package (Ma *et al*., 2008), was used for genome‐wide testing of the epistatic effects of locus pairs on complex quantitative traits. In this programme, an extended Kempthorne model (Mao *et al*., 2007) was implemented for estimating epistatic effect (two‐locus interaction) between each marker pair. The contribution of each epistatic pair to phenotypic variation was calculated following the formula: c = *SS*_*SNP1 × SNP2*_ */Var*_*p*_, where *SS*_*SNP1 × SNP2*_ was the variance of the significant *SNP1× SNP2* interactive effect and the *Var*_*p*_ was the phenotype variance. More details about this module are described in Ma *et al*. (2008).

#### Real‐time quantitative PCR (RT‐qPCR)

Expression analysis was conducted to investigate the tissue or temporal expression patterns of the potentially associated genes in the population of *P. tomentosa* and four other species within the genus (*P. euphratica, P. trichocarpa, P. tremula* and *P. tremuloides*). Each pool was a mixture of the same tissue samples from 10 random *P. tomentosa* individuals at a particular timepoint. The details of total RNA extraction, cDNA synthesis and RT‐qPCR programme and reaction are described in Methods S4. The primer pairs for the tested genes and the internal control (*Actin1*, Accession number: EF145577) are listed in Table S10. The expression values were analysed using Opticon Monitor Analysis Software v3.1 and standardised to the levels of *Actin1* using the 2^−ΔΔCt^ method. All reactions were performed with three technical replications and three biological replications.

#### SNP genotype‐specific expression analysis

To test whether the most significant SNPs and InDels affect the relative transcript abundance of their corresponding genes, we quantified the mRNA levels of the different SNP and InDel genotypic classes in the association mapping panel by RT‐qPCR. The tested timepoints and tissues were selected based on the spatial association signals; the stem vascular cambium was generally used for validating the variants underlying BD traits and SAM was used for variants associated with H. For each genotypic class, tens or hundreds of individuals from the association mapping panel were sampled by obtaining cDNA from the fresh stem cambium or SAM tissue.

## Accession numbers

Raw sequence data of genome re‐sequencing have been deposited in the Genome Sequence Archive in BIG Data Center, Beijing Institute of Genomics (BIG), Chinese Academy of Sciences, under accession number CRA000903 that is publicly accessible at http://bigd.big.ac.cn/gsa. All data corresponding to the QTL and linkage disequilibrium mapping are provided in Supplementary Tables and Data.

## Author contributions

D.Z. planned and designed the research. Q.D., X.Y., J.X., M.Q., L.X., W.L., J.T., C.G., J.C., and B.L. performed experiments, conducted fieldwork, analysed data etc. Q.D. wrote the manuscript.

## Supporting information


**Figure S1** Growth trajectories of tree height, basal diameter and stem volume in *Populus* linkage population and association mapping panel.
**Figure S2** The dynamic curves of the stem height to diameter ratio in the *Populus* linkage population and association mapping panel.
**Figure S3** Annotation and target gene prediction within all 27 putative SHRs.
**Figure S4** Estimates of the squared allele‐frequency correlations for all pairwise SNP combinations within each SHR.
**Figure S5** The diverse patterns of linkage disequilibrium decay with physical distance between the common SNPs separately for each SHR.
**Figure S6** Manhattan and quantile‐quantile plots resulting from the association results between all SNPs of 27 SHRs and basal diameter at the nine timepoints.
**Figure S7** Manhattan and quantile‐quantile plots resulting from the association results between all SNPs of 27 SHRs and stem height at the nine timepoints.
**Figure S8** Manhattan and quantile‐quantile plots resulting from the association results between all SNPs of 27 SHRs and stem volume at the nine timepoints.
**Figure S9** The significance of 27 SHR‐wide scans for basal diameter, tree height and stem volume at the nine growth timepoints of *Populus*.
**Figure S10** Structural networks that represent significant gene, lncRNA and miRNA loci for basal diameter, tree height and stem volume at the nine growth timepoints of *Populus*.
**Figure S11** Identification of the association hotspots related to the protein‐coding genes and noncoding RNA genes at the suggestive and significant *P*‐values respectively.
**Figure S12** The genetic effect and phenotypic contributions of Chr04_11343447 and Chr16_5505403 over the 2‐ to 5‐year growth phases.
**Figure S13** Gene–gene interactions formed uniquely interconnected networks for basal diameter at the nine timepoints.
**Figure S14** Gene–gene interactions formed uniquely interconnected networks for tree height at the nine timepoints.
**Figure S15** Gene–gene interactions formed uniquely interconnected networks for stem volume at the nine timepoints.
**Figure S16** SHR08 contains causative signals underlying growth‐stage‐specific stem height and basal diameter growth.
**Figure S17** The allelic distributions of 12 lead SNP sites among individuals from the three climatic regions.
**Figure S18** SHR23 may contain a causal SNP (Chr16_5505403) associated with the fourth and fifth year of growth in *Populus*.
**Figure S19** Interspecific expression profiles for the five potential time‐specific genes in the genus *Populus*.
**Table S1** Descriptive statistic for each trait at each single timepoint in the linkage population and association mapping panel.
**Table S2** Quantitative trait locus detection and segmental homology region identification for three growth traits at 12 timepoints in a *Populus* interspecific linkage population.
**Table S3** Annotation of dynamic QTL and corresponding segmental homology regions for three growth traits over 12 timepoints in *Populus*.
**Table S4** Annotation and diversity assessment of genes and SNPs within 27 segmental homology regions in the association mapping panel of *Populus*.
**Table S5** Summary of potential selective signals and genes within the SHRs in the association mapping panel.
**Table S6** Functional annotations of these selective protein‐coding genes among all pairwise climatic regions of *Populus* (NW vs. S, NE vs. S or NW vs. NE).
**Table S7** Summary of the number of all significant SNP within SHRs associated with three growth traits over nine time points of *Populus*.
**Table S8** Summary of pairwise SNP and gene epistasis for three traits at nine time points in association mapping panel of *Populus*.
**Table S9** The mean value for each trait at the nine time point in the three climatic regions of the association mapping panel.
**Table S10** Primers used for reverse transcription quantitative PCR for the candidate genes.
**Method S1** Identification and target prediction of noncoding RNA genes.
**Method S2** Library construction for genome re‐sequencing.
**Method S3** Statistics formulas for Fst, θ_w_, π and Tajima's D calculations.
**Method S4** Total RNA extraction, cDNA synthesis and reverse transcription quantitative PCR.

**Data S1** Annotation of all protein‐coding genes in the 27 SHRs of *Populus*.
**Data S2** Summary of all long noncoding RNA genes in the 27 SHRs of *Populus*.
**Data S3** Summary of all MicroRNA genes in the 27 SHRs of *Populus*.
**Data S4** Gene ontology (GO) term analysis of all genes in the 27 SHRs at the top‐ranked enrichments (FDR ≤ 0.05).
**Data S5** Prediction of lncRNA‐target gene pairs within all 27 SHRs.
**Data S6** Prediction of microRNA‐target gene pairs within all 27 SHRs.
**Data S7** Summary of all SNP variants in the association mapping panel of *Populus*.
**Data S8** Summary of potential selective signals within the SHRs among pairwise climatic regions of *Populus*.
**Data S9** Summary of potential selective genes within the SHRs among pairwise climatic regions of *Populus*.
**Data S10** The allelic distributions of 109 representative SNPs within the selective genes among the three climatic regions.
**Data S11** Identification of significant additive and dominance associations for BD, H and V at the nine timepoints.
**Data S12** Significant SNP–SNP epistatic interactions for BD, H and V across the nine timepoints in the association mapping panel.
**Data S13** Pairwise epistatic interactions between these significant additive/dominant SNPs detected for V and BD at multiple timepoints.
**Data S14** Expression data of several time‐specific genes at the corresponding timepoints or tissues in *Populus*.
