# Supplementary material for: Time‐specific and pleiotropic quantitative trait loci coordinately modulate stem growth in Populus
Source: Plant Biotechnol J. 2018 Sep 17;17(3):608–24. doi: 10.1111/pbi.13002 (PMC6381792; doi:10.1111/pbi.13002)
Supplement: Supplementary file 1 — Figure S1 Growth trajectories of tree height, basal diameter and stem volume in Populus linkage population and association mapping panel. Figure S2 The dynamic curves of the stem height to diameter ratio in the Populus linkage population and association mapping panel. Figure S3 Annotation and target gene prediction within all 27 putative SHRs. Figure S4 Estimates of the squared allele‐frequency correlations for all pairwise SNP combinations within each SHR. Figure S5 The diverse patterns of linkage disequilibrium decay with physical distance between the common SNPs separately for each SHR. Figure S6 Manhattan and quantile‐quantile plots resulting from the association results between all SNPs of 27 SHRs and basal diameter at the nine timepoints. Figure S7 Manhattan and quantile‐quantile plots resulting from the association results between all SNPs of 27 SHRs and stem height at the nine timepoints. Figure S8 Manhattan and quantile‐quantile plots resulting from the association results between all SNPs of 27 SHRs and stem volume at the nine timepoints. Figure S9 The significance of 27 SHR‐wide scans for basal diameter, tree height and stem volume at the nine growth timepoints of Populus. Figure S10 Structural networks that represent significant gene, lncRNA and miRNA loci for basal diameter, tree height and stem volume at the nine growth timepoints of Populus. Figure S11 Identification of the association hotspots related to the protein‐coding genes and noncoding RNA genes at the suggestive and significant P‐values respectively. Figure S12 The genetic effect and phenotypic contributions of Chr04_11343447 and Chr16_5505403 over the 2‐ to 5‐year growth phases. Figure S13 Gene–gene interactions formed uniquely interconnected networks for basal diameter at the nine timepoints. Figure S14 Gene–gene interactions formed uniquely interconnected networks for tree height at the nine timepoints. Figure S15 Gene–gene interactions formed uniquely interconnected networks for stem volu [file PBI-17-608-s002.doc]

## Supporting Information

Article title: **Time-specific and pleiotropic quantitative trait loci coordinately modulate stem growth in *Populus***

Authors: Qingzhang Du, Xiaohui Yang, Jianbo Xie, Mingyang Quan, Liang Xiao, Wenjie Lu, Jiaxing Tian, Chenrui Gong, Jinhui Chen, Bailian Li, and Deqiang Zhang*

The following Supporting Information is available for this article:

**Figure S1** Growth trajectories of tree height, basal diameter, and stem volume in *Populus* linkage population and association mapping panel.

**Figure S2** The dynamic curves of the stem height to diameter ratio in the *Populus* linkage population and association mapping panel.

**Figure S3** Annotation and target gene prediction within all 27 putative SHRs

**Figure S4** Estimates of the squared allele-frequency correlations for all pairwise SNP combinations within each SHR.

**Figure S5** The diverse patterns of linkage disequilibrium decay with physical distance between the common SNPs separately for each SHR.

**Figure S6** Manhattan and quantile-quantile plots resulting from the association results between all SNPs of 27 SHRs and basal diameter at the nine timepoints.

**Figure S7** Manhattan and quantile-quantile plots resulting from the association results between all SNPs of 27 SHRs and stem height at the nine timepoints.

**Figure S8** Manhattan and quantile-quantile plots resulting from the association results between all SNPs of 27 SHRs and stem volume at the nine timepoints.

**Figure S9** The significance of 27 SHR-wide scans for basal diameter, tree height, and stem volume at the nine growth timepoints of *Populus*.

**Figure S10** Structural networks that represent signiﬁcant gene, lncRNA, and miRNA loci for basal diameter, tree height, and stem volume at the nine growth timepoints of *Populus*.

**Figure S11** Identification of the association hotspots related to the protein-coding genes and noncoding RNA genes at the suggestive and significant *P*-values, respectively.

**Figure S12** The genetic effect and phenotypic contributions of Chr04_11343447 and Chr16_5505403 over the two- to five-year growth phases.

**Figure S13** Gene–gene interactions formed uniquely interconnected networks for basal diameter at the nine timepoints.

**Figure S14** Gene–gene interactions formed uniquely interconnected networks for tree height at the nine timepoints.

**Figure S15** Gene–gene interactions formed uniquely interconnected networks for stem volume at the nine timepoints.

**Figure S16** SHR08 contains causative signals underlying growth-stage-specific stem height and basal diameter growth.

**Figure S17** The allelic distributions of 12 lead SNP sites among individuals from the three climatic regions.

**Figure S18** SHR23 may contain a causal SNP (Chr16_5505403) associated with the fourth and fifth year of growth in *Populus*.

**Figure S19** Interspecific expression proﬁles for the five potential time-specific genes in the genus *Populus*.

**Table S1** Descriptive statistic for each trait at each single timepoint in the linkage population and association mapping panel

**Table S2** Quantitative trait locus detection and segmental homology region identiﬁcation for three growth traits at 12 timepoints in a Populus interspeciﬁc linkage population

**Table S3** Annotation of dynamic QTL and corresponding segmental homology regions for three growth traits over 12 timepoints in Populus

**Table S4** Annotation and diversity assessment of genes and SNPs within 27 segmental homology regions in the association mapping panel of *Populus*

**Table S5** Summary of potential selective signals and genes within the SHRs in the association mapping panel.

**Table S6** Functional annotations of these selective protein-coding genes among all pairwise climatic regions of *Populus* (NW vs. S, NE vs. S or NW vs. NE).

**Table S7** Summary of the number of all significant SNP within SHRs associated with three growth traits over nine time points of *Populus*

**Table S8** Summary of pairwise SNP and gene epistasis for three traits at nine time points in association mapping panel of *Populus.*

**Table S9** The mean value for each trait at the nine time point in the three climatic regions of the association mapping panel

**Table S10** Primers used for reverse transcription quantitative PCR for the candidate genes

**Method S1** Identification and target prediction of noncoding RNA genes

**Method S2** Library construction for genome re-sequencing

**Method S3** Statistics formulas for Fst, θ_w_, π and Tajima’s D calculations

**Method S4** Total RNA extraction, cDNA synthesis, and reverse transcription quantitative PCR **Data S1-S14** were provided in other formats (Excel), which were submitted as a separate files

**Figure S1. Growth trajectories of tree height, basal diameter, and stem volume in *Populus* linkage population and association mapping panel.** By plotting height (H), basal diameter (BD), and stem volume (V) at continuous growth timepoints of *Populus* in a linkage population (1,200 individuals, 12 timepoints, **a–c**) and an association mapping panel (435 individuals, nine timepoints, **d–f**). In the linkage population, the red line is the ﬁt of the growth curves of the progeny of the cross between *Populus* *alba* × *P.* *glandulosa* (P1) as the female parent and *P. tomentosa* (P2) as the male parent, whereas the blue line is the observed data for the parents.

**
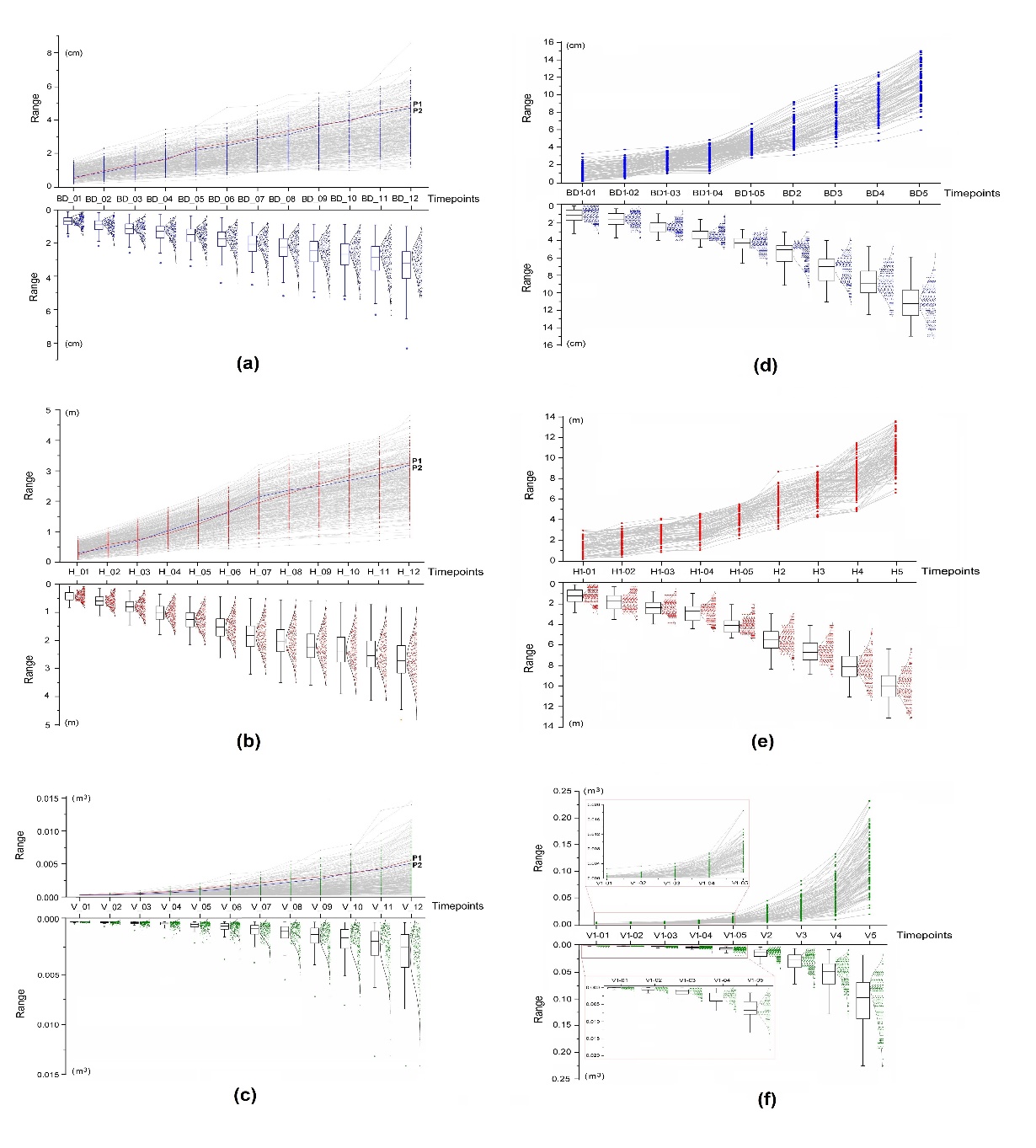
**

**Figure S2 The dynamic curves of the stem height to diameter ratio in the *Populus* linkage population and association mapping panel.** (**a**) In the linkage population, the increased height to diameter (H/D) ratio of the progeny (F1, *n* = 1,200) at the 12 timepoints is roughly consistent with the growth tendency of the two parents (P1 and P2). (b) In the association mapping panel, the H/D ratios decreased rapidly and then smooth gradually for the three climatic regions (NW, NE, and S) and for the total population during the growing periods.

**
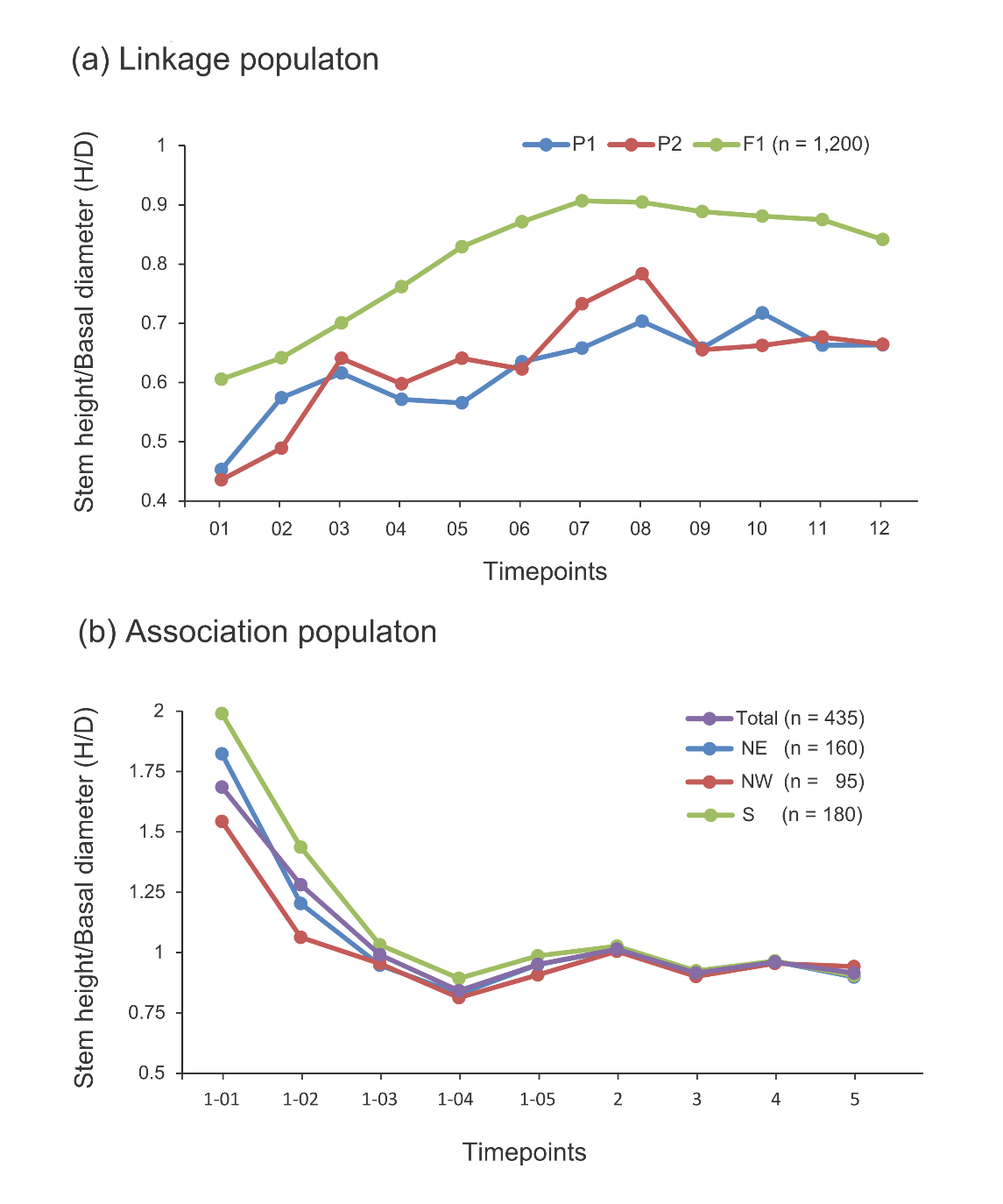
**

**Figure S3 Annotation and target gene prediction within all 27 putative SHRs** (**a**) Annotation of protein-coding genes, long noncoding RNA (lncRNA) genes, and microRNA (pre-miRNA) precursor genes within all sequences of the 27 SHRs. (**b**) Prediction of potential miRNA target genes and lncRNA target genes within all 27 SHRs.

**
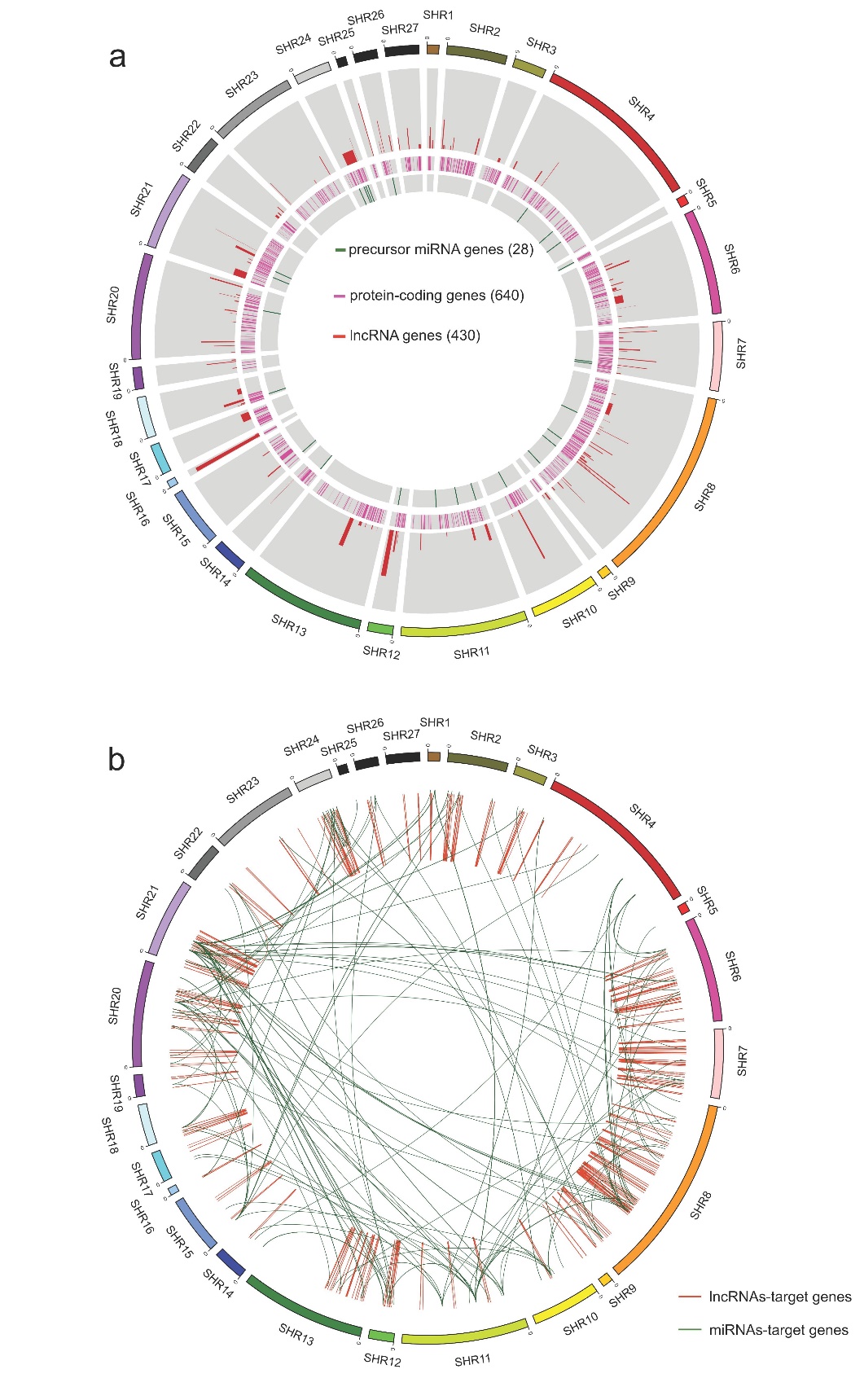
**

**Figure S4. Estimates of the squared allele-frequency correlations for all pairwise SNP combinations within each SHR.** *r*^2^, squared allele frequency correlation coefficient.

**
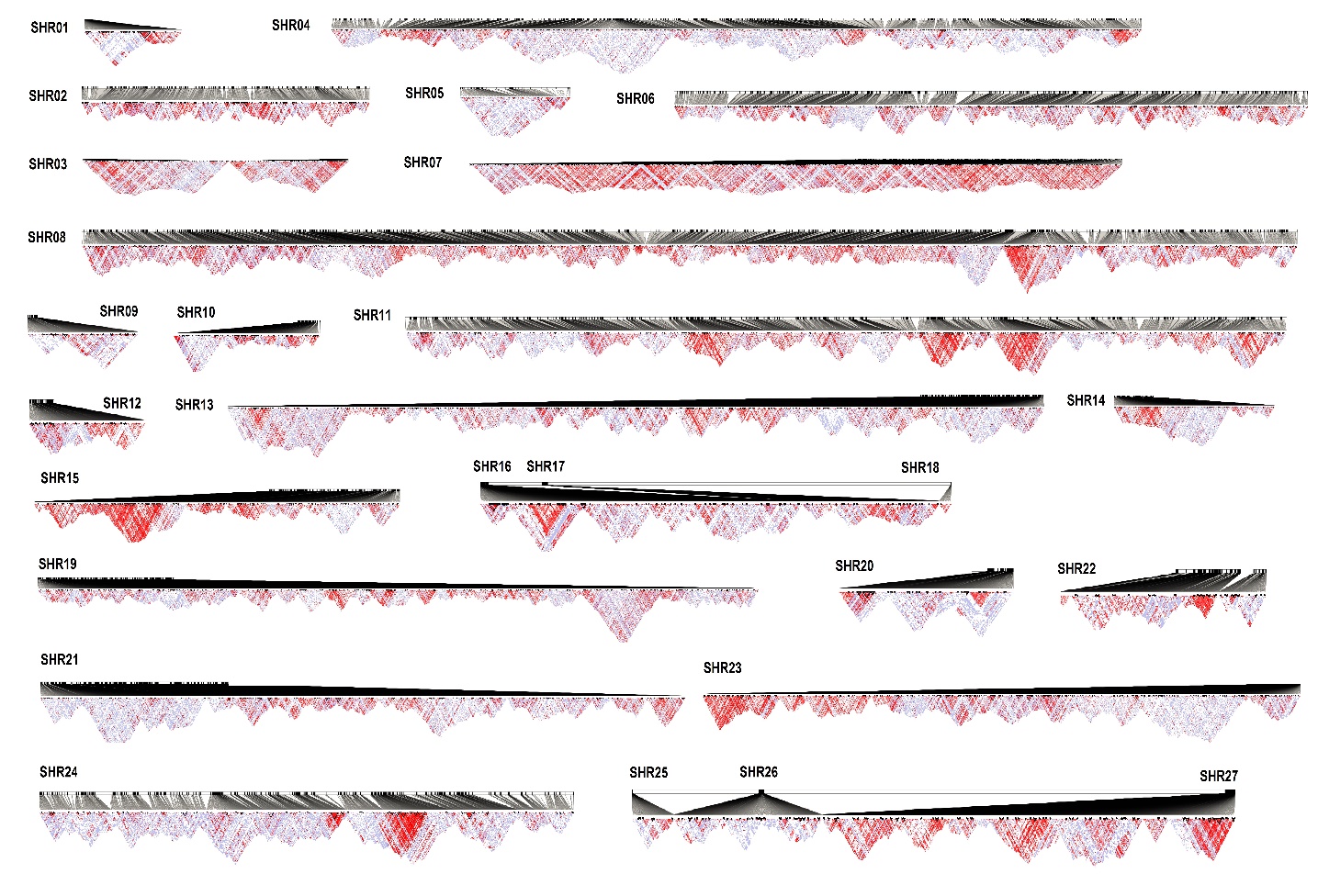
**

**Figure S5. The diverse patterns of linkage disequilibrium decay with physical distance between the common SNPs separately for each SHR.** The curves describe the nonlinear regressions of *r*^2^ onto the physical distance in base pairs (bp).

**
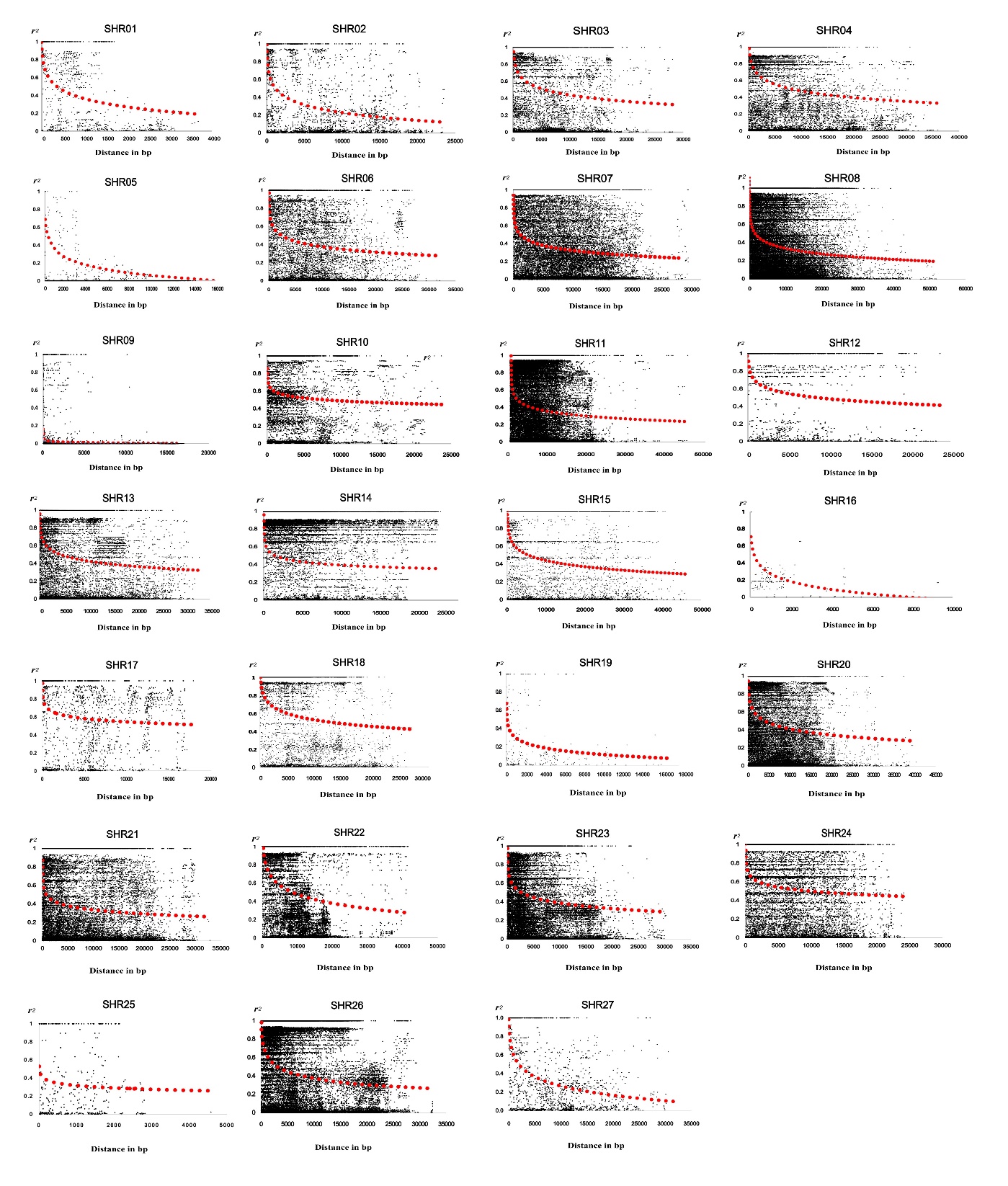
**

**Figure S6. Manhattan and quantile-quantile plots resulting from the association results between all SNPs of 27 SHRs and basal diameter at the nine timepoints.** The x-axis shows the SHR positions and the y-axis shows the significance expressed as −log_10_ *P* value. The dashed horizontal line depicts the Bonferroni-adjusted significance threshold (9.4 × 10^−7^). BD, basal diameter.

**
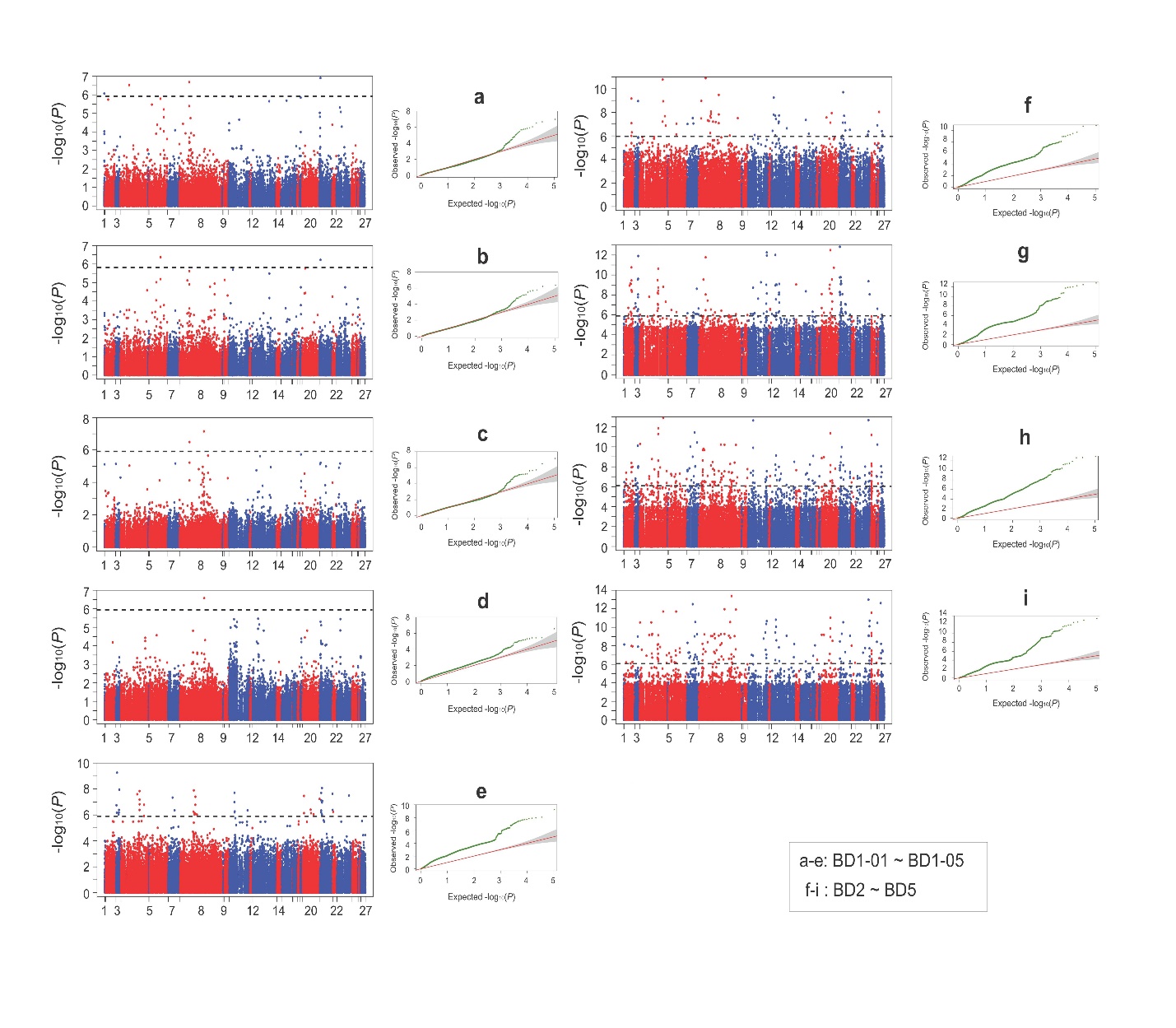
**

**Figure S7. Manhattan and quantile-quantile plots resulting from the association results between all SNPs of 27 SHRs and stem height at the nine timepoints.** The x-axis shows the SHR positions and the y-axis shows the significance expressed as −log_10_ *P* value. The dashed horizontal line depicts the Bonferroni-adjusted significance threshold (9.4 × 10^−7^). H, stem height.

**
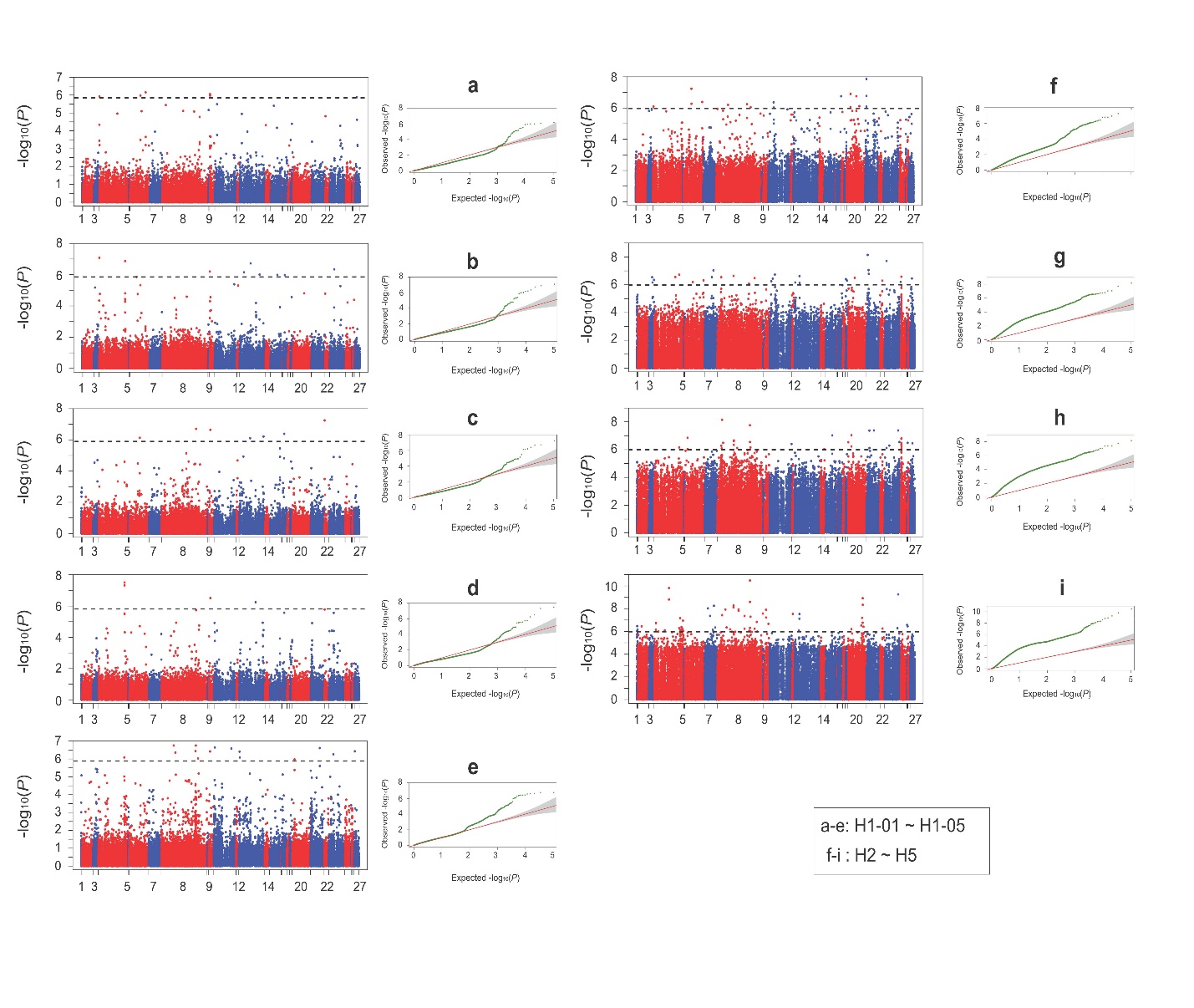
**

**Figure S8. Manhattan and quantile-quantile plots resulting from the association results between all SNPs of 27 SHRs and stem volume at the nine timepoints.** The x-axis shows the SHR positions and the y-axis shows the significance expressed as −log_10_ *P* value. The dashed horizontal line depicts the Bonferroni-adjusted significance threshold (9.4 × 10^−7^). V, stem volume.

**
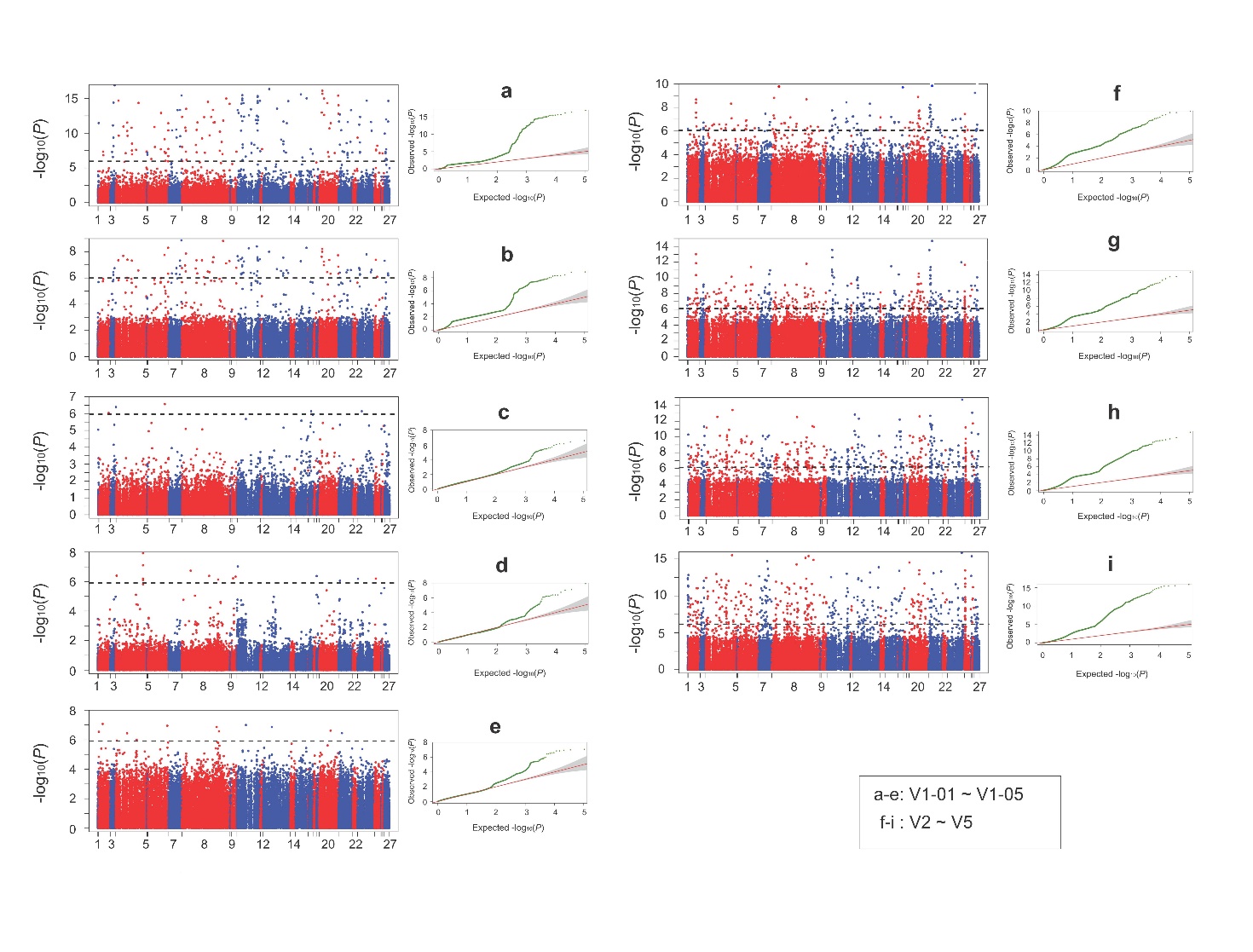
**

**Figure S9. The significance of 27 SHR-wide scans for basal diameter, tree height, and stem volume at the nine growth timepoints of *Populus***. Each row represents a trait and each column corresponds to a SHR containing multiple SNPs that are signiﬁcantly associated with a trait. Signiﬁcance is color-coded based on the *P* value of the association. BD, basal diameter; H, tree height; and V, stem volume.

**
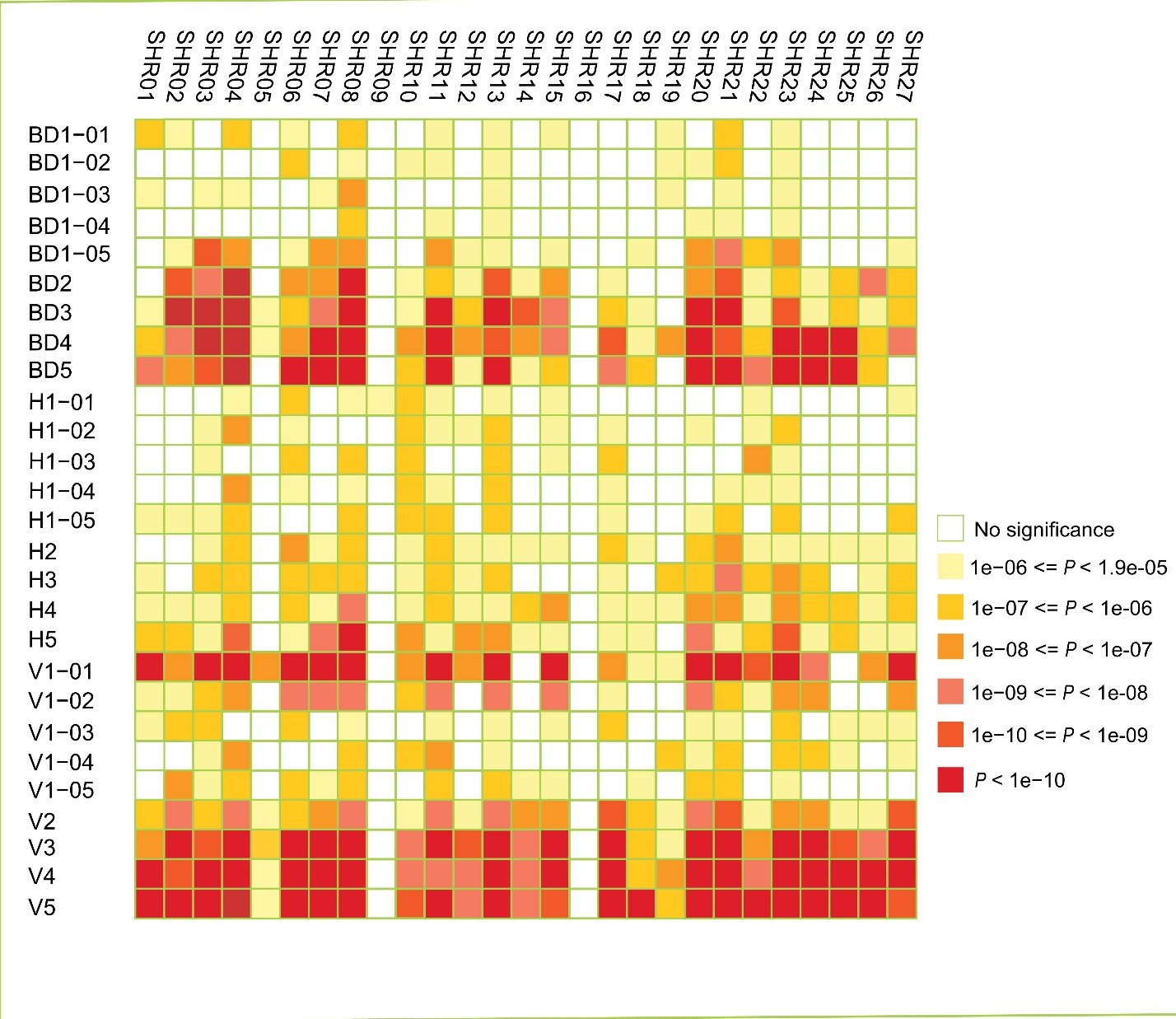
**

**Figure S10. Structural networks that represent signiﬁcant gene, lncRNA, and miRNA loci for basal diameter, tree height, and stem volume at the nine growth timepoints of *Populus*.** Nodes depict genes and edges depict signiﬁcant associations at *P* < 1.9×10^-5^. BD, basal diameter; H, tree height; and V, stem volume.

**
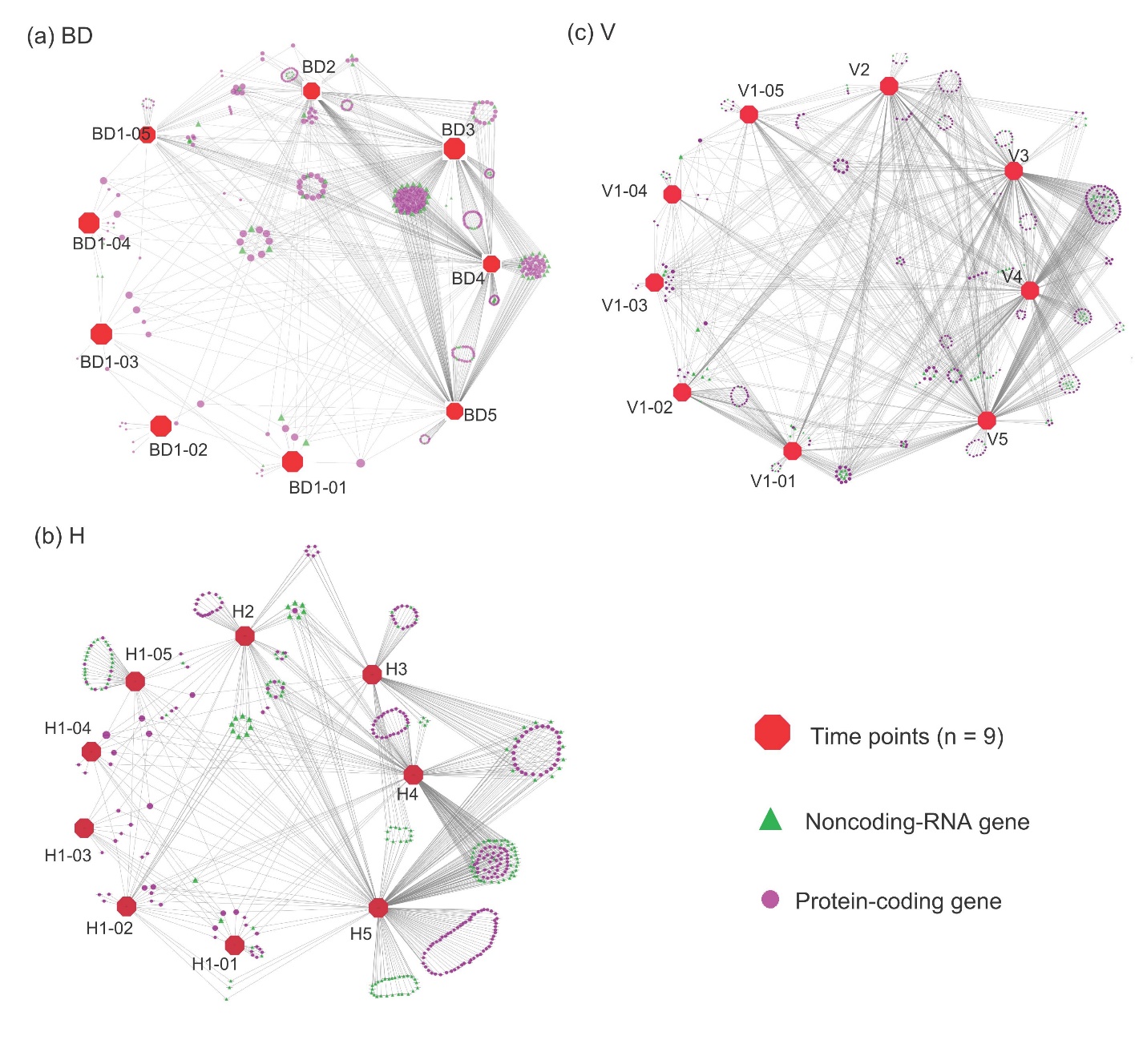
**

**Figure S11. Identification of the association hotspots related to the protein-coding genes and noncoding RNA genes at the suggestive and significant *P*-values, respectively.** * represent the association hotspot (repeatedly associated with at least 12 data sets, α < 0.05) Protein-coding genes (a, b) and noncoding RNA genes (c, d).

**
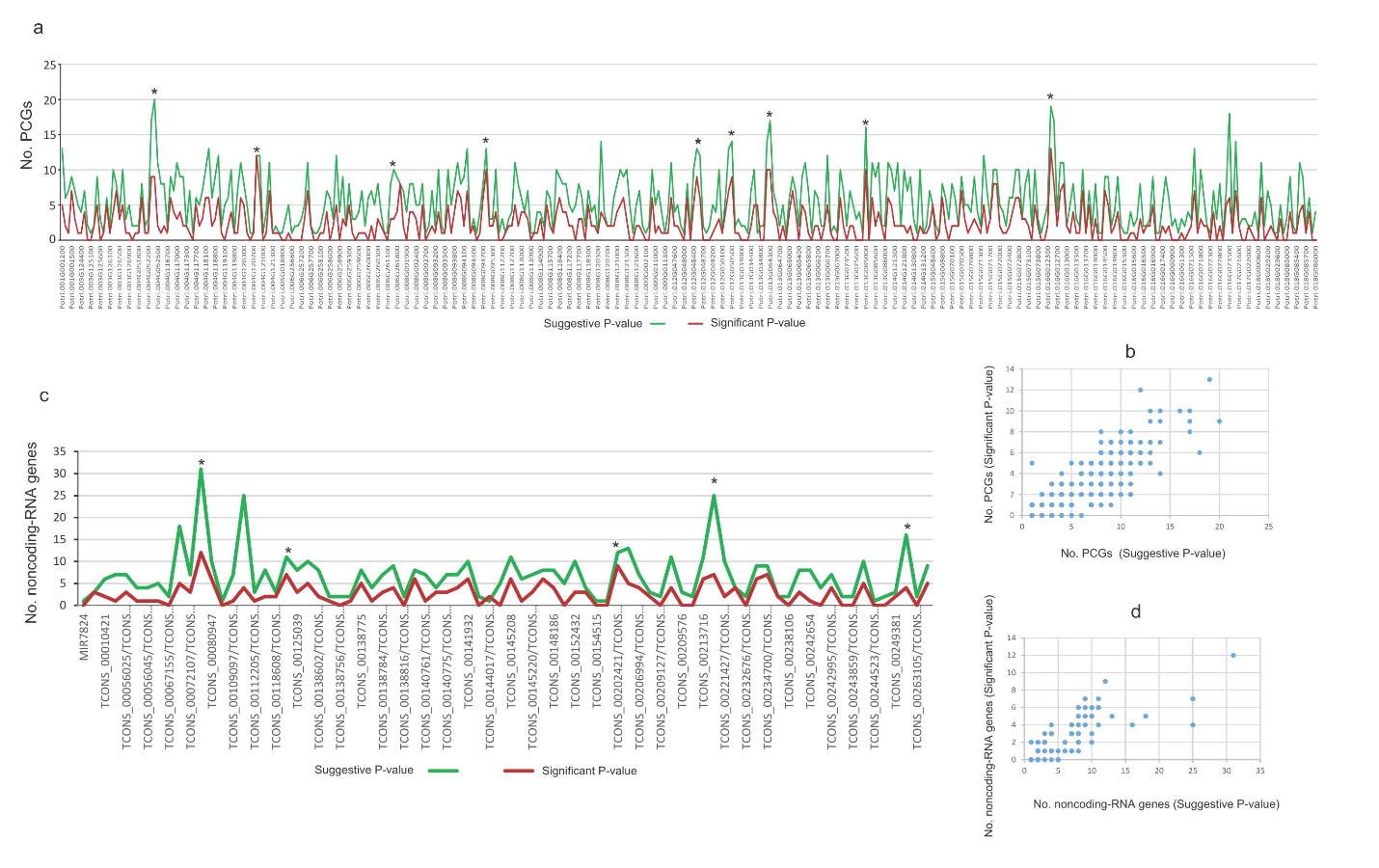
**

**Figure S12. The genetic effect and phenotypic contributions of Chr04_11343447 and Chr16_5505403 over the two- to five-year growth phases.** (a) A pleiotropic SNP, Chr04_11343447, had significant additive effects for H and V, but a significant dominant effect for BD from the two- to five-year growth phases. (b) Chr16_5505403 contributed an exclusively dominant effect for all three traits over the two- to five-year growth phases. The phenotypic contributions of Chr04_11343447 and Chr16_5505403 increased gradually over the two- to five-year growth phases for the three traits.

**
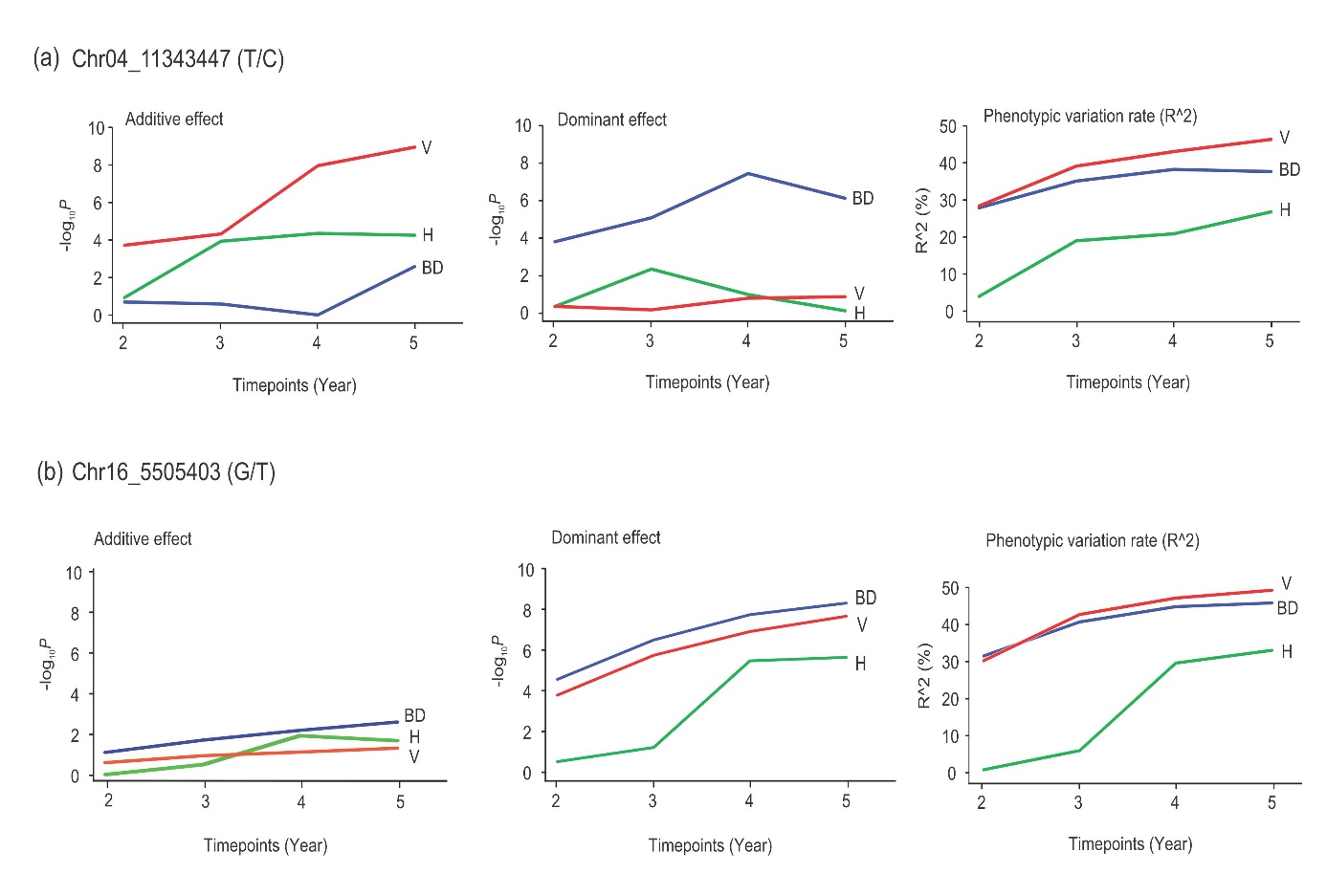
**

**Figure S13. Gene–gene interactions formed uniquely interconnected networks for basal diameter at the nine timepoints.** Significant gene–gene interactions are connected by red lines. Different colored segments of the circle show all 27 SHRs of the *Populus* reference genome. BD, basal diameter

**
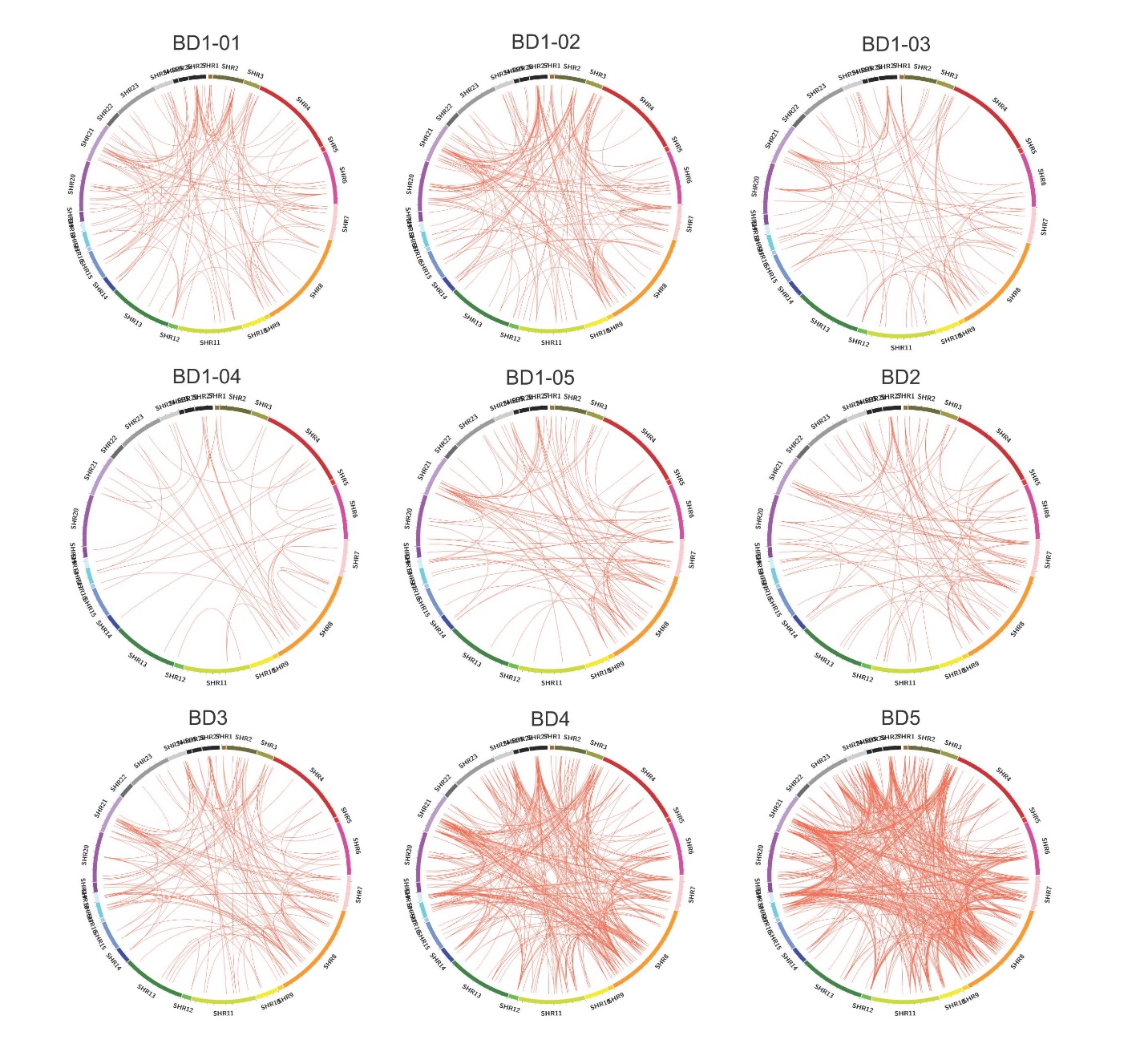
**

**Figure S14. Gene–gene interactions formed uniquely interconnected networks for tree height at the nine timepoints.** Significant gene-gene interactions are connected by blue lines. Different colored segments of the circle show all 27 SHRs of the *Populus* reference genome. H, tree height.

**
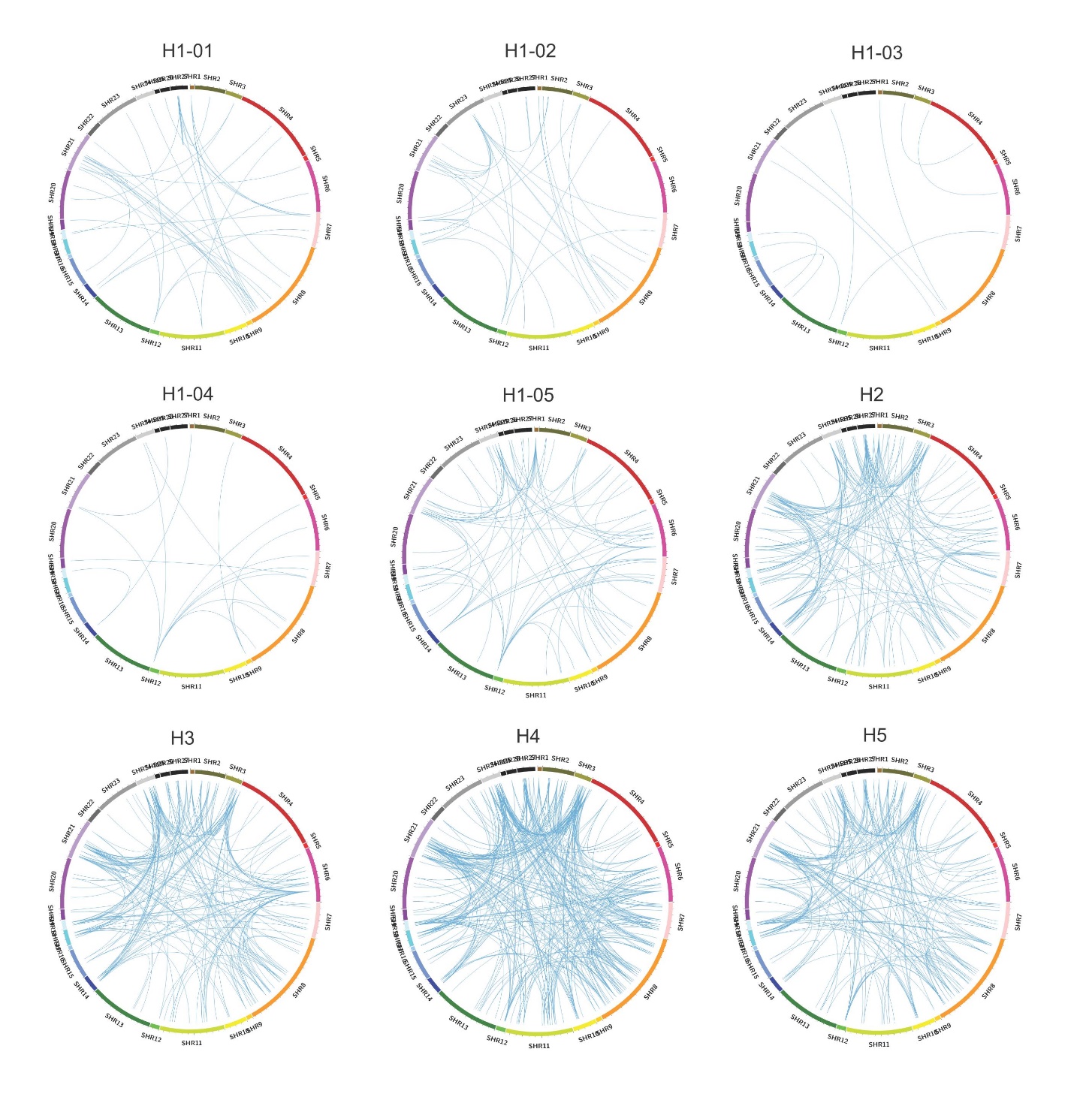
**

**Figure S15. Gene–gene interactions formed uniquely interconnected networks for stem volume at the nine timepoints.** Significant gene–gene interactions are connected by green lines. Different colored segments of the circle show all 27 SHRs of the *Populus* reference genome.

**
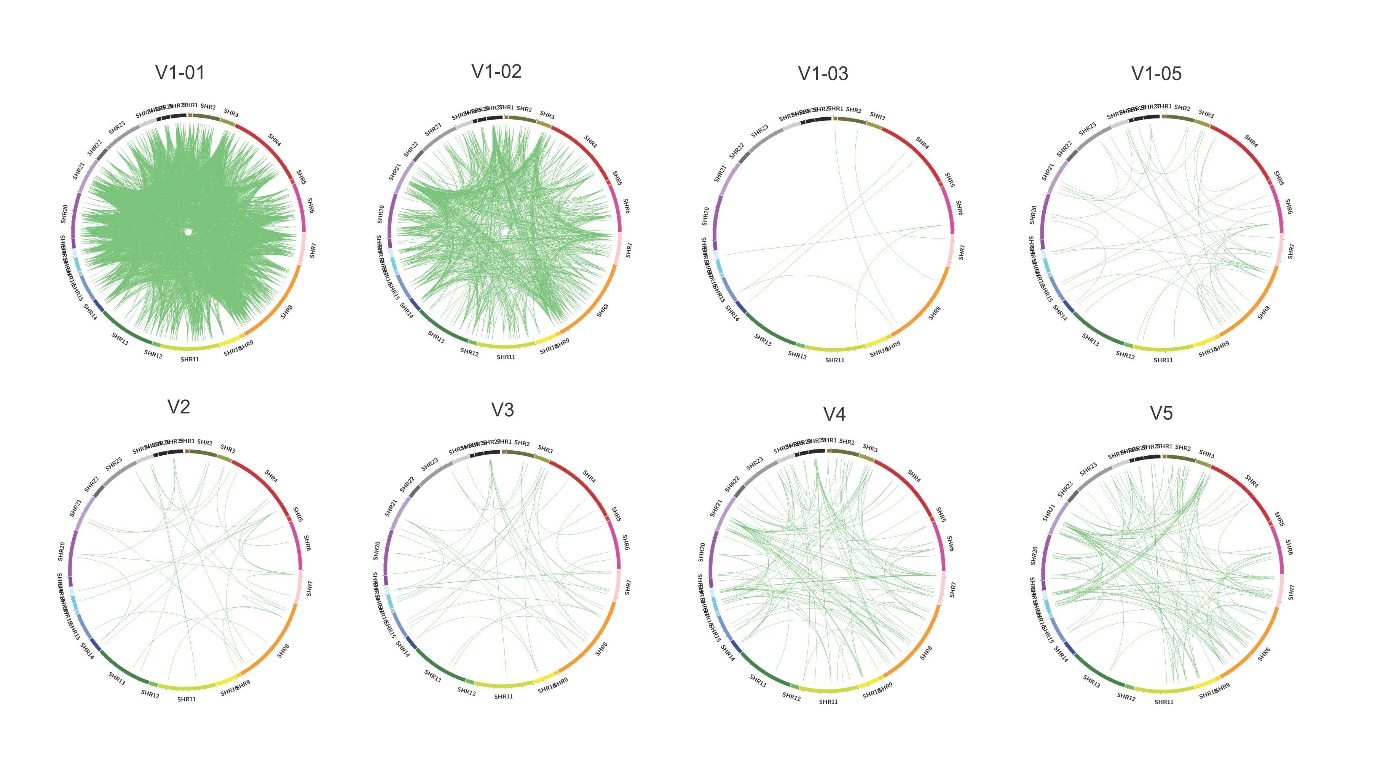
**

**Figure 16. The SHR08 locus contains causative signals underlying growth-stage-specific stem height and basal diameter growth.** (**a**) Manhattan plots displaying the association results between all SNP positions of 27 SHRs (x-axis) and seven time-specific height (H) and basal diameter (BD) traits. The y-axis shows the significance expressed as −log_10_ *P* value. (**b**) The lead SNP (the SNP with the lowest *P* value) for each time-specific H and BD trait, whose position is indicated in the SHR08 locus by a larger black dot. The dashed horizontal line depicts the Bonferroni-adjusted significance threshold (9.4 × 10^−7^). (**c**) The selection signals within SHR08 between the northwestern (NW) and southern (S) climatic regions of *Populus tomentosa*. Six candidate genes are shown at the bottom (red rectangle, coding sequences; black line, introns; green rectangle, 5’ and 3’ untranslated regions). The stars represent the signiﬁcant selective signals. (**d**) Box plot for each growth trait (orange) and expression of each linked gene (sky blue) is plotted as a genetic effect of genotypes at the lead SNP. The horizontal line represents the mean and the vertical lines mark the range from the 5th and 95th percentile of the total data. (**e**) A linkage disequilibrium (LD) representation of the pair-wise *r*^2^ value among all polymorphic sites (SNPs and InDels) across the three adjacent genes, where a deeper red colour corresponds to a higher *r*^2^ value. (**f**) Genotypic effect of the significant InDel locus on BD5 and the expression of *Potri.008G121200*. Plot of correlation between BD5 and the expression level of *Potri.008G121200* among different genotypes of the InDel site. The *r* value is based on a Pearson correlation coefﬁcient. The *P* value is calculated using the t approximation. (**g**) A representation of the pair-wise *r*^2^ value among all polymorphic sites (SNPs and InDels) across the adjacent *Potri.008G113300* and *Potri.008G114200* genes. (**h**) Genotypic effect of the significant InDel locus on H4 (red) and *Potri.008G113300* expression (sky blue). Plot of the correlation between H4 and the expression level of *Potri.008G113300* among the genotype classes.


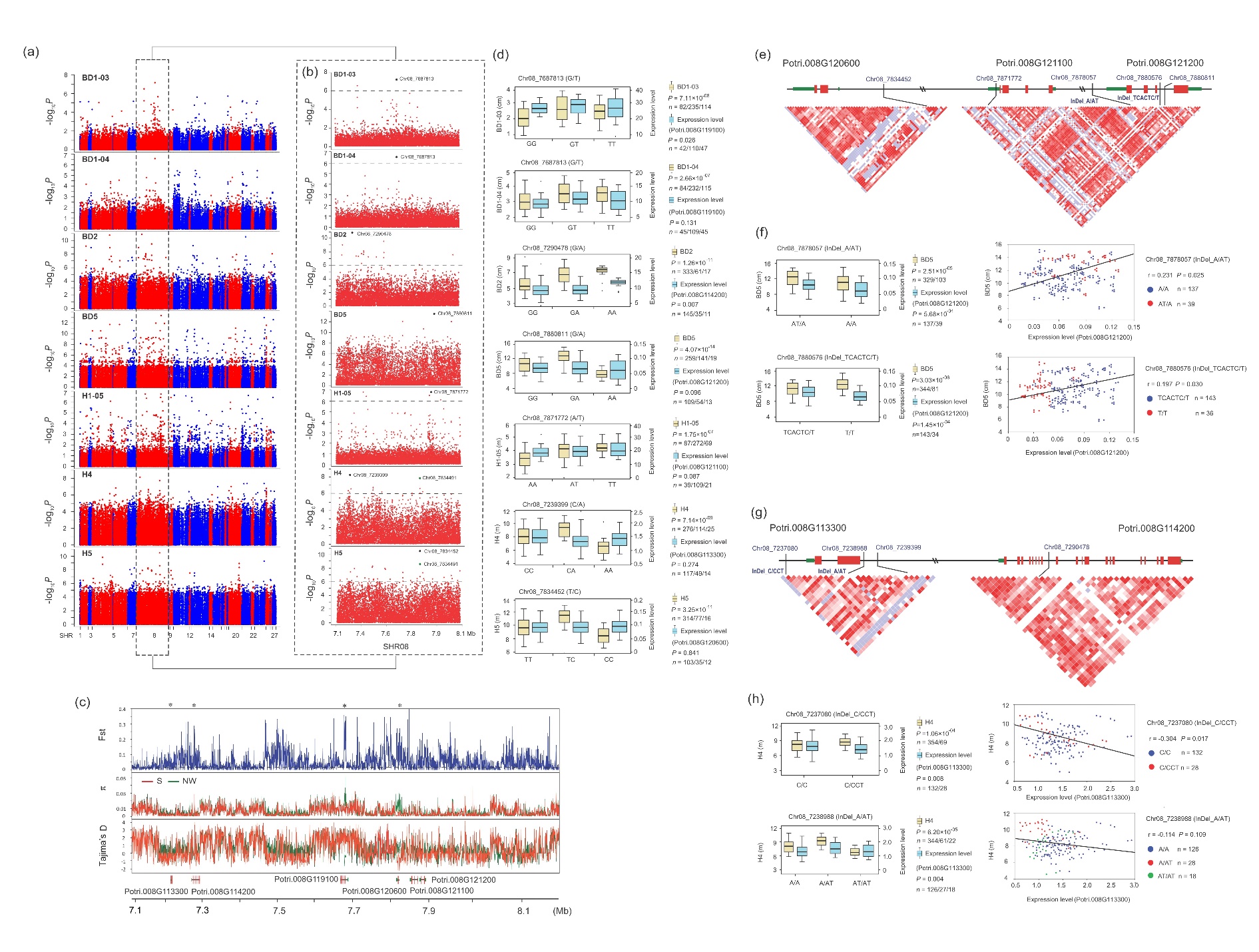


**Figure S17 The allelic distributions of 12 lead SNP sites among individuals from the three climatic regions.** The detailed information of these lead SNP and genes were shown in Table 1; Southern (S), Northwestern (NW), and Northeastern (NE) climatic regions. Red shows the frequency of the major allele and blue shows the frequency of the minor allele.

**
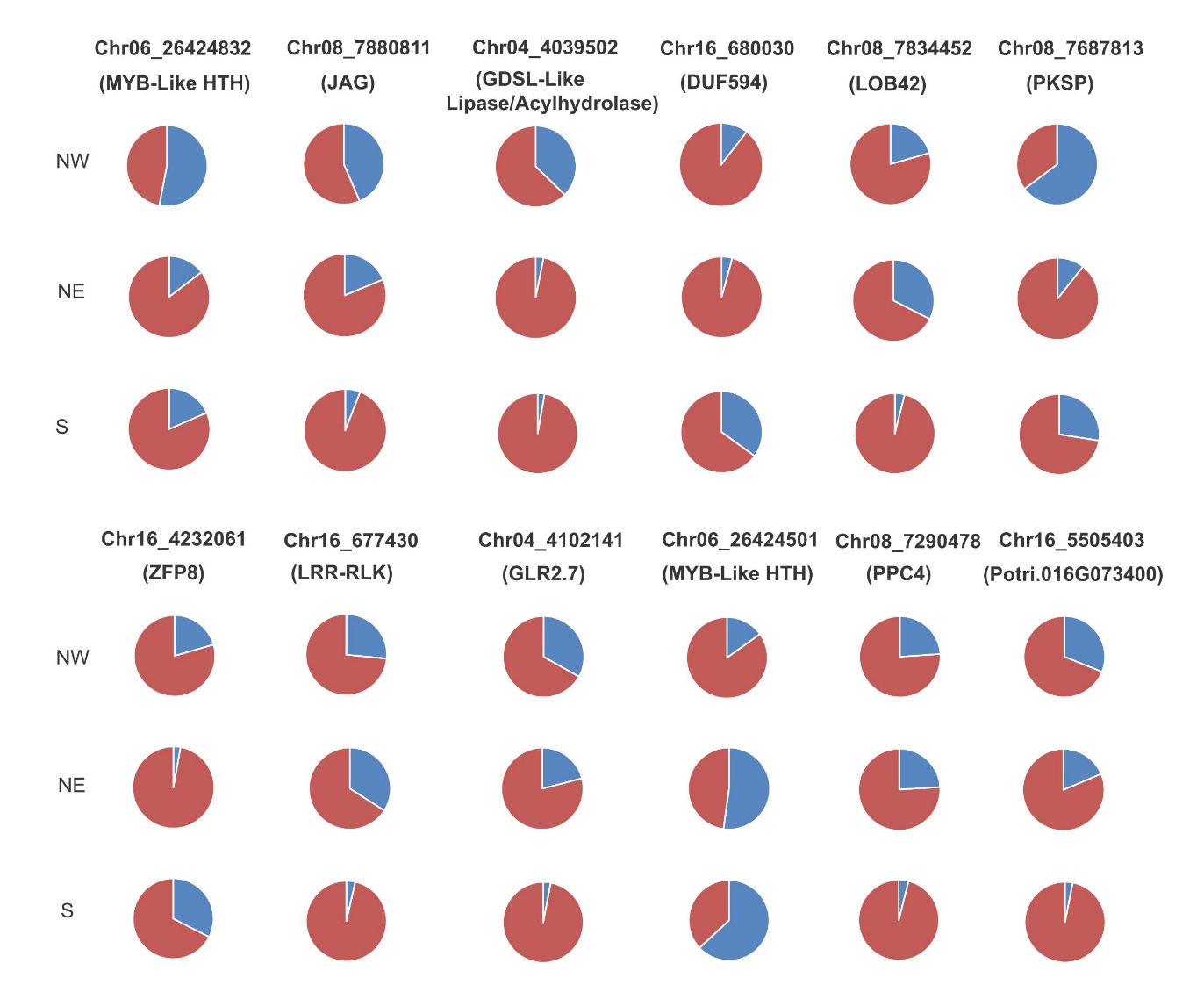
**

**Figure 18. SHR23 may contain a causal SNP (Chr16_5505403) associated with the fourth and fifth year of growth in *Populus.*** (a) Manhattan plots displaying the association results between all SNPs of 27 SHRs and the three growth traits in the fourth and fifth years of growth. The x-axis shows the SHR positions and the y-axis shows the significance expressed as −log_10_ *P* value. (b) The top-ranked SNP (Chr16_5505403) for each trait at the fourth and fifth years of growth, whose position is indicated in the SHR23 locus by a larger black dot. The dashed horizontal line depicts the Bonferroni-adjusted significance threshold (9.4 × 10^−7^). Two linked candidate genes are shown at the bottom (red rectangle, coding sequences; black line, introns; green rectangle, 5’ and 3’ untranslated regions). (c) Box plot for the growth trait (red and sky blue) is plotted as an effect of genotypes at the SNP Chr16_5505403. The horizontal line represents the mean and the vertical lines mark the range from the 5th and 95th percentile of the total data. Plots of correlation between each trait pair among the genotype classes. The *r* value is based on a Pearson correlation coefﬁcient. The *P* value is calculated using the *t* approximation. (d) Box plot for the expression values of *Potri.016G073400* (red) and *Potri.016G073500* (sky blue) is plotted as an effect of genotypes at SNP Chr16_5505403. Plots of correlation between expression levels of both genes among the genotype classes.

**
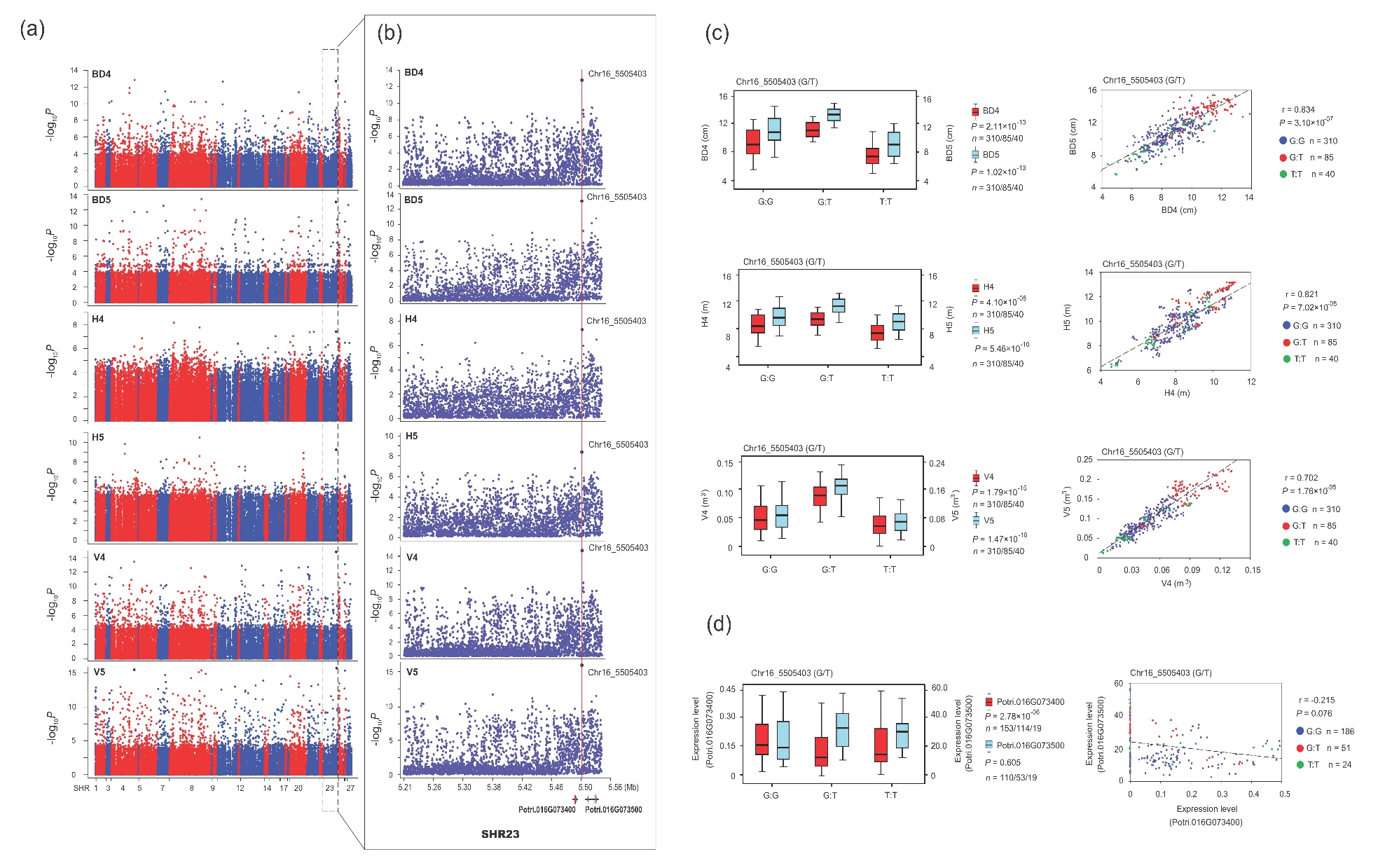
**

**Figure S19 Interspecific expression proﬁles for the five potential time-specific genes in the genus *Populus*.** (**a)** Phylogenetic analysis of five *WRKY3* orthologs of *Populus* *spp*. (*Salix purpurea* as the out-group) using the orthologous proteins in MEAG 6.0. *WRKY3* exhibits distinct expression profiles within the vascular cambium and shoot apical meristem (SAM) at the nine growth timepoints among five *Populus* species, where the color of each box corresponds to the log_2_ fold change value of expression according to the legend. The heatmap was generated from hierarchical cluster analysis of genes. (**b**) Eleven *Potri.16G073400* orthologs (using BLASP in NCBI) were divided into three major classes of the phylogenetic tree (monocots, annual herbaceous dicots, and perennial dicots) with well-supported bootstrap values in MEAG 6.0. *Potri.16G073400* orthologs exhibit distinct expression profiles within the vascular cambium and SAM at the nine growth timepoints among five *Populus* species. The same phylogenetic cluster and time-specific expression profiles were observed for (**c)** *Potri.004G120900* (*DUF3598*), (**d**) *Potri.016G012400* (*DUF594*) and (**e**) *Potri.016G013200* (unknown gene) within the vascular cambium and SAM among five *Populus* species.

**
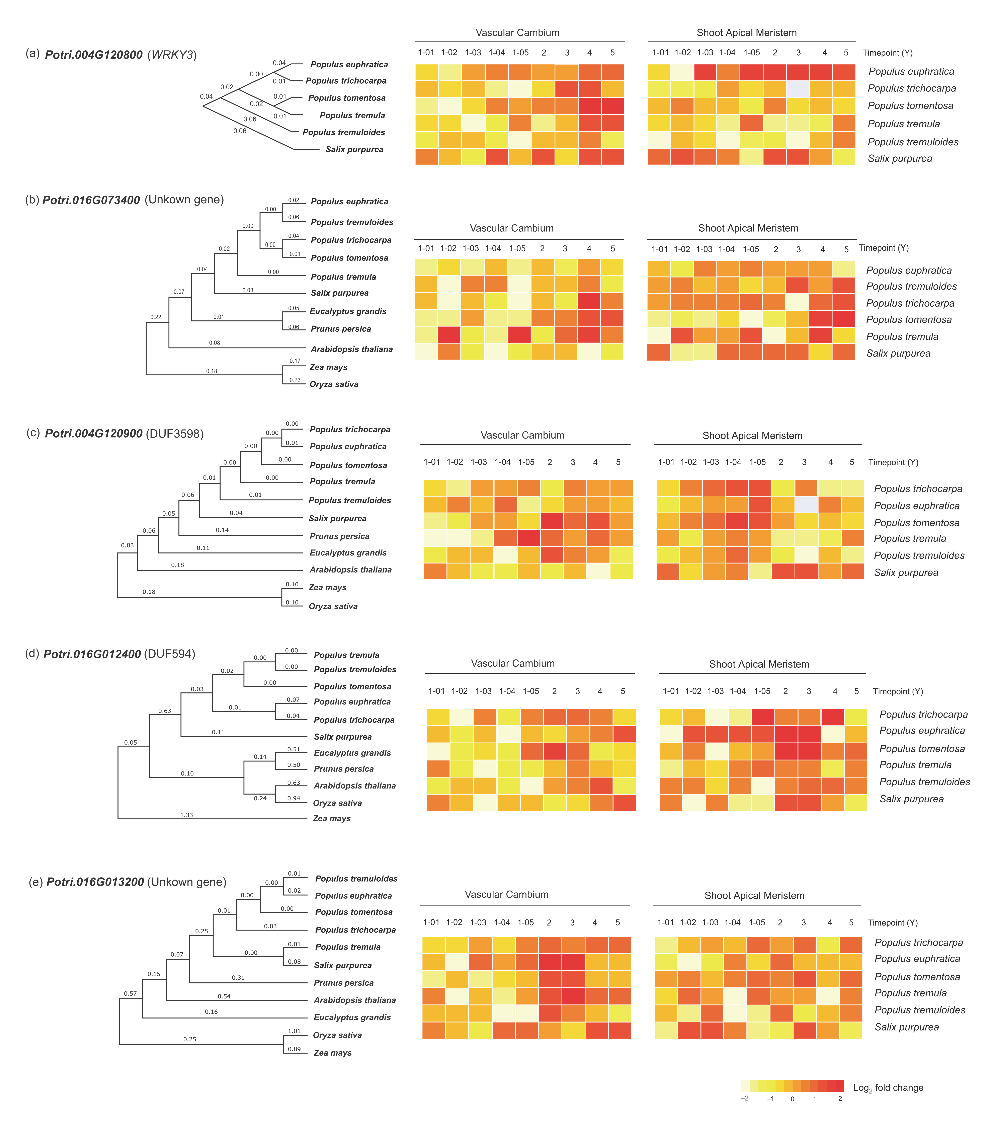
**

**Table S1** Descriptive statistic for each trait at the each time point in the linkage population and association mapping panel, respectively

| **Population** | **Trait** | **Mean** | **Standard Deviation** | **Minimum** | **Maximum** | **Standard Error** | **N** |
| --- | --- | --- | --- | --- | --- | --- | --- |
| **Linkage**  **population** | H_01 | 0.4273 | 0.1639 | 0.0720 | 0.8270 | 0.0047 | 1200 |
|  | H_02 | 0.5998 | 0.1993 | 0.1500 | 1.1230 | 0.0058 | 1200 |
|  | H_03 | 0.8012 | 0.2564 | 0.2240 | 1.4520 | 0.0074 | 1200 |
|  | H_04 | 1.0234 | 0.3146 | 0.3510 | 1.7950 | 0.0091 | 1200 |
|  | H_05 | 1.2682 | 0.3654 | 0.4500 | 2.1500 | 0.0105 | 1200 |
|  | H_06 | 1.5187 | 0.4290 | 0.4420 | 2.6220 | 0.0124 | 1200 |
|  | H_07 | 1.8447 | 0.5143 | 0.5000 | 3.2040 | 0.0148 | 1200 |
|  | H_08 | 2.0247 | 0.5596 | 0.5560 | 3.5060 | 0.0162 | 1200 |
|  | H_09 | 2.1926 | 0.5894 | 0.5820 | 3.6010 | 0.0170 | 1200 |
|  | H_10 | 2.3424 | 0.6174 | 0.6420 | 3.9080 | 0.0178 | 1200 |
|  | H_11 | 2.5043 | 0.6451 | 0.7230 | 4.1300 | 0.0186 | 1200 |
|  | H_12 | 2.6808 | 0.6963 | 0.8210 | 4.8300 | 0.0201 | 1200 |
|  | BD_01 | 0.7216 | 0.2797 | 0.1600 | 1.5860 | 0.0081 | 1200 |
|  | BD_02 | 0.9709 | 0.3612 | 0.2080 | 2.1900 | 0.0104 | 1200 |
|  | BD_03 | 1.1844 | 0.4171 | 0.2830 | 2.6250 | 0.0120 | 1200 |
|  | BD_04 | 1.3934 | 0.4918 | 0.3210 | 3.2170 | 0.0142 | 1200 |
|  | BD_05 | 1.5922 | 0.5570 | 0.3940 | 3.4100 | 0.0161 | 1200 |
|  | BD_06 | 1.8170 | 0.6109 | 0.4980 | 4.4400 | 0.0176 | 1200 |
|  | BD_07 | 2.1198 | 0.7014 | 0.8150 | 4.5460 | 0.0202 | 1200 |
|  | BD_08 | 2.3356 | 0.7842 | 0.8790 | 5.1520 | 0.0226 | 1200 |
|  | BD_09 | 2.5761 | 0.8690 | 0.8940 | 5.2580 | 0.0251 | 1200 |
|  | BD_10 | 2.7794 | 0.9352 | 0.9060 | 5.3530 | 0.0270 | 1200 |
|  | BD_11 | 3.0107 | 1.0415 | 0.9340 | 6.3320 | 0.0301 | 1200 |
|  | BD_12 | 3.3661 | 1.1659 | 1.0270 | 8.4100 | 0.0337 | 1200 |
|  | V_01 | 2.46E-05 | 2.41E-05 | 1.48E-07 | 1.41E-04 | 6.96E-07 | 1200 |
|  | V_02 | 5.98E-05 | 5.85E-05 | 1.15E-06 | 4.03E-04 | 1.69E-06 | 1200 |
|  | V_03 | 1.16E-04 | 1.09E-04 | 3.66E-06 | 7.33E-04 | 3.14E-06 | 1200 |
|  | V_04 | 2.04E-04 | 1.96E-04 | 6.69E-06 | 1.37E-03 | 5.66E-06 | 1200 |
|  | V_05 | 3.24E-04 | 3.03E-04 | 1.05E-05 | 1.83E-03 | 8.74E-06 | 1200 |
|  | V_06 | 4.93E-04 | 4.33E-04 | 1.79E-05 | 3.23E-03 | 1.25E-05 | 1200 |
|  | V_07 | 8.06E-04 | 6.75E-04 | 3.30E-05 | 3.54E-03 | 1.95E-05 | 1200 |
|  | V_08 | 1.08E-03 | 9.21E-04 | 3.89E-05 | 5.17E-03 | 2.66E-05 | 1200 |
|  | V_09 | 1.43E-03 | 1.22E-03 | 4.61E-05 | 6.84E-03 | 3.52E-05 | 1200 |
|  | V_10 | 1.77E-03 | 1.49E-03 | 5.44E-05 | 7.73E-03 | 4.3E-05 | 1200 |
|  | V_11 | 2.24E-03 | 1.96E-03 | 6.46E-05 | 1.28E-02 | 5.66E-05 | 1200 |
|  | V_12 | 2.98E-03 | 2.72E-03 | 7.91E-05 | 2.45E-02 | 7.85E-05 | 1200 |
|  |  |  |  |  |  |  |  |
| **Association**  **mapping panel** | H_1-01 | 1.2380 | 0.6654 | 0.1189 | 2.8411 | 0.0319 | 435 |
|  | H_1-02 | 1.7684 | 0.7490 | 0.3268 | 3.5118 | 0.0359 | 435 |
|  | H_1-03 | 2.3736 | 0.7179 | 0.7751 | 3.9621 | 0.0344 | 435 |
|  | H_1-04 | 2.8745 | 0.8691 | 0.9770 | 4.4196 | 0.0417 | 435 |
|  | H_1-05 | 4.0802 | 0.7517 | 2.0568 | 5.3115 | 0.0360 | 435 |
|  | H_2 | 5.4534 | 1.0807 | 2.9587 | 8.3710 | 0.0518 | 435 |
|  | H_3 | 6.5735 | 1.0633 | 4.0794 | 8.8736 | 0.0510 | 435 |
|  | H_4 | 8.1843 | 1.4147 | 4.6200 | 11.0604 | 0.0678 | 435 |
|  | H_5 | 10.0387 | 1.4815 | 6.3749 | 13.1145 | 0.0710 | 435 |
|  | BD_1-01 | 1.1464 | 0.7254 | 0.0175 | 3.2075 | 0.0348 | 435 |
|  | BD_1-02 | 1.6117 | 0.7230 | 0.3559 | 3.6909 | 0.0347 | 435 |
|  | BD_1-03 | 2.4799 | 0.6597 | 0.9350 | 3.9797 | 0.0316 | 435 |
|  | BD_1-04 | 3.3311 | 0.7059 | 0.9686 | 4.7833 | 0.0338 | 435 |
|  | BD_1-05 | 4.3407 | 0.7996 | 2.7199 | 6.6274 | 0.0383 | 435 |
|  | BD_2 | 5.5365 | 1.3612 | 3.0497 | 9.1456 | 0.0653 | 435 |
|  | BD_3 | 7.3050 | 1.5153 | 3.9990 | 11.0313 | 0.0727 | 435 |
|  | BD_4 | 8.8348 | 1.6759 | 4.6940 | 12.5152 | 0.0804 | 435 |
|  | BD_5 | 11.1795 | 1.9776 | 5.8980 | 14.9750 | 0.0948 | 435 |
|  | V_1-01 | 2.08E-04 | 3.17E-04 | 8.31E-09 | 2.38E-03 | 1.52E-05 | 435 |
|  | V_1-02 | 4.87E-04 | 5.09E-04 | 1.10E-05 | 3.19E-03 | 2.44E-05 | 435 |
|  | V_1-03 | 1.32E-03 | 8.82E-04 | 9.16E-05 | 3.96E-03 | 4.23E-05 | 435 |
|  | V_1-04 | 2.78E-03 | 1.62E-03 | 1.42E-04 | 6.80E-03 | 7.77E-05 | 435 |
|  | V_1-05 | 6.44E-03 | 3.01E-03 | 1.57E-03 | 1.83E-02 | 1.44E-04 | 435 |
|  | V_2 | 1.45E-02 | 8.82E-03 | 2.65E-03 | 4.23E-02 | 4.23E-04 | 435 |
|  | V_3 | 3.00E-02 | 1.55E-02 | 5.12E-03 | 7.86E-02 | 7.42E-04 | 435 |
|  | V_4 | 5.38E-02 | 2.63E-02 | 7.08E-03 | 1.28E-01 | 1.26E-03 | 435 |
|  | V_5 | 1.05E-01 | 4.69E-02 | 1.74E-02 | 2.26E-01 | 2.25E-03 | 435 |

H=Tree height, V=stem volume, BD=basal diameter

**Table S2** *Quantitative trait locus detection and segmental homology region identiﬁcation for three growth traits at 12 timepoints in a Populus interspeciﬁc linkage population*

| **Trait** | **Linkage Group** | **Range of QTL interval (cM)** | **Marker Position (cM)** | **Adjust QTL**  **interval (cM)** | **Peak marker^a^** | **LOD score^b^** | **Variation**  **explained (%) ^c^** | **Segmental homology region in Chromosome (bp)** | **No. of genes** |
| --- | --- | --- | --- | --- | --- | --- | --- | --- | --- |
| BD_11 | LG_I | 72.2-73.6 | 72.7 | 72.2-73.6 | Ptr_1_SSR2 | 3.5 | 0.101 | Chr01:96484-109863 | 4 |
| H_01 | LG_I | 72.2-73.6 | 72.7 | 72.2-73.6 | Ptr_1_SSR2 | 3.5 | 0.113 | Chr01:96484-109863 | 4 |
| H_02 | LG_I | 71.8-72.9 | 72.2 | 71.8-72.9 | Ptr_1_SSR2 | 3.4 | 0.039 | Chr01:96484-109863 | 4 |
| H_03 | LG_I | 72.2-73.6 | 72.7 | 72.2-73.6 | Ptr_1_SSR2 | 3.7 | 0.111 | Chr01:96484-109863 | 4 |
| V_01 | LG_I | 72.2-73.9 | 72.7 | 72.2-75.1 | Ptr_1_SSR2 | 3.4 | 0.148 | Chr01:96484-109863 | 4 |
| V_02 | LG_I | 72.2-74.1 | 72.7 | 72.2-75.1 | Ptr_1_SSR2 | 3.7 | 0.203 | Chr01:96484-109863 | 4 |
| V_03 | LG_I | 72.2-75.1 | 72.7 | 72.2-75.1 | Ptr_1_SSR2 | 3.7 | 0.217 | Chr01:96484-109863 | 4 |
| BD_01 | LG_I | 278.9-280.9 | 279.4 | 278.3-280.9 | Ptr_13_SSR60 | 3.2 | 0.040 | Chr13:7892575-8114972 | 16 |
| BD_02 | LG_I | 279.3-280.3 | 279.4 | 278.3-280.9 | Ptr_13_SSR60 | 5.2 | 0.096 | Chr13:7892575-8114972 | 16 |
| BD_03 | LG_I | 278.7-279.6 | 279.3 | 278.3-280.9 | Ptr_13_SSR60 | 5.1 | 0.109 | Chr13:7892575-8114972 | 16 |
| BD_04 | LG_I | 278.3-279.6 | 278.8 | 278.3-280.9 | Ptr_13_SSR60 | 4.2 | 0.143 | Chr13:7892575-8114972 | 16 |
| BD_05 | LG_I | 278.3-279.6 | 279.3 | 278.3-280.9 | Ptr_13_SSR60 | 3.3 | 0.069 | Chr13:7892575-8114972 | 16 |
| BD_06 | LG_I | 278.9-280.9 | 279.4 | 278.3-280.9 | Ptr_13_SSR60 | 3.3 | 0.064 | Chr13:7892575-8114972 | 16 |
| BD_10 | LG_I | 278.7-280.2 | 279.4 | 278.3-280.9 | Ptr_13_SSR60 | 3.5 | 0.045 | Chr13:7892575-8114972 | 16 |
| BD_11 | LG_I | 278.3-279.5 | 278.8 | 278.3-280.9 | Ptr_13_SSR60 | 3.5 | 0.163 | Chr13:7892575-8114972 | 16 |
| BD_12 | LG_I | 278.3-279.6 | 278.8 | 278.3-280.9 | Ptr_13_SSR60 | 3.4 | 0.143 | Chr13:7892575-8114972 | 16 |
| H_01 | LG_I | 279-280.9 | 279.4 | 279.0-280.9 | Ptr_13_SSR60 | 3.3 | 0.037 | Chr13:7892575-8114972 | 16 |
| H_02 | LG_I | 279-280.8 | 279.4 | 279.0-280.9 | Ptr_13_SSR60 | 3.1 | 0.030 | Chr13:7892575-8114972 | 16 |
| H_03 | LG_I | 279-280.8 | 279.4 | 279.0-280.9 | Ptr_13_SSR60 | 3.1 | 0.030 | Chr13:7892575-8114972 | 16 |
| H_04 | LG_I | 279-280.8 | 279.4 | 279.0-280.9 | Ptr_13_SSR60 | 3.4 | 0.060 | Chr13:7892575-8114972 | 16 |
| H_05 | LG_I | 279-280.8 | 279.4 | 279.0-280.9 | Ptr_13_SSR60 | 3.1 | 0.030 | Chr13:7892575-8114972 | 16 |
| H_09 | LG_I | 279-280.8 | 279.4 | 279.0-280.9 | Ptr_13_SSR60 | 3.1 | 0.030 | Chr13:7892575-8114972 | 16 |
| H_10 | LG_I | 279-280.8 | 279.4 | 279.0-280.9 | Ptr_13_SSR60 | 3.1 | 0.030 | Chr13:7892575-8114972 | 16 |
| H_11 | LG_I | 279-280.8 | 279.4 | 279.0-280.9 | Ptr_13_SSR60 | 3.1 | 0.030 | Chr13:7892575-8114972 | 16 |
| H_12 | LG_I | 279-280.8 | 279.4 | 279.0-280.9 | Ptr_13_SSR60 | 3.4 | 0.060 | Chr13:7892575-8114972 | 16 |
| V_01 | LG_I | 279.2-280 | 279.4 | 278.3-281.4 | Ptr_13_SSR60 | 5.9 | 0.156 | Chr13:7892575-8114972 | 16 |
| V_02 | LG_I | 279.4-279.7 | 279.4 | 278.3-281.4 | Ptr_13_SSR60 | 15.8 | 0.325 | Chr13:7892575-8114972 | 16 |
| V_03 | LG_I | 278.3-280.3 | 278.8 | 278.3-281.4 | Ptr_13_SSR60 | 14.8 | 0.381 | Chr13:7892575-8114972 | 16 |
| V_04 | LG_I | 278.3-279.9 | 278.8 | 278.3-281.4 | Ptr_13_SSR60 | 15.2 | 0.301 | Chr13:7892575-8114972 | 16 |
| V_05 | LG_I | 278.3-279.7 | 278.8 | 278.3-281.4 | Ptr_13_SSR60 | 11.6 | 0.306 | Chr13:7892575-8114972 | 16 |
| V_06 | LG_I | 278.3-281.4 | 278.8 | 278.3-281.4 | Ptr_13_SSR60 | 8.3 | 0.334 | Chr13:7892575-8114972 | 16 |
| V_07 | LG_I | 278.3-279.4 | 278.8 | 278.3-281.4 | Ptr_13_SSR60 | 7.4 | 0.276 | Chr13:7892575-8114972 | 16 |
| V_08 | LG_I | 278.3-280.5 | 278.8 | 278.3-281.4 | Ptr_13_SSR60 | 9.0 | 0.350 | Chr13:7892575-8114972 | 16 |
| V_09 | LG_I | 278.3-279 | 278.8 | 278.3-281.4 | Ptr_13_SSR60 | 7.7 | 0.293 | Chr13:7892575-8114972 | 16 |
| V_10 | LG_I | 278.3-279 | 278.8 | 278.3-281.4 | Ptr_13_SSR60 | 6.8 | 0.294 | Chr13:7892575-8114972 | 16 |
| V_11 | LG_I | 278.3-279.5 | 278.8 | 278.3-281.4 | Ptr_13_SSR60 | 4.6 | 0.255 | Chr13:7892575-8114972 | 16 |
| V_12 | LG_I | 278.3-279.3 | 278.8 | 278.3-281.4 | Ptr_13_SSR60 | 7.1 | 0.293 | Chr13:7892575-8114972 | 16 |
| V_01 | LG_II | 57.8-58.3 | 58.3 | 57.8-61.6 | Ptr_16-SSR32 | 7.5 | 0.247 | Chr16:5216365-5559330 | 27 |
| V_02 | LG_II | 57.8-61.6 | 58.3 | 57.8-61.6 | Ptr_16-SSR32 | 10.0 | 0.247 | Chr16:5216365-5559330 | 27 |
| V_03 | LG_II | 58.3-58.9 | 58.4 | 57.8-61.6 | Ptr_16-SSR32 | 7.3 | 0.166 | Chr16:5216365-5559330 | 27 |
| V_04 | LG_II | 57.8-59.6 | 58.3 | 57.8-61.6 | Ptr_16-SSR32 | 8.6 | 0.229 | Chr16:5216365-5559330 | 27 |
| V_05 | LG_II | 57.8-59.1 | 58.3 | 57.8-61.6 | Ptr_16-SSR32 | 8.7 | 0.247 | Chr16:5216365-5559330 | 27 |
| V_06 | LG_II | 57.8-59.8 | 58.3 | 57.8-61.6 | Ptr_16-SSR32 | 9.3 | 0.228 | Chr16:5216365-5559330 | 27 |
| V_07 | LG_II | 57.8-58.8 | 58.3 | 57.8-61.6 | Ptr_16-SSR32 | 7.6 | 0.204 | Chr16:5216365-5559330 | 27 |
| V_08 | LG_II | 57.8-58.3 | 58.3 | 57.8-61.6 | Ptr_16-SSR32 | 7.6 | 0.198 | Chr16:5216365-5559330 | 27 |
| V_09 | LG_II | 57.8-58.3 | 58.3 | 57.8-61.6 | Ptr_16-SSR32 | 8.8 | 0.223 | Chr16:5216365-5559330 | 27 |
| V_10 | LG_II | 58.8-59.2 | 58.9 | 57.8-61.6 | Ptr_16-SSR32 | 6.2 | 0.336 | Chr16:5216365-5559330 | 27 |
| V_11 | LG_II | 57.8-58.3 | 58.3 | 57.8-61.6 | Ptr_16-SSR32 | 6.8 | 0.185 | Chr16:5216365-5559330 | 27 |
| V_12 | LG_II | 57.8-58.3 | 58.3 | 57.8-61.6 | Ptr_16-SSR32 | 9.7 | 0.204 | Chr16:5216365-5559330 | 27 |
| V_01 | LG_III | 93.1-94.3 | 93.2 | 92.4-94.7 | Ptr_13_SSR22 | 6.9 | 0.318 | Chr13:2271530-2328111 | 12 |
| V_04 | LG_III | 92.4-93.1 | 92.7 | 92.4-94.7 | Ptr_13_SSR22 | 10.6 | 0.212 | Chr13:2271530-2328111 | 12 |
| V_05 | LG_III | 92.4-94.7 | 92.7 | 92.4-94.7 | Ptr_13_SSR22 | 10.3 | 0.216 | Chr13:2271530-2328111 | 12 |
| V_06 | LG_III | 92.4-93.3 | 92.7 | 92.4-94.7 | Ptr_13_SSR22 | 10.2 | 0.169 | Chr13:2271530-2328111 | 12 |
| V_04 | LG_III | 107.5-108.1 | 107.5 | 107.4-111.7 | Ptr_15_SSR27 | 10.1 | 0.386 | Chr08:7141332-7788190 | 82 |
| V_08 | LG_III | 119.1-120.9 | 119.6 | 119.1-120.9 | Ptr_8_SSR28 | 3.5 | 0.198 | Chr08:5712273-5983057 | 45 |
| V_09 | LG_III | 119.1-120.5 | 119.6 | 119.1-120.9 | Ptr_8_SSR28 | 5.3 | 0.207 | Chr08:5712273-5983057 | 45 |
| V_10 | LG_III | 119.1-120.6 | 119.6 | 119.1-120.9 | Ptr_8_SSR28 | 5.0 | 0.209 | Chr08:5712273-5983057 | 45 |
| V_11 | LG_III | 119.1-120.5 | 119.6 | 119.1-120.9 | Ptr_8_SSR28 | 7.2 | 0.308 | Chr08:5712273-5983057 | 45 |
| V_12 | LG_III | 119.1-120.4 | 119.6 | 119.1-120.9 | Ptr_8_SSR28 | 4.6 | 0.246 | Chr08:5712273-5983057 | 45 |
| V_01 | LG_III | 107.9-109.1 | 108.5 | 107.4-111.7 | Ptr_8_SSR33 | 6.7 | 0.161 | Chr08:7141332-7788190 | 82 |
| V_02 | LG_III | 108.4-108.8 | 108.5 | 107.4-111.7 | Ptr_8_SSR33 | 14.4 | 0.249 | Chr08:7141332-7788190 | 82 |
| V_03 | LG_III | 107.4-108 | 107.9 | 107.4-111.7 | Ptr_8_SSR33 | 14.1 | 0.308 | Chr08:7141332-7788190 | 82 |
| V_05 | LG_III | 107.9-109.4 | 108.4 | 107.4-111.7 | Ptr_8_SSR33 | 7.8 | 0.198 | Chr08:7141332-7788190 | 82 |
| V_06 | LG_III | 107.9-110.3 | 108.4 | 107.4-111.7 | Ptr_8_SSR33 | 8.8 | 0.187 | Chr08:7141332-7788190 | 82 |
| V_07 | LG_III | 107.9-109.3 | 108.5 | 107.4-111.7 | Ptr_8_SSR33 | 5.0 | 0.173 | Chr08:7141332-7788190 | 82 |
| V_08 | LG_III | 107.4-111.7 | 107.9 | 107.4-111.7 | Ptr_8_SSR33 | 6.7 | 0.294 | Chr08:7141332-7788190 | 82 |
| V_09 | LG_III | 107.4-109.8 | 107.9 | 107.4-111.7 | Ptr_8_SSR33 | 9.9 | 0.295 | Chr08:7141332-7788190 | 82 |
| V_10 | LG_III | 107.4-109.1 | 107.9 | 107.4-111.7 | Ptr_8_SSR33 | 9.3 | 0.303 | Chr08:7141332-7788190 | 82 |
| V_11 | LG_III | 107.4-109 | 107.9 | 107.4-111.7 | Ptr_8_SSR33 | 11.4 | 0.346 | Chr08:7141332-7788190 | 82 |
| V_12 | LG_III | 107.4-109.8 | 107.9 | 107.4-111.7 | Ptr_8_SSR33 | 12.0 | 0.355 | Chr08:7141332-7788190 | 82 |
| V_11 | LG_IV | 131.3-132.1 | 132.0 | 131.3-132.1 | E54/M31-362 | 3.3 | 0.035 | / | / |
| H_06 | LG_IV | 121.4-122.5 | 121.7 | 121.4-122.5 | E60/M44-390 | 3.0 | 0.028 | / | / |
| H_07 | LG_IV | 121.5-122.5 | 121.7 | 121.4-122.5 | E60/M44-390 | 3.4 | 0.032 | / | / |
| H_08 | LG_IV | 121.4-122.5 | 121.7 | 121.4-122.5 | E60/M44-390 | 3.0 | 0.028 | / | / |
| H_09 | LG_IV | 121.5-122.5 | 121.7 | 121.4-122.5 | E60/M44-390 | 3.4 | 0.072 | / | / |
| H_10 | LG_IV | 121.4-122.5 | 121.7 | 121.4-122.5 | E60/M44-390 | 3.0 | 0.028 | / | / |
| H_11 | LG_IV | 121.5-122.5 | 121.7 | 121.4-122.5 | E60/M44-390 | 4.4 | 0.062 | / | / |
| H_12 | LG_IV | 121.5-122.5 | 121.7 | 121.4-122.5 | E60/M44-390 | 3.4 | 0.032 | / | / |
| V_02 | LG_IV | 27.5-28.4 | 28.0 | 27.5-28.4 | Pto_SSR1812 | 7.2 | 0.295 | / | / |
| V_04 | LG_IV | 27.5-28.1 | 28.0 | 27.5-28.4 | Pto_SSR1812 | 4.5 | 0.228 | / | / |
| V_06 | LG_IV | 27.5-28.1 | 28.0 | 27.5-28.4 | Pto_SSR1812 | 3.3 | 0.175 | / | / |
| V_08 | LG_IV | 27.5-28.1 | 28.0 | 27.5-28.4 | Pto_SSR1812 | 5.0 | 0.233 | / | / |
| V_09 | LG_IV | 27.5-28 | 28.0 | 27.5-28.4 | Pto_SSR1812 | 5.7 | 0.266 | / | / |
| V_10 | LG_IV | 27.5-28.1 | 28.0 | 27.5-28.4 | Pto_SSR1812 | 5.5 | 0.264 | / | / |
| V_11 | LG_IV | 27.5-28.2 | 28.0 | 27.5-28.4 | Pto_SSR1812 | 3.3 | 0.209 | / | / |
| V_12 | LG_IV | 27.5-28.2 | 28.0 | 27.5-28.4 | Pto_SSR1812 | 3.3 | 0.259 | / | / |
| V_05 | LG_IV | 72.8-73.8 | 73.3 | 72.8-73.8 | Ptr_14_SSR18 | 6.1 | 0.368 | Chr14:1725402-1735475 | 2 |
| V_06 | LG_IV | 61.7-62.7 | 62.2 | 61.7-62.7 | Ptr_14_SSR55 | 3.9 | 0.113 | Chr14:9333872-9441896 | 12 |
| V_01 | LG_IV | 79.8-81.3 | 80.2 | 79.8-81.4 | Ptr_14_SSR60 | 8.5 | 0.235 | Chr14:9973141-10026379 | 9 |
| V_02 | LG_IV | 79.8-80.5 | 80.2 | 79.8-81.4 | Ptr_14_SSR60 | 15.1 | 0.292 | Chr14:9973141-10026379 | 9 |
| V_03 | LG_IV | 79.8-80.7 | 80.2 | 79.8-81.4 | Ptr_14_SSR60 | 11.1 | 0.256 | Chr14:9973141-10026379 | 9 |
| V_04 | LG_IV | 80.2-80.5 | 80.2 | 79.8-81.4 | Ptr_14_SSR60 | 8.3 | 0.162 | Chr14:9973141-10026379 | 9 |
| V_05 | LG_IV | 79.8-80.5 | 80.2 | 79.8-81.4 | Ptr_14_SSR60 | 7.4 | 0.171 | Chr14:9973141-10026379 | 9 |
| V_06 | LG_IV | 79.8-80.5 | 80.2 | 79.8-81.4 | Ptr_14_SSR60 | 8.6 | 0.139 | Chr14:9973141-10026379 | 9 |
| V_07 | LG_IV | 79.8-81.4 | 80.2 | 79.8-81.4 | Ptr_14_SSR60 | 6.6 | 0.246 | Chr14:9973141-10026379 | 9 |
| V_08 | LG_IV | 79.8-80.6 | 80.2 | 79.8-81.4 | Ptr_14_SSR60 | 7.0 | 0.189 | Chr14:9973141-10026379 | 9 |
| V_09 | LG_IV | 79.8-81.2 | 80.2 | 79.8-81.4 | Ptr_14_SSR60 | 5.9 | 0.193 | Chr14:9973141-10026379 | 9 |
| V_10 | LG_IV | 80.2-80.9 | 80.7 | 79.8-81.4 | Ptr_14_SSR60 | 6.7 | 0.365 | Chr14:9973141-10026379 | 9 |
| V_11 | LG_IV | 79.8-80.7 | 80.2 | 79.8-81.4 | Ptr_14_SSR60 | 3.6 | 0.177 | Chr14:9973141-10026379 | 9 |
| V_12 | LG_IV | 79.8-80.8 | 80.2 | 79.8-81.4 | Ptr_14_SSR60 | 6.5 | 0.241 | Chr14:9973141-10026379 | 9 |
| H_09 | LG_IX | 59.8-61.2 | 60.6 | 59.8-61.2 | E34/M42-132 | 3.5 | 0.043 | / | / |
| H_10 | LG_IX | 59.8-61.2 | 60.6 | 59.8-61.2 | E34/M42-132 | 4.4 | 0.038 | / | / |
| H_11 | LG_IX | 59.8-61.2 | 60.6 | 59.8-61.2 | E34/M42-132 | 5.4 | 0.061 | / | / |
| V_10 | LG_IX | 95.5-97.3 | 96.1 | 95.5-97.3 | E68/M92-373 | 3.2 | 0.033 | / | / |
| V_01 | LG_IX | 54.1-55.3 | 54.2 | 53.6-55.9 | Ptr_18_SSR46 | 3.3 | 0.175 | Chr18:11180589-11303079 | 12 |
| V_02 | LG_IX | 54.1-54.6 | 54.2 | 53.6-55.9 | Ptr_18_SSR46 | 11.6 | 0.271 | Chr18:11180589-11303079 | 12 |
| V_03 | LG_IX | 54.1-54.7 | 54.2 | 53.6-55.9 | Ptr_18_SSR46 | 9.9 | 0.211 | Chr18:11180589-11303079 | 12 |
| V_04 | LG_IX | 53.6-55.2 | 54.1 | 53.6-55.9 | Ptr_18_SSR46 | 9.5 | 0.288 | Chr18:11180589-11303079 | 12 |
| V_05 | LG_IX | 54.2-55.9 | 54.2 | 53.6-55.9 | Ptr_18_SSR46 | 3.4 | 0.106 | Chr18:11180589-11303079 | 12 |
| V_06 | LG_IX | 54.2-54.4 | 54.2 | 53.6-55.9 | Ptr_18_SSR46 | 6.9 | 0.098 | Chr18:11180589-11303079 | 12 |
| V_07 | LG_IX | 54.1-54.5 | 54.2 | 53.6-55.9 | Ptr_18_SSR46 | 4.2 | 0.146 | Chr18:11180589-11303079 | 12 |
| V_08 | LG_IX | 54.1-55.4 | 54.2 | 53.6-55.9 | Ptr_18_SSR46 | 3.4 | 0.116 | Chr18:11180589-11303079 | 12 |
| V_09 | LG_IX | 54.1-55 | 54.2 | 53.6-55.9 | Ptr_18_SSR46 | 4.5 | 0.132 | Chr18:11180589-11303079 | 12 |
| V_10 | LG_IX | 54.1-55.4 | 54.2 | 53.6-55.9 | Ptr_18_SSR46 | 3.2 | 0.107 | Chr18:11180589-11303079 | 12 |
| V_12 | LG_IX | 54.1-54.8 | 54.2 | 53.6-55.9 | Ptr_18_SSR46 | 5.4 | 0.120 | Chr18:11180589-11303079 | 12 |
| V_02 | LG_IX | 65.7-67.5 | 66.2 | 65.0-69.2 | Ptr_8_SSR36 | 5.8 | 0.300 | Chr08:7750461-7943204 | 20 |
| V_03 | LG_IX | 65-65.9 | 65.5 | 65.0-69.2 | Ptr_8_SSR36 | 5.4 | 0.278 | Chr08:7750461-7943204 | 20 |
| V_04 | LG_IX | 65.5-65.9 | 65.7 | 65.0-69.2 | Ptr_8_SSR36 | 3.1 | 0.126 | Chr08:7750461-7943204 | 20 |
| V_08 | LG_IX | 65.7-69.2 | 66.2 | 65.0-69.2 | Ptr_8_SSR36 | 3.2 | 0.287 | Chr08:7750461-7943204 | 20 |
| V_09 | LG_IX | 65.5-66.4 | 65.7 | 65.0-69.2 | Ptr_8_SSR36 | 3.1 | 0.074 | Chr08:7750461-7943204 | 20 |
| V_10 | LG_IX | 65.7-68.5 | 66.2 | 65.0-69.2 | Ptr_8_SSR36 | 3.4 | 0.220 | Chr08:7750461-7943204 | 20 |
| V_12 | LG_IX | 65.7-66.4 | 65.7 | 65.0-69.2 | Ptr_8_SSR36 | 6.5 | 0.120 | Chr08:7750461-7943204 | 20 |
| V_02 | LG_V | 37.6-38.1 | 38.1 | 37.1-40.0 | Ptr_17_SSR34 | 13.7 | 0.306 | Chr17:1900346-2033309 | 21 |
| V_03 | LG_V | 37.6-38.2 | 38.1 | 37.1-40.0 | Ptr_17_SSR34 | 12.4 | 0.345 | Chr17:1900346-2033309 | 21 |
| V_04 | LG_V | 37.1-39 | 37.6 | 37.1-40.0 | Ptr_17_SSR34 | 3.7 | 0.334 | Chr17:1900346-2033309 | 21 |
| V_05 | LG_V | 37.6-38.6 | 38.1 | 37.1-40.0 | Ptr_17_SSR34 | 9.6 | 0.346 | Chr17:1900346-2033309 | 21 |
| V_06 | LG_V | 37.6-38.8 | 38.1 | 37.1-40.0 | Ptr_17_SSR34 | 6.6 | 0.25 | Chr17:1900346-2033309 | 21 |
| V_07 | LG_V | 37.1-37.9 | 37.6 | 37.1-40.0 | Ptr_17_SSR34 | 4.2 | 0.245 | Chr17:1900346-2033309 | 21 |
| V_08 | LG_V | 38.4-39.9 | 38.9 | 37.1-40.0 | Ptr_17_SSR34 | 7.4 | 0.346 | Chr17:1900346-2033309 | 21 |
| V_09 | LG_V | 38.4-40 | 38.9 | 37.1-40.0 | Ptr_17_SSR34 | 9.7 | 0.349 | Chr17:1900346-2033309 | 21 |
| V_10 | LG_V | 38.4-39.4 | 38.9 | 37.1-40.0 | Ptr_17_SSR34 | 11.8 | 0.354 | Chr17:1900346-2033309 | 21 |
| V_11 | LG_V | 38.9-39.8 | 39.4 | 37.1-40.0 | Ptr_17_SSR34 | 9.1 | 0.381 | Chr17:1900346-2033309 | 21 |
| V_12 | LG_V | 37.6-38.2 | 38.1 | 37.1-40.0 | Ptr_17_SSR34 | 9.8 | 0.333 | Chr17:1900346-2033309 | 21 |
| BD_01 | LG_VI | 7.5-8.4 | 8.0 | 7.5-8.4 | Ptr_12_SSR33 | 5.4 | 0.077 | Chr12:4390642-4903494 | 36 |
| BD_02 | LG_VI | 7.5-8.4 | 8.0 | 7.5-8.4 | Ptr_12_SSR33 | 4.7 | 0.068 | Chr12:4390642-4903494 | 36 |
| BD_03 | LG_VI | 7.5-8.4 | 8.2 | 7.5-8.4 | Ptr_12_SSR33 | 3.2 | 0.156 | Chr12:4390642-4903494 | 36 |
| BD_04 | LG_VI | 7.5-8.4 | 8.0 | 7.5-8.4 | Ptr_12_SSR33 | 4.4 | 0.047 | Chr12:4390642-4903494 | 36 |
| BD_05 | LG_VI | 7.5-8.4 | 8.0 | 7.5-8.4 | Ptr_12_SSR33 | 3.7 | 0.038 | Chr12:4390642-4903494 | 36 |
| H_01 | LG_VI | 7.8-9.7 | 8.2 | 7.8-9.7 | Ptr_12_SSR33 | 3.9 | 0.045 | Chr12:4390642-4903494 | 36 |
| H_02 | LG_VI | 7.8-9.7 | 8.2 | 7.8-9.7 | Ptr_12_SSR33 | 3.4 | 0.033 | Chr12:4390642-4903494 | 36 |
| H_03 | LG_VI | 7.8-8.8 | 8.2 | 7.8-9.7 | Ptr_12_SSR33 | 3.0 | 0.035 | Chr12:4390642-4903494 | 36 |
| H_04 | LG_VI | 7.8-8.8 | 8.2 | 7.8-9.7 | Ptr_12_SSR33 | 4.0 | 0.075 | Chr12:4390642-4903494 | 36 |
| H_05 | LG_VI | 7.8-8.8 | 8.2 | 7.8-9.7 | Ptr_12_SSR33 | 3.1 | 0.035 | Chr12:4390642-4903494 | 36 |
| H_06 | LG_VI | 7.8-8.8 | 8.2 | 7.8-9.7 | Ptr_12_SSR33 | 3.7 | 0.035 | Chr12:4390642-4903494 | 36 |
| V_01 | LG_VI | 7.8-9.3 | 8.2 | 7.4-9.3 | Ptr_12_SSR33 | 5.1 | 0.058 | Chr12:4390642-4903494 | 36 |
| V_02 | LG_VI | 7.4-8.3 | 8.0 | 7.4-9.3 | Ptr_12_SSR33 | 4.5 | 0.067 | Chr12:4390642-4903494 | 36 |
| V_03 | LG_VI | 7.4-8.4 | 8.0 | 7.4-9.3 | Ptr_12_SSR33 | 3.6 | 0.056 | Chr12:4390642-4903494 | 36 |
| V_07 | LG_VI | 7.8-9.3 | 8.2 | 7.4-9.3 | Ptr_12_SSR33 | 5.1 | 0.057 | Chr12:4390642-4903494 | 36 |
| V_03 | LG_VI | 107.5-108.1 | 107.5 | 107.5-108.1 | Ptr_15_SSR27 | 13.5 | 0.390 | Chr15:5126325-5188211 | 4 |
| V_04 | LG_VI | 107.5-108.1 | 108.4 | 107.5-108.1 | Ptr_15_SSR27 | 9.9 | 0.225 | Chr15:5126325-5188211 | 4 |
| V_04 | LG_VI | 144.9-145.5 | 145.4 | 143.8-146.1 | Ptr_15_SSR60 | 3.5 | 0.150 | Chr15:9434094-9829619 | 42 |
| V_05 | LG_VI | 144.9-146.1 | 145.4 | 143.8-146.1 | Ptr_15_SSR60 | 3.4 | 0.142 | Chr15:9434094-9829619 | 42 |
| V_08 | LG_VI | 143.8-145.5 | 144.9 | 143.8-146.1 | Ptr_15_SSR60 | 3.1 | 0.096 | Chr15:9434094-9829619 | 42 |
| BD_05 | LG_VI | 113.4-113.8 | 113.7 | 113.4-113.8 | Ptr_18_SSR1 | 3.2 | 0.039 | Chr18:50120-70716 | 3 |
| BD_06 | LG_VI | 113.4-113.8 | 113.7 | 113.4-113.8 | Ptr_18_SSR1 | 3.2 | 0.109 | Chr18:50120-70716 | 3 |
| V_01 | LG_VI | 113.4-114.5 | 113.7 | 113.4-114.5 | Ptr_18_SSR1 | 8.3 | 0.174 | Chr18:50120-70716 | 3 |
| V_02 | LG_VI | 113.4-113.7 | 113.7 | 113.4-114.5 | Ptr_18_SSR1 | 14.3 | 0.244 | Chr18:50120-70716 | 3 |
| V_03 | LG_VI | 113.4-113.7 | 113.7 | 113.4-114.5 | Ptr_18_SSR1 | 9.9 | 0.183 | Chr18:50120-70716 | 3 |
| V_04 | LG_VI | 113.7-113.7 | 113.7 | 113.4-114.5 | Ptr_18_SSR1 | 10.7 | 0.185 | Chr18:50120-70716 | 3 |
| V_05 | LG_VI | 113.4-113.7 | 113.7 | 113.4-114.5 | Ptr_18_SSR1 | 6.8 | 0.295 | Chr18:50120-70716 | 3 |
| V_06 | LG_VI | 113.4-113.7 | 113.7 | 113.4-114.5 | Ptr_18_SSR1 | 10.9 | 0.168 | Chr18:50120-70716 | 3 |
| V_07 | LG_VI | 113.4-113.8 | 113.7 | 113.4-114.5 | Ptr_18_SSR1 | 4.7 | 0.159 | Chr18:50120-70716 | 3 |
| V_11 | LG_VI | 113.7-113.8 | 113.7 | 113.4-114.5 | Ptr_18_SSR1 | 5.0 | 0.138 | Chr18:50120-70716 | 3 |
| V_12 | LG_VI | 113.7-113.8 | 113.7 | 113.4-114.5 | Ptr_18_SSR1 | 8.5 | 0.153 | Chr18:50120-70716 | 3 |
| V_02 | LG_VI | 69-69.6 | 69.5 | 69.0-75.0 | Ptr_9_SSR20 | 11.8 | 0.273 | Chr09:1929816-2097651 | 12 |
| V_03 | LG_VI | 69-69.6 | 69.5 | 69.0-75.0 | Ptr_9_SSR20 | 10.1 | 0.213 | Chr09:1929816-2097651 | 12 |
| V_04 | LG_VI | 69-69.7 | 69.5 | 69.0-75.0 | Ptr_9_SSR20 | 5.7 | 0.093 | Chr09:1929816-2097651 | 12 |
| V_05 | LG_VI | 69-69.7 | 69.5 | 69.0-75.0 | Ptr_9_SSR20 | 3.4 | 0.102 | Chr09:1929816-2097651 | 12 |
| V_06 | LG_VI | 69-69.7 | 69.5 | 69.0-75.0 | Ptr_9_SSR20 | 6.9 | 0.098 | Chr09:1929816-2097651 | 12 |
| V_07 | LG_VI | 69-69.7 | 69.5 | 69.0-75.0 | Ptr_9_SSR20 | 5.1 | 0.175 | Chr09:1929816-2097651 | 12 |
| V_08 | LG_VI | 69-69.7 | 69.5 | 69.0-75.0 | Ptr_9_SSR20 | 5.6 | 0.155 | Chr09:1929816-2097651 | 12 |
| V_09 | LG_VI | 69-69.7 | 69.5 | 69.0-75.0 | Ptr_9_SSR20 | 6.0 | 0.168 | Chr09:1929816-2097651 | 12 |
| V_10 | LG_VI | 69-69.8 | 69.5 | 69.0-75.0 | Ptr_9_SSR20 | 3.8 | 0.138 | Chr09:1929816-2097651 | 12 |
| V_11 | LG_VI | 69-75 | 69.5 | 69.0-75.0 | Ptr_9_SSR20 | 3.1 | 0.113 | Chr09:1929816-2097651 | 12 |
| V_12 | LG_VI | 69-72.8 | 69.5 | 69.0-75.0 | Ptr_9_SSR20 | 5.3 | 0.181 | Chr09:1929816-2097651 | 12 |
| V_02 | LG_VII | 137.2-137.7 | 137.7 | 137.2-138.5 | Ptr_5_SSR6 | 3.9 | 0.182 | Chr05:1480240-1499889 | 5 |
| V_03 | LG_VII | 137.2-138.5 | 137.7 | 137.2-138.5 | Ptr_5_SSR6 | 3.5 | 0.191 | Chr05:1480240-1499889 | 5 |
| H_02 | LG_X | 137.4-142.1 | 141.6 | 137.4-142.1 | E32/M32-47 | 3.1 | 0.078 | / | / |
| H_03 | LG_X | 137.4-142.1 | 141.6 | 137.4-142.1 | E32/M32-47 | 3.1 | 0.048 | / | / |
| H_04 | LG_X | 138.1-141.8 | 141.1 | 137.4-142.1 | E32/M32-47 | 4.3 | 0.031 | / | / |
| H_05 | LG_X | 138.1-141.8 | 141.1 | 137.4-142.1 | E32/M32-47 | 3.3 | 0.031 | / | / |
| H_06 | LG_X | 137.4-142.1 | 141.6 | 137.4-142.1 | E32/M32-47 | 3.1 | 0.048 | / | / |
| H_07 | LG_X | 138.1-141.8 | 141.1 | 137.4-142.1 | E32/M32-47 | 5.2 | 0.031 | / | / |
| H_07 | LG_X | 71.2-72 | 71.8 | 71.2-72.0 | E60/M81-142 | 4.3 | 0.090 | / | / |
| H_08 | LG_X | 71.2-72 | 71.8 | 71.2-72.0 | E60/M81-142 | 6.3 | 0.078 | / | / |
| H_10 | LG_X | 71.2-72 | 71.8 | 71.2-72.0 | E60/M81-142 | 3.8 | 0.062 | / | / |
| H_11 | LG_X | 71.2-72 | 71.8 | 71.2-72.0 | E60/M81-142 | 3.3 | 0.042 | / | / |
| H_12 | LG_X | 71.2-72 | 71.8 | 71.2-72.0 | E60/M81-142 | 4.2 | 0.085 | / | / |
| V_10 | LG_X | 70.5-71.8 | 71.3 | 70.5-71.8 | E60/M81-84 | 3.1 | 0.039 | / | / |
| V_11 | LG_X | 70.4-71.8 | 71.3 | 70.5-71.8 | E60/M81-84 | 2.5 | 0.037 | / | / |
| BD_03 | LG_X | 50-50.5 | 50.5 | 50.0-50.5 | Ptr_12_SSR33 | 4.2 | 0.063 | Chr03:14552483-14785339 | 32 |
| BD_02 | LG_X | 50-50.5 | 50.5 | 50.0-50.5 | Ptr_3_SSR37 | 3.1 | 0.128 | Chr03:14552483-14785339 | 32 |
| BD_05 | LG_X | 50-50.5 | 50.5 | 50.0-50.5 | Ptr_3_SSR37 | 3.1 | 0.125 | Chr03:14552483-14785339 | 32 |
| BD_06 | LG_X | 50-50.5 | 50.5 | 50.0-50.5 | Ptr_3_SSR37 | 3.4 | 0.147 | Chr03:14552483-14785339 | 32 |
| BD_07 | LG_X | 50-50.5 | 50.5 | 50.0-50.5 | Ptr_3_SSR37 | 3.4 | 0.135 | Chr03:14552483-14785339 | 32 |
| BD_08 | LG_X | 50-50.5 | 50.5 | 50.0-50.5 | Ptr_3_SSR37 | 3.9 | 0.105 | Chr03:14552483-14785339 | 32 |
| BD_08 | LG_X | 50-50.5 | 50.5 | 50.0-50.5 | Ptr_3_SSR37 | 3.4 | 0.125 | Chr03:14552483-14785339 | 32 |
| V_01 | LG_X | 50-50.5 | 50.5 | 50.0-50.5 | Ptr_3_SSR37 | 10.4 | 0.241 | Chr03:14552483-14785339 | 32 |
| V_02 | LG_X | 50-50.5 | 50.5 | 50.0-50.5 | Ptr_3_SSR37 | 17.0 | 0.280 | Chr03:14552483-14785339 | 32 |
| V_03 | LG_X | 50-50.5 | 50.5 | 50.0-50.5 | Ptr_3_SSR37 | 12.5 | 0.283 | Chr03:14552483-14785339 | 32 |
| V_04 | LG_X | 50-50.5 | 50.5 | 50.0-50.5 | Ptr_3_SSR37 | 12.6 | 0.262 | Chr03:14552483-14785339 | 32 |
| V_05 | LG_X | 50-50.5 | 50.5 | 50.0-50.5 | Ptr_3_SSR37 | 12.6 | 0.276 | Chr03:14552483-14785339 | 32 |
| V_06 | LG_X | 50-50.5 | 50.5 | 50.0-50.5 | Ptr_3_SSR37 | 12.5 | 0.267 | Chr03:14552483-14785339 | 32 |
| V_07 | LG_X | 50-50.5 | 50.5 | 50.0-50.5 | Ptr_3_SSR37 | 11.0 | 0.273 | Chr03:14552483-14785339 | 32 |
| V_08 | LG_X | 50-50.5 | 50.5 | 50.0-50.5 | Ptr_3_SSR37 | 10.4 | 0.269 | Chr03:14552483-14785339 | 32 |
| V_09 | LG_X | 50-50.5 | 50.5 | 50.0-50.5 | Ptr_3_SSR37 | 12.9 | 0.349 | Chr03:14552483-14785339 | 32 |
| V_10 | LG_X | 50-50.5 | 50.5 | 50.0-50.5 | Ptr_3_SSR37 | 9.4 | 0.298 | Chr03:14552483-14785339 | 32 |
| BD_05 | LG_XI | 68.5-69.8 | 68.9 | 68.5-69.2 | CesA9_SSR1 | 3.1 | 0.037 | Chr18:2296264-2354624 | 9 |
| BD_06 | LG_XI | 68.5-69.2 | 68.9 | 68.5-69.2 | CesA9_SSR1 | 3.3 | 0.034 | Chr18:2296264-2354624 | 9 |
| BD_07 | LG_XI | 68.5-69.1 | 68.9 | 68.5-69.2 | CesA9_SSR1 | 3.2 | 0.033 | Chr18:2296264-2354624 | 9 |
| BD_08 | LG_XI | 68.5-69.1 | 68.9 | 68.5-69.2 | CesA9_SSR1 | 3.1 | 0.031 | Chr18:2296264-2354624 | 9 |
| BD_09 | LG_XI | 68.5-69 | 68.9 | 68.5-69.2 | CesA9_SSR1 | 3.3 | 0.04 | Chr18:2296264-2354624 | 9 |
| H_01 | LG_XI | 68.8-70.1 | 68.9 | 68.5-70.1 | CesA9_SSR1 | 3.8 | 0.044 | Chr18:2296264-2354624 | 9 |
| H_02 | LG_XI | 68.8-70.1 | 68.9 | 68.5-70.1 | CesA9_SSR1 | 3.8 | 0.044 | Chr18:2296264-2354624 | 9 |
| H_03 | LG_XI | 68.5-69 | 68.9 | 68.5-70.1 | CesA9_SSR1 | 4.2 | 0.050 | Chr18:2296264-2354624 | 9 |
| H_04 | LG_XI | 68.8-69 | 68.9 | 68.5-70.1 | CesA9_SSR1 | 3.5 | 0.043 | Chr18:2296264-2354624 | 9 |
| H_05 | LG_XI | 68.5-69.9 | 68.9 | 68.5-70.1 | CesA9_SSR1 | 3.7 | 0.046 | Chr18:2296264-2354624 | 9 |
| H_06 | LG_XI | 68.5-69.8 | 68.9 | 68.5-70.1 | CesA9_SSR1 | 3.6 | 0.044 | Chr18:2296264-2354624 | 9 |
| H_07 | LG_XI | 68.8-69.9 | 68.9 | 68.5-70.1 | CesA9_SSR1 | 3.7 | 0.046 | Chr18:2296264-2354624 | 9 |
| H_08 | LG_XI | 68.5-69.2 | 68.9 | 68.5-70.1 | CesA9_SSR1 | 3.3 | 0.041 | Chr18:2296264-2354624 | 9 |
| H_09 | LG_XI | 68.8-70.1 | 68.9 | 68.5-70.1 | CesA9_SSR1 | 3.1 | 0.038 | Chr18:2296264-2354624 | 9 |
| H_10 | LG_XI | 68.5-69.7 | 68.9 | 68.5-70.1 | CesA9_SSR1 | 3.8 | 0.046 | Chr18:2296264-2354624 | 9 |
| H_11 | LG_XI | 68.5-70.1 | 68.9 | 68.5-70.1 | CesA9_SSR1 | 3.0 | 0.037 | Chr18:2296264-2354624 | 9 |
| H_12 | LG_XI | 68.5-69 | 68.9 | 68.5-70.1 | CesA9_SSR1 | 3.8 | 0.048 | Chr18:2296264-2354624 | 9 |
| V_04 | LG_XI | 68.5-69 | 68.9 | 68.5-70.0 | CesA9_SSR1 | 3.4 | 0.041 | Chr18:2296264-2354624 | 9 |
| V_05 | LG_XI | 68.5-69 | 68.9 | 68.5-70.0 | CesA9_SSR1 | 4.1 | 0.052 | Chr18:2296264-2354624 | 9 |
| V_06 | LG_XI | 68.5-70 | 68.9 | 68.5-70.0 | CesA9_SSR1 | 3.5 | 0.045 | Chr18:2296264-2354624 | 9 |
| V_08 | LG_XI | 68.5-69.1 | 68.9 | 68.5-70.0 | CesA9_SSR1 | 3.2 | 0.035 | Chr18:2296264-2354624 | 9 |
| V_09 | LG_XI | 68.5-69 | 68.9 | 68.5-70.0 | CesA9_SSR1 | 3.0 | 0.039 | Chr18:2296264-2354624 | 9 |
| V_10 | LG_XI | 68.5-69 | 68.9 | 68.5-70.0 | CesA9_SSR1 | 3.4 | 0.037 | Chr18:2296264-2354624 | 9 |
| BD_10 | LG_XI | 66.4-66.8 | 66.7 | 66.4-66.8 | E57/M35-481 | 3.1 | 0.041 | / | / |
| BD_11 | LG_XI | 66.4-66.8 | 66.7 | 66.4-66.8 | E57/M35-481 | 3.1 | 0.041 | / | / |
| V_05 | LG_XI | 66.4-66.8 | 66.7 | 66.4-66.8 | E57/M35-481 | 3.4 | 0.038 | / | / |
| V_06 | LG_XI | 66.4-66.8 | 66.7 | 66.4-66.8 | E57/M35-481 | 3.3 | 0.037 | / | / |
| V_07 | LG_XI | 66.4-66.8 | 66.7 | 66.4-66.8 | E57/M35-481 | 3.6 | 0.047 | / | / |
| V_08 | LG_XI | 66.4-66.8 | 66.7 | 66.4-66.8 | E57/M35-481 | 3.9 | 0.051 | / | / |
| V_09 | LG_XI | 66.4-66.8 | 66.7 | 66.4-66.8 | E57/M35-481 | 3.7 | 0.05 | / | / |
| V_10 | LG_XI | 66.4-66.8 | 66.7 | 66.4-66.8 | E57/M35-481 | 3.6 | 0.048 | / | / |
| V_11 | LG_XI | 66.4-66.8 | 66.7 | 66.4-66.8 | E57/M35-481 | 4.8 | 0.061 | / | / |
| V_12 | LG_XI | 66.4-66.9 | 66.7 | 66.4-66.8 | E57/M35-481 | 5.0 | 0.068 | / | / |
| BD_08 | LG_XII | 51.9-52.5 | 52.4 | 51.9-52.5 | E45/M38-183 | 5.5 | 0.066 | / | / |
| BD_08 | LG_XII | 53.8-54.8 | 54.2 | 53.8-54.8 | E45/M38-99 | 3.7 | 0.042 | / | / |
| V_05 | LG_XII | 12.6-13.9 | 13.1 | 12.6-13.9 | Ptr_13_SSR38 | 7.7 | 0.311 | Chr13:4912982-5419899 | 34 |
| V_09 | LG_XII | 12.6-13.9 | 13.1 | 12.6-13.9 | Ptr_13_SSR38 | 6.6 | 0.195 | Chr13:4912982-5419899 | 34 |
| V_10 | LG_XII | 12.6-13.8 | 13.1 | 12.6-13.9 | Ptr_13_SSR38 | 6.9 | 0.231 | Chr13:4912982-5419899 | 34 |
| V_11 | LG_XII | 12.6-13.8 | 13.1 | 12.6-13.9 | Ptr_13_SSR38 | 8.6 | 0.252 | Chr13:4912982-5419899 | 34 |
| V_12 | LG_XII | 12.6-13.9 | 13.1 | 12.6-13.9 | Ptr_13_SSR38 | 12.5 | 0.279 | Chr13:4912982-5419899 | 34 |
| V_02 | LG_XII | 86.1-86.7 | 86.6 | 86.1-86.8 | Ptr_16_SSR24 | 8.8 | 0.270 | Chr16:4224182-4331942 | 12 |
| V_03 | LG_XII | 86.1-86.7 | 86.6 | 86.1-86.8 | Ptr_16_SSR24 | 7.5 | 0.27 | Chr16:4224182-4331942 | 12 |
| V_05 | LG_XII | 86.1-86.7 | 86.6 | 86.1-86.8 | Ptr_16_SSR24 | 3.9 | 0.276 | Chr16:4224182-4331942 | 12 |
| V_06 | LG_XII | 86.1-86.8 | 86.6 | 86.1-86.8 | Ptr_16_SSR24 | 3.3 | 0.164 | Chr16:4224182-4331942 | 12 |
| V_07 | LG_XII | 86.1-86.7 | 86.6 | 86.1-86.8 | Ptr_16_SSR24 | 4.0 | 0.172 | Chr16:4224182-4331942 | 12 |
| V_08 | LG_XII | 86.1-86.7 | 86.6 | 86.1-86.8 | Ptr_16_SSR24 | 5.7 | 0.268 | Chr16:4224182-4331942 | 12 |
| V_09 | LG_XII | 86.1-86.7 | 86.6 | 86.1-86.8 | Ptr_16_SSR24 | 5.9 | 0.232 | Chr16:4224182-4331942 | 12 |
| V_10 | LG_XII | 86.1-86.7 | 86.6 | 86.1-86.8 | Ptr_16_SSR24 | 5.7 | 0.222 | Chr16:4224182-4331942 | 12 |
| V_11 | LG_XII | 86.1-86.7 | 86.6 | 86.1-86.8 | Ptr_16_SSR24 | 6.8 | 0.327 | Chr16:4224182-4331942 | 12 |
| H_07 | LG_XII | 49.9-50.7 | 50.2 | 49.9-50.7 | Ptr_16_SSR6 | 3.7 | 0.089 | Chr16:633006-942148 | 59 |
| V_09 | LG_XII | 49.9-50.4 | 50.2 | 49.9-50.7 | Ptr_16_SSR6 | 4.3 | 0.118 | Chr16:633006-942148 | 60 |
| V_10 | LG_XII | 49.9-50.7 | 50.2 | 49.9-50.7 | Ptr_16_SSR6 | 3.2 | 0.100 | Chr16:633006-942148 | 59 |
| V_12 | LG_XII | 49.9-50.6 | 50.2 | 49.9-50.7 | Ptr_16_SSR6 | 5.5 | 0.120 | Chr16:633006-942148 | 60 |
| V_04 | LG_XIII | 46.9-48.8 | 47.4 | 46.9-48.8 | Ptr_4_SSR43 | 6.1 | 0.368 | Chr04:10807670-11504932 | 49 |
| H_07 | LG_XIV | 86.4-87.5 | 86.9 | 86.4-88.3 | CesA7_SSR2/CesA7_SSR1 | 7.7 | 0.065 | Chr06:26087736-26511015 | 56 |
| H_08 | LG_XIV | 86.9-88.3 | 87.4 | 86.4-88.3 | CesA7_SSR2/CesA7_SSR1 | 7.9 | 0.091 | Chr06:26087736-26511015 | 56 |
| H_09 | LG_XIV | 86.9-97.4 | 87.4 | 86.4-88.3 | CesA7_SSR2/CesA7_SSR1 | 5.6 | 0.062 | Chr06:26087736-26511015 | 56 |
| H_10 | LG_XIV | 86.9-88.3 | 87.4 | 86.4-88.3 | CesA7_SSR2/CesA7_SSR1 | 7.9 | 0.0391 | Chr06:26087736-26511015 | 56 |
| H_11 | LG_XIV | 86.9-97.4 | 87.4 | 86.4-88.3 | CesA7_SSR2/CesA7_SSR1 | 9.6 | 0.0462 | Chr06:26087736-26511015 | 56 |
| V_01 | LG_XIV | 86.9-88.3 | 87.4 | 86.4-88.3 | CesA7_SSR2/CesA7_SSR1 | 7.4 | 0.391 | Chr06:26087736-26511015 | 56 |
| V_02 | LG_XIV | 86.4-87.5 | 86.9 | 86.4-88.3 | CesA7_SSR2/CesA7_SSR1 | 7.7 | 0.365 | Chr06:26087736-26511015 | 56 |
| V_03 | LG_XIV | 86.9-97.4 | 87.4 | 86.4-88.3 | CesA7_SSR2/CesA7_SSR1 | 9.6 | 0.362 | Chr06:26087736-26511015 | 56 |
| BD_07 | LG_XV | 75.8-79.7 | 77.2 | 76.1-79.8 | E35/M51-76 | 3.5 | 0.031 | / | / |
| BD_08 | LG_XV | 76.1-79.7 | 77.7 | 76.1-79.8 | E35/M51-76 | 4.5 | 0.054 | / | / |
| BD_09 | LG_XV | 76.3-79.8 | 78.2 | 76.1-79.8 | E35/M51-76 | 4.2 | 0.059 | / | / |
| V_11 | LG_XV | 76-81.2 | 78.7 | 76.0-81.2 | E35/M51-76 | 3.1 | 0.036 | / | / |
| BD_03 | LG_XV | 50.0-50.5 | 50.6 | 71.9-72.4 | Ptr_3_SSR37 | 3.1 | 0.128 | Chr04:4011121-4127522 | 11 |
| V_02 | LG_XV | 71.9-72.4 | 72.4 | 71.9-72.4 | Ptr_4_SSR19 | 11.3 | 0.296 | Chr04:4011121-4127522 | 11 |
| V_03 | LG_XV | 71.9-72.4 | 72.4 | 71.9-72.4 | Ptr_4_SSR19 | 13.6 | 0.27 | Chr04:4011121-4127522 | 11 |
| V_04 | LG_XV | 71.9-72.4 | 72.4 | 71.9-72.4 | Ptr_4_SSR19 | 9.2 | 0.273 | Chr04:4011121-4127522 | 11 |
| V_05 | LG_XV | 71.9-72.4 | 72.4 | 71.9-72.4 | Ptr_4_SSR19 | 5.7 | 0.258 | Chr04:4011121-4127522 | 11 |
| V_06 | LG_XV | 71.9-72.4 | 72.4 | 71.9-72.4 | Ptr_4_SSR19 | 5.5 | 0.172 | Chr04:4011121-4127522 | 11 |
| BD_02 | LG_XVI | 67.3-70 | 68.5 | 67.2-70.1 | E32/M32-40 | 4.2 | 0.079 | / | / |
| BD_03 | LG_XVI | 67.4-70 | 68.5 | 67.2-70.1 | E32/M32-40 | 4.2 | 0.048 | / | / |
| BD_04 | LG_XVI | 67.7-70.1 | 69.0 | 67.2-70.1 | E32/M32-40 | 4.3 | 0.061 | / | / |
| BD_05 | LG_XVI | 67.3-70 | 68.5 | 67.2-70.1 | E32/M32-40 | 4.2 | 0.069 | / | / |
| BD_06 | LG_XVI | 67.2-70 | 69.0 | 67.2-70.1 | E32/M32-40 | 3.5 | 0.051 | / | / |
| BD_07 | LG_XVI | 67.2-70.1 | 68.5 | 67.2-70.1 | E32/M32-40 | 3.6 | 0.061 | / | / |
| BD_10 | LG_XVI | 67.5-70 | 68.5 | 67.2-70.1 | E32/M32-40 | 3.9 | 0.039 | / | / |
| BD_11 | LG_XVI | 67.5-70 | 68.5 | 67.2-70.1 | E32/M32-40 | 3.9 | 0.073 | / | / |
| BD_12 | LG_XVI | 67.4-70 | 68.5 | 67.2-70.1 | E32/M32-40 | 4.3 | 0.081 | / | / |
| H_01 | LG_XVI | 66.4-70 | 68.0 | 66.4-70.2 | E32/M32-40 | 3.0 | 0.041 | / | / |
| H_02 | LG_XVI | 66.5-69.2 | 68.0 | 66.4-70.2 | E32/M32-40 | 4.8 | 0.038 | / | / |
| H_03 | LG_XVI | 66.5-69.2 | 68.0 | 66.4-70.2 | E32/M32-40 | 3.9 | 0.036 | / | / |
| H_04 | LG_XVI | 66.6-69.4 | 68.0 | 66.4-70.2 | E32/M32-40 | 5.1 | 0.043 | / | / |
| H_05 | LG_XVI | 66.6-69.7 | 68.0 | 66.4-70.2 | E32/M32-40 | 3.7 | 0.077 | / | / |
| V_04 | LG_XVI | 67.2-70.2 | 69.0 | 66.4-70.2 | E32/M32-40 | 5.4 | 0.041 | / | / |
| V_05 | LG_XVI | 66.6-69.7 | 68.0 | 66.4-70.2 | E32/M32-40 | 3.6 | 0.06 | / | / |
| V_06 | LG_XVI | 66.4-70 | 68.0 | 66.4-70.2 | E32/M32-40 | 3.0 | 0.051 | / | / |
| V_07 | LG_XVI | 66.6-69.4 | 68.0 | 66.4-70.2 | E32/M32-40 | 4.2 | 0.072 | / | / |
| V_08 | LG_XVI | 66.5-69.2 | 68.0 | 66.4-70.2 | E32/M32-40 | 3.9 | 0.07 | / | / |
| V_09 | LG_XVI | 66.5-69.2 | 68.0 | 66.4-70.2 | E32/M32-40 | 3.8 | 0.071 | / | / |
| V_07 | LG_XVI | 63-63.6 | 63.5 | 63.3-63.6 | E45/M38-142 | 3.3 | 0.046 | / | / |
| V_08 | LG_XVI | 63.3-63.6 | 63.5 | 63.3-63.6 | E45/M38-142 | 3.5 | 0.042 | / | / |
| V_09 | LG_XVI | 63.3-63.6 | 63.5 | 63.3-63.6 | E45/M38-142 | 3.2 | 0.045 | / | / |
| V_08 | LG_XVII | 67-68.4 | 67.5 | 67.0-68.5 | Pto_SSRU84610 | 5.0 | 0.263 | / | / |
| V_09 | LG_XVII | 67-68.1 | 67.5 | 67.0-68.5 | Pto_SSRU84610 | 8.1 | 0.32 | / | / |
| V_10 | LG_XVII | 67-68.5 | 67.5 | 67.0-68.5 | Pto_SSRU84610 | 8.6 | 0.334 | / | / |
| V_11 | LG_XVII | 67.4-68.4 | 67.5 | 67.0-68.5 | Pto_SSRU84610 | 4.3 | 0.325 | / | / |
| V_12 | LG_XVII | 67.4-68.1 | 67.5 | 67.0-68.5 | Pto_SSRU84610 | 4.0 | 0.319 | / | / |
| V_02 | LG_XVII | 64.3-64.9 | 64.8 | 64.3-64.9 | Ptr_9_SSR7 | 4.4 | 0.286 | Chr09:630909-655644 | 2 |
| V_03 | LG_XVII | 64.3-64.9 | 64.8 | 64.3-64.9 | Ptr_9_SSR7 | 5.9 | 0.304 | Chr09:630909-655644 | 2 |
| V_04 | LG_XVII | 64.3-64.9 | 64.8 | 64.3-64.9 | Ptr_9_SSR7 | 5.9 | 0.271 | Chr09:630909-655644 | 2 |
| V_02 | LG_XVIII | 54.8-55.3 | 55.3 | 54.8-55.3 | Ptr_13_SSR49 | 11.9 | 0.282 | Chr13:6383491-6496433 | 14 |

‘/’, not found; BD, basal diameter, ^a^Peak marker refers to the marker that is closest to the QTL peak, ^b^Log of odds (LOD) value at position of QTL peak, ^c^Percent of phenotypic variance explained by the QTL.

**Table S3** *Annotation of dynamic QTL and corresponding segmental homology regions for three growth traits over 12 timepoints in Populus*

| **Range of QTL interval** | **Peak marker^a^** | **Marker Position** | **QTL number per cM** | **QTL number of dynamic traits** | | | | **Segmental homology regions** | **No. genes in QTL** | | |
| --- | --- | --- | --- | --- | --- | --- | --- | --- | --- | --- | --- |
|  |  |  |  | **Total** | **BD** | **H** | **V** |  | **Genes** | **miRNAs** | **LncRNAs** |
| LG_I:71.8-75.1 | Ptr_1_SSR2 | 72.2 | 2.1 | 7 | 1 | 3 | 3 | Chr01:96484-109863 (SHR01) | 4 | 0 | 19 |
| LG_I:278-280.9 | Ptr_13_SSR60 | 279.4 | 10.3* | 30 | 9 | 9 | 12 | Chr13:7892575-8114972 (SHR15) | 16 | 1 | 2 |
| LG_II:57.8-61.6 | Ptr_16-SSR32 | 58.4 | 3.2 | 12 | 0 | 0 | 12 | Chr16:5216365-5559330 (SHR23) | 27 | 0 | 11 |
| LG_III:107.4-109.8 | Ptr_8_SSR33 | 108.5 | 5.0 | 12 | 0 | 0 | 12 | Chr08:7141332-7788190 (SHR08) | 82 | 2 | 73 |
| LG_III:119.1-120.9 | Ptr_8_SSR28 | 119.1 | 2.8 | 5 | 0 | 0 | 5 | Chr08:5712273-5983057 (SHR07) | 45 | 2 | 33 |
| LG_III:92.4-94.7 | Ptr_13_SSR22 | 92.7 | 1.7 | 4 | 0 | 0 | 4 | Chr13:2271530-2328111 (SHR12) | 12 | 1 | 16 |
| LG_IV:121.4-122.5 | E60/M44-390 | 121.7 | 6.4 | 7 | 0 | 7 | 0 | / | / | / | / |
| LG_IV:131.3-132.1 | E54/M31-362 | 132.2 | 1.3 | 1 | 0 | 0 | 1 | / | / | / | / |
| LG_IV:27.2-28.4 | Pto_SSR1812 | 28.1 | 6.7 | 8 | 0 | 0 | 8 | / | / | / | / |
| LG_IV:61.7-62.7 | Ptr_14_SSR55 | 62.2 | 1.0 | 1 | 0 | 0 | 1 | Chr14:9333872-9441896 (SHR17) | 12 | 0 | 1 |
| LG_IV:72.8-73.8 | Ptr_14_SSR18 | 72.8 | 1.0 | 1 | 0 | 0 | 1 | Chr14:1725402-1735475 (SHR16) | 2 | 0 | 11 |
| LG_IV:79.8-81.4 | Ptr_14_SSR60 | 80.2 | 7.5* | 12 | 0 | 0 | 12 | Chr14:9973141-10026379 (SHR18) | 9 | 1 | 16 |
| LG_IX:53.6-55.9 | Ptr_18_SSR46 | 54.2 | 4.8 | 11 | 0 | 0 | 11 | Chr18:11180589-11303079 (SHR27) | 12 | 0 | 7 |
| LG_IX:59.8-61.2 | E34/M42-132 | 60.6 | 2.1 | 3 | 0 | 3 | 0 | / | / | / | / |
| LG_IX:65.7-69.2 | Ptr_8_SSR36 | 65.7 | 2.0 | 7 | 0 | 0 | 7 | Chr08:7750461-7943204 (SHR08) | 20 | 2 | 7 |
| LG_IX:95.5-97.3 | E68/M92-373 | 96.1 | 0.6 | 1 | 0 | 0 | 1 | / | / | / | / |
| LG_V:37.1-39.9 | Ptr_17_SSR34 | 38.4 | 3.9 | 11 | 0 | 0 | 11 | Chr17:1900346-2033309 (SHR24) | 21 | 4 | 8 |
| LG_VI:107.5-108.1 | Ptr_15_SSR27 | 107.5 | 3.3 | 2 | 0 | 0 | 2 | Chr15:5126325-5188211 (SHR19) | 4 | 0 | 12 |
| LG_VI:113.4-114.5 | Ptr_18_SSR1 | 113.7 | 10.0* | 11 | 2 | 0 | 9 | Chr18:50120-70716 (SHR25) | 3 | 0 | 5 |
| LG_VI:143.8-146.1 | Ptr_15_SSR60 | 145.7 | 1.3 | 3 | 0 | 0 | 3 | Chr15:9434094-9829619 (SHR20) | 42 | 1 | 26 |
| LG_VI:69.0-72.8 | Ptr_9_SSR20 | 69.5 | 2.9 | 11 | 0 | 0 | 11 | Chr09:1929816-2097651 (SHR10) | 12 | 1 | 10 |
| LG_VI:7.5-9.7 | Ptr_12_SSR33 | 8.2 | 7.0* | 15 | 5 | 6 | 4 | Chr12:4390642-4903494 (SHR11) | 36 | 3 | 18 |
| LG_VII:137.2-138.5 | Ptr_5_SSR6 | 137.8 | 1.5 | 2 | 0 | 0 | 2 | Chr05:1480240-1499889 (SHR05) | 5 | 1 | 0 |
| LG_X:137.4-142.1 | E32/M32-47 | 141.1 | 1.3 | 6 | 0 | 6 | 0 | / | / | / | / |
| LG_X:50.0-55.5 | Ptr_3_SSR37 | 50.6 | 3.1 | 17 | 7 | 0 | 10 | Chr03:14552483-14785339 (SHR02) | 32 | 0 | 20 |
| LG_X:70.4-72.0 | E60/M81-84 | 70.8 | 4.4 | 7 | 0 | 5 | 2 | / | / | / | / |
| LG_XI:66.4-66.8 | E57/M35-481 | 66.7 | 25.0* | 10 | 2 | 0 | 8 | / | / | / | / |
| LG_XI:68.5-70.1 | CesA9_InDel2 | 68.9 | 14.4* | 23 | 5 | 12 | 6 | Chr18:2296264-2354624 (SHR26) | 9 | 2 | 6 |
| LG_XII:12.6-13.9 | Ptr_13_SSR38 | 12.6 | 3.8 | 5 | 0 | 0 | 5 | Chr13:4912982-5419899 (SHR13) | 34 | 0 | 20 |
| LG_XII:49.9-50.7 | Ptr_16_SSR6 | 50.2 | 5.0 | 4 | 0 | 1 | 3 | Chr16:633006-942148 (SHR21) | 60 | 3 | 24 |
| LG_XII:51.9-52.5 | E45/M38-183 | 52.6 | 1.7 | 1 | 1 | 0 | 0 | / | / | / | / |
| LG_XII:53.8-54.8 | E45/M38-99 | 54.2 | 1.0 | 1 | 1 | 0 | 0 | / | / | / | / |
| LG_XII:86.1-86.8 | Ptr_16_SSR24 | 86.6 | 12.9* | 9 | 0 | 0 | 9 | Chr16:4224182-4331942 (SHR22) | 12 | 0 | 13 |
| LG_XIII:46.9-48.8 | Ptr_4_SSR43 | 46.9 | 0.5 | 1 | 0 | 0 | 1 | Chr04:10807670-11504932 (SHR04) | 47 | 3 | 16 |
| LG_XIV:86.4-87.5 | CesA7_SSR1 | 85.8 | 7.3* | 8 | 0 | 5 | 3 | Chr06:26087736-26511015 (SHR06) | 56 | 0 | 37 |
| LG_XV:71.9-72.4 | Ptr_4_SSR19 | 72.5 | 12.0* | 6 | 1 | 0 | 5 | Chr04:4011121-4127522 (SHR03) | 11 | 0 | 5 |
| LG_XV:75.8-79.8 | E35/M51-76 | 77.2 | 1.0 | 4 | 3 | 0 | 1 | / | / | / | / |
| LG_XVI:63-63.6 | E45/M38-142 | 63.7 | 5.0 | 3 | 0 | 0 | 3 | / | / | / | / |
| LG_XVI:66.4-70.1 | E32/M32-40 | 69.9 | 5.4 | 20 | 9 | 5 | 6 | / | / | / | / |
| LG_XVII:64.3-64.9 | Ptr_9_SSR7 | 65.2 | 5.0 | 3 | 0 | 0 | 3 | Chr09:630909-655644 (SHR09) | 2 | 0 | 13 |
| LG_XVII:67-68.5 | Pto_SSRU84610 | 67 | 3.3 | 5 | 0 | 0 | 5 | / | / | / | / |
| LG_XVIII:54.8-55.3 | Ptr_13_SSR49 | 55.4 | 2.0 | 1 | 0 | 0 | 1 | Chr13:6383491-6496433 (SHR14) | 14 | 1 | 1 |
| Total | / | / | / | 311 | 46 | 62 | 203 | Chr01:96484-109863 (SHR01) | 640 | 28 | 430 |

^a^ The marker information is provided in Du *et al* (2016); *QTL hotspot, n ≥ 7.0, α < 0.05, basal diameter (BD), tree height (H), stem volume (V); centiMorgans (cM); protein-coding genes (genes), long noncoding RNA (lncRNA) and microRNA (miRNA). Number of (No.). More information on these genes is listed in Data S1–3

**Table S4** Annotation and diversity assessment of genes and SNPs within 27 segmental homology regions in the association mapping panel of Populus

| **QTL Intervals** | **SHRs** | **Genes** | **miRNA** | **lncRNA** | **Total SNPs** | **Intergenic-SNPs** | **Genic-SNPs** | **Noncoding RNA-SNPs** | **SHR Length** | **Density** | **π** | **Tajima’ D** |
| --- | --- | --- | --- | --- | --- | --- | --- | --- | --- | --- | --- | --- |
| LG_I:71.8-75.1 | Chr01:96484-109863 | 4 | 0 | 19 | 296 | 6 | 290 | 57 | 13379 | 45.2 | 0.3625 | 1.9241 |
| LG_X:50-55.5 | Chr03:14552483-14785339 | 32 | 0 | 20 | 1616 | 674 | 942 | 210 | 232856 | 144.1 | 0.3483 | 2.0921 |
| LG_XV:71.9-72.4 | Chr04:4011121-4127522 | 11 | 0 | 5 | 1723 | 935 | 788 | 125 | 116401 | 67.6 | 0.2723 | 0.9993 |
| LG_XIII:46.9-48.8 | Chr04:10807670-11504932 | 47 | 3 | 16 | 5268 | 3640 | 1628 | 184 | 697262 | 132.4 | 0.2552 | 0.8154 |
| LG_VII:137.2-138.5 | Chr05:1480240-1499889 | 5 | 1 | 0 | 241 | 63 | 178 | 0 | 19649 | 81.5 | 0.3217 | 2.0225 |
| LG_XIV:86.4-87.5 | Chr06:26087736-26511015 | 56 | 0 | 37 | 2855 | 1685 | 1170 | 296 | 423279 | 148.3 | 0.3054 | 1.2378 |
| LG_III:119.1-1209 | Chr08:5712273-5983057 | 45 | 2 | 33 | 2487 | 1262 | 1225 | 304 | 270784 | 108.9 | 0.3052 | 1.3025 |
| LG_III:107.4-109.8 | Chr08:7141332-7943204 | 102 | 4 | 80 | 9624 | 7298 | 2326 | 1050 | 801872 | 83.3 | 0.2905 | 1.1475 |
| LG_XVII:64.3-64.9 | Chr09:630909-655644 | **2** | 0 | 13 | 258 | 175 | 83 | 81 | 24735 | 95.9 | 0.4381 | 1.7758 |
| LG_VI:69-72.8 | Chr09:1929816-2097651 | 12 | 1 | 10 | 896 | 515 | 381 | 47 | 167835 | 187.3 | 0.3391 | 1.9486 |
| LG_VI:7.5-9.7 | Chr12:4390642-4903494 | 36 | 3 | 18 | 4020 | 2862 | 1158 | 193 | 512852 | 127.6 | 0.2734 | 0.9622 |
| LG_III:92.4-94.7 | Chr13:2271530-2328111 | 12 | 1 | 16 | 332 | 182 | 150 | 31 | 56581 | 170.4 | 0.2542 | 0.72 |
| LG_XII:12.6-13.9 | Chr13:4912982-5419899 | 34 | 0 | 20 | 4219 | 3181 | 1038 | 321 | 506917 | 120.2 | 0.3022 | 1.4224 |
| LG_XVIII:54.8-55.3 | Chr13:6383491-6496433 | 14 | 1 | 1 | 1164 | 763 | 401 | 1 | 112942 | 97.0 | 0.2408 | 0.8042 |
| LG_I:278-280.9 | Chr13:7892575-8114972 | 16 | 1 | 2 | 1407 | 822 | 585 | 100 | 222397 | 158.1 | 0.2723 | 0.7784 |
| LG_IV:72.8-73.8 | Chr14:1725402-1735475 | 2 | 0 | 11 | 46 | 18 | 342 | 44 | 10073 | 219.0 | 0.3103 | 1.0721 |
| LG_IV:61.7-62.7 | Chr14:9333872-9441896 | 12 | 0 | 1 | 1000 | 273 | 28 | 374 | 108024 | 108.0 | 0.333 | 2.2864 |
| LG_IV:79.8-81.4 | Chr14:9973141-10026379 | 9 | 1 | 16 | 480 | 138 | 727 | 107 | 53238 | 110.9 | 0.3155 | 1.5468 |
| LG_VI:107.5-108.1 | Chr15:5126325-5188211 | 4 | 0 | 12 | 461 | 256 | 205 | 135 | 61886 | 134.2 | 0.3407 | 1.8901 |
| LG_VI:143.8-146.1 | Chr15:9434094-9829619 | 42 | 1 | 26 | 3951 | 2546 | 1405 | 390 | 395525 | 100.1 | 0.2745 | 1.0283 |
| LG_XII:49.9-50.7 | Chr16:633006-942148 | 60 | 3 | 24 | 3033 | 1746 | 1287 | 449 | 309142 | 101.9 | 0.3081 | 1.3304 |
| LG_XII:86.1-86.8 | Chr16:4224182-4331942 | 12 | 0 | 13 | 766 | 622 | 144 | 34 | 107760 | 140.7 | 0.2687 | 0.8737 |
| LG_II:57.8-61.6 | Chr16:5216365-5559330 | 27 | 0 | 11 | 3315 | 2620 | 695 | 87 | 342965 | 103.5 | 0.2822 | 1.2852 |
| LG_V:37.1-39.9 | Chr17:1900346-2033309 | 21 | 4 | 8 | 1501 | 870 | 631 | 584 | 132963 | 88.6 | 0.2732 | 1.0925 |
| LG_VI:113.4-114.5 | Chr18:50120-70716 | **3** | 0 | 5 | 146 | 109 | 37 | 0 | 20596 | 141.1 | 0.3715 | 2.9192 |
| LG_XI:68.5-70.1 | Chr18:2296264-2354624 | 9 | 2 | 6 | 436 | 223 | 213 | 53 | 58360 | 133.9 | 0.2889 | 1.2558 |
| LG_IX:53.6-55.9 | Chr18:11180589-11303079 | 12 | 0 | 7 | 1408 | 891 | 517 | 153 | 122490 | 87.0 | 0.3253 | 1.3369 |
|  |  | 641 | 28 | 430 | 52949 | 34375 | 18574 | 5410 | 5902763 | 111.5 |  |  |

Segmental homology region (SHR), Linkage group (LG), Chromosome (Chr), Long noncoding RNA (lncRNA), microRNA (miRNA), nucleotide diversity (π)

**Table S5** Summary of potential selective signals and genes within SHRs in association mapping panel.

| **Pairwise population Sets** | **No. significant bins** | **Sig. selective regions** | **Region size (kb)** | **Sig. selective PCGs** | **Sig. selective nonRNA genes** | **No. significant SNPs** | **Average Fst** |
| --- | --- | --- | --- | --- | --- | --- | --- |
| **NE vs NW** | 84 | 40 | 0.25-13.0 (1.9) | 14 | 2 | 228 | 0.117 |
| **NW vs S** | 629 | 133 | 0.25-41.2 (6.3) | 98 | 101 | 2,462 | 0.185 |
| **NE vs S** | 534 | 89 | 0.25-17.5 (3.1) | 50 | 58 | 2,021 | 0.211 |

Southern (S), Northwestern (NW), and Northeastern (NE) climatic regions; PCGs: Protein-coding genes; Significant divergent selection is the top 1% of the empirical distribution of Fst and π values between each pairwise climatic region (NW vs. NE, NW vs. S, and NE vs. S)

**Table S6** Functional annotations of these selective protein-coding genes among all pairwise climatic regions of *Populus* (NW vs. S, NE vs. S or NW vs. NE).

| **Code** | **Pairwise regions** | **Selective Genes** | **Description** | **ATG (*Arabidopsis thaliana* gene)** | |
| --- | --- | --- | --- | --- | --- |
| 1 | NE/S | Potri.001G001200 | Nuclear pore complex protein | AT1G55540 | |
| 2 | NE/S | Potri.001G001500 | RING/U-box superfamily protein | AT5G56340 | |
| 3 | NE/S | Potri.003G124100 | DEA(D/H)-box RNA helicase family protein | AT1G63250 | |
| 4 | NE/S | Potri.003G125600 | heavy metal atpase 5 | AT1G63440 | |
| 5 | NE/S | Potri.003G125800 | glutathione S-transferase THETA 1 | AT5G41210 | |
| 6 | NE/S | Potri.004G052100 | Peroxidase superfamily protein | AT2G34060 | |
| 7 | NE/S | Potri.004G052500 | glutamate receptor 2.7 | AT2G29120 | |
| 8 | NE/S | Potri.004G120400 | 0 | AT5G39200 | |
| 9 | NE/S | Potri.004G121000 | homologue of bacterial MinE 1 | AT1G69390 | |
| 10 | NE/S | Potri.006G256400 | TRAF-like family protein | AT2G25320 | |
| 11 | NE/S | Potri.006G261100 | Leucine-rich repeat protein kinase family protein | AT1G79620 | |
| 12 | NE/S | Potri.008G091200 | Early-responsive to dehydration stress protein (ERD4) | AT1G69450 | |
| 13 | NE/S | Potri.008G091400 | Prefoldin chaperone subunit family protein | AT1G26660 | |
| 14 | NE/S | Potri.008G094100 | Cobalamin biosynthesis CobW-like protein | AT1G26520 | |
| 15 | NE/S | Potri.008G094200 | beta glucosidase 40 | AT1G26560 | |
| 16 | NE/S | Potri.008G094800 | QUASIMODO2 LIKE 2 | AT2G03480 | |
| 17 | NE/S | Potri.008G112900 | Armadillo/beta-catenin-like repeat family protein | AT1G68940 | |
| 18 | NE/S | Potri.008G117000 | Di-glucose binding protein with Leucine-rich repeat domain | AT1G25570 | |
| 19 | NE/S | Potri.008G120000 | alpha-xylosidase 1 | AT1G68560 | |
| 20 | NE/S | Potri.009G011400 | tyrosylprotein sulfotransferase | AT1G08030 | |
| 21 | NE/S | Potri.009G011500 | 0 | 0 |  |
| 22 | NE/S | Potri.012G047600 | beta-6 tubulin | AT5G12250 | |
| 23 | NE/S | Potri.012G048400 | protein tyrosine kinase family protein | AT1G73660 | |
| 24 | NE/S | Potri.012G050500 | MATE efflux family protein | AT5G52450 | |
| 25 | NE/S | Potri.013G084800 | LUC7 related protein | AT3G03340 | |
| 26 | NE/S | Potri.013G085800 | Putative adipose-regulatory protein (Seipin) | AT5G16460 | |
| 27 | NE/S | Potri.014G120900 | Vps51/Vps67 family (components of vesicular transport) protein | AT4G02030 | |
| 28 | NE/S | Potri.014G121100 | WD40/YVTN repeat-like-containing domain;Bromodomain | AT2G47410 | |
| 29 | NE/S | Potri.014G121400 | Pentatricopeptide repeat (PPR) superfamily protein | AT3G62470 | |
| 30 | NE/S | Potri.014G121500 | Signal transduction histidine kinase | AT2G47430 | |
| 31 | NE/S | Potri.014G131300 | Rhodanese/Cell cycle control phosphatase superfamily protein | AT2G21045 | |
| 32 | NE/S | Potri.015G072100 | 0 | AT5G62575 | |
| 33 | NE/S | Potri.016G014800 | Glycosyl hydrolase superfamily protein | AT2G20680 | |
| 34 | NE/S | Potri.016G016200 | UDP-glucosyl transferase 71B1 | AT3G21750 | |
| 35 | NE/S | Potri.016G016300 | UDP-Glycosyltransferase superfamily protein | AT3G21790 | |
| 36 | NE/S | Potri.016G016400 | UDP-glucosyl transferase 71B6 | AT3G21780 | |
| 37 | NE/S | Potri.016G061000 | Disease resistance-responsive (dirigent-like protein) family protein | AT1G58170 | |
| 38 | NE/S | Potri.016G061500 | Leucine-rich repeat transmembrane protein kinase | AT1G53440 | |
| 39 | NE/S | Potri.016G061600 | low-molecular-weight cysteine-rich 8 | AT3G61172 | |
| 40 | NE/S | Potri.016G071600 | Late embryogenesis abundant (LEA) hydroxyproline-rich glycoprotein family | AT2G35980 | |
| 41 | NE/S | Potri.016G072700 | alpha/beta-Hydrolases superfamily protein | AT3G11620 | |
| 42 | NE/S | Potri.017G021300 | Cysteine proteinases superfamily protein | AT4G15880 | |
| 43 | NE/S | Potri.017G022600 | 0 | 0 |  |
| 44 | NE/S | Potri.017G022700 | Plant regulator RWP-RK family protein | AT2G43500 | |
| 45 | NE/S | Potri.018G029500 | Basic-leucine zipper (bZIP) transcription factor family protein | AT5G11260 | |
| 46 | NE/S | Potri.018G085100 | novel interactor of JAZ | AT4G28910 | |
| 47 | NE/S | Potri.018G085200 | sugar transporter 14 | AT1G77210 | |
| 48 | NE/S | Potri.018G085600 | alpha/beta-Hydrolases superfamily protein | AT2G44970 | |
| 49 | NE/S | Potri.018G085800 | Tetratricopeptide repeat (TPR)-like superfamily protein | AT4G02750 | |
| 50 | NW/NE | Potri.004G052700 | RNA helicase family protein | AT4G18465 | |
| 51 | NW/NE | Potri.004G121100 | early nodulin-like protein 17 | AT5G15350 | |
| 52 | NW/NE | Potri.006G256500 | ACT-like superfamily protein | AT5G25320 | |
| 53 | NW/NE | Potri.008G113300 | zinc transporter 7 precursor | AT2G04032 | |
| 54 | NW/NE | Potri.008G117000 | Di-glucose binding protein with Leucine-rich repeat domain | AT1G25570 | |
| 55 | NW/NE | Potri.008G121400 | Tetratricopeptide repeat (TPR)-like superfamily protein | AT1G13410 | |
| 56 | NW/NE | Potri.013G085100 | O-acyltransferase (WSD1-like) family protein | AT3G49190 | |
| 57 | NW/NE | Potri.016G060600 | zinc finger protein 8 | AT2G41940 | |
| 58 | NW/NE | Potri.016G060900 | Disease resistance-responsive (dirigent-like protein) family protein | AT1G58170 | |
| 59 | NW/NE | Potri.016G061000 | Disease resistance-responsive (dirigent-like protein) family protein | AT1G58170 | |
| 60 | NW/NE | Potri.016G061100 | Translation protein SH3-like family protein | AT1G57860 | |
| 61 | NW/NE | Potri.016G071600 | Late embryogenesis abundant (LEA) hydroxyproline-rich glycoprotein family | AT2G35980 | |
| 62 | NW/NE | Potri.017G022900 | Plant regulator RWP-RK family protein | AT3G59580 | |
| 63 | NW/NE | Potri.018G085400 | Exostosin family protein | AT5G19670 | |
| 64 | NW/S | Potri.001G001200 | Nuclear pore complex protein | AT1G55540 | |
| 65 | NW/S | Potri.003G125700 | heavy metal atpase 5 | AT1G63440 | |
| 66 | NW/S | Potri.003G126700 | Protein kinase protein with tetratricopeptide repeat domain | AT1G63500 | |
| 67 | NW/S | Potri.004G051900 | GDSL-like Lipase/Acylhydrolase superfamily protein | AT5G45950 | |
| 68 | NW/S | Potri.004G052300 | Apoptosis inhibitory protein 5 (API5) | AT2G34040 | |
| 69 | NW/S | Potri.004G052500 | glutamate receptor 2.7 | AT2G29120 | |
| 70 | NW/S | Potri.005G018700 | Inorganic H pyrophosphatase family protein | AT1G15690 | |
| 71 | NW/S | Potri.006G256600 | pectin methylesterase 3 | AT3G14310 | |
| 72 | NW/S | Potri.006G257600 | Beta-1,3-N-Acetylglucosaminyltransferase family protein | AT4G32105 | |
| 73 | NW/S | Potri.006G257700 | 0 | 0 |  |
| 74 | NW/S | Potri.006G259100 | 0 | 0 |  |
| 75 | NW/S | Potri.006G259600 | aberrant lateral root formation 4 | AT5G11030 | |
| 76 | NW/S | Potri.006G261700 | C2H2 and C2HC zinc fingers superfamily protein | AT5G10970 | |
| 77 | NW/S | Potri.008G091200 | Early-responsive to dehydration stress protein (ERD4) | AT1G69450 | |
| 78 | NW/S | Potri.008G091400 | Prefoldin chaperone subunit family protein | AT1G26660 | |
| 79 | NW/S | Potri.008G091500 | 0 | AT1G26650 | |
| 80 | NW/S | Potri.008G091600 | 0 | AT3G11810 | |
| 81 | NW/S | Potri.008G091700 | DHHC-type zinc finger family protein | AT1G69420 | |
| 82 | NW/S | Potri.008G092900 | Pentatricopeptide repeat (PPR) superfamily protein | AT2G03380 | |
| 83 | NW/S | Potri.008G093100 | appr-1-p processing enzyme family protein | AT1G69340 | |
| 84 | NW/S | Potri.008G093200 | nodulin-related protein 1 | AT2G03440 | |
| 85 | NW/S | Potri.008G093300 | Remorin family protein | AT1G13920 | |
| 86 | NW/S | Potri.008G093900 | Glycosyl transferase, family 35 | AT3G29320 | |
| 87 | NW/S | Potri.008G094000 | WRKY DNA-binding protein 57 | AT1G69310 | |
| 88 | NW/S | Potri.008G094100 | Cobalamin biosynthesis CobW-like protein | AT1G26520 | |
| 89 | NW/S | Potri.008G094200 | beta glucosidase 40 | AT1G26560 | |
| 90 | NW/S | Potri.008G094400 | 0 | AT1G26580 | |
| 91 | NW/S | Potri.008G094700 | Tetratricopeptide repeat (TPR)-like superfamily protein | AT3G27960 | |
| 92 | NW/S | Potri.008G094800 | QUASIMODO2 LIKE 2 | AT2G03480 | |
| 93 | NW/S | Potri.008G095100 | 0 | AT1G26470 | |
| 94 | NW/S | Potri.008G095200 | Glucose-6-phosphate/phosphate translocator-related | AT5G46110 | |
| 95 | NW/S | Potri.008G112900 | Armadillo/beta-catenin-like repeat family protein | AT1G68940 | |
| 96 | NW/S | Potri.008G113500 | 0 | 0 |  |
| 97 | NW/S | Potri.008G113700 | 6-phosphogluconate dehydrogenase family protein | AT3G02360 | |
| 98 | NW/S | Potri.008G113900 | LAG1 longevity assurance homolog 3 | AT1G13580 | |
| 99 | NW/S | Potri.008G114300 | nudix hydrolase 1 | AT1G68760 | |
| 100 | NW/S | Potri.008G114900 | decapping 5 | AT1G26110 | |
| 101 | NW/S | Potri.008G115700 | Family of unknown function (DUF572) | AT1G25682 | |
| 102 | NW/S | Potri.008G116400 | Transmembrane Fragile-X-F-associated protein | AT1G68820 | |
| 103 | NW/S | Potri.008G116600 | NAC (No Apical Meristem) domain transcriptional regulator superfamily protein | AT1G25580 | |
| 104 | NW/S | Potri.008G117000 | Di-glucose binding protein with Leucine-rich repeat domain | AT1G25570 | |
| 105 | NW/S | Potri.008G117200 | methionine aminopeptidase 1B | AT1G13270 | |
| 106 | NW/S | Potri.008G117300 | allene oxide cyclase 3 | AT3G25780 | |
| 107 | NW/S | Potri.008G117400 | golgi alpha-mannosidase II | AT5G14950 | |
| 108 | NW/S | Potri.008G117500 | myb-like transcription factor family protein | AT1G25550 | |
| 109 | NW/S | Potri.008G117700 | phytochrome and flowering time regulatory protein (PFT1) | AT1G25540 | |
| 110 | NW/S | Potri.008G120000 | alpha-xylosidase 1 | AT1G68560 | |
| 111 | NW/S | Potri.008G120400 | B-box type zinc finger protein with CCT domain | AT1G25440 | |
| 112 | NW/S | Potri.008G120600 | LOB domain-containing protein 42 | AT1G68510 | |
| 113 | NW/S | Potri.008G120900 | Protein of unknown function (DUF1218) | AT1G13380 | |
| 114 | NW/S | Potri.008G121100 | ribosomal protein L15 | AT3G25920 | |
| 115 | NW/S | Potri.008G121200 | C2H2 and C2HC zinc fingers superfamily protein | AT1G68480 | |
| 116 | NW/S | Potri.008G121300 | Regulator of Vps4 activity in the MVB pathway protein | AT1G25420 | |
| 117 | NW/S | Potri.008G121400 | Tetratricopeptide repeat (TPR)-like superfamily protein | AT1G13410 | |
| 118 | NW/S | Potri.009G002100 | Transducin/WD40 repeat-like superfamily protein | AT4G29860 | |
| 119 | NW/S | Potri.009G011400 | tyrosylprotein sulfotransferase | AT1G08030 | |
| 120 | NW/S | Potri.009G011500 | 0 | 0 |  |
| 121 | NW/S | Potri.009G011600 | 0 | 0 |  |
| 122 | NW/S | Potri.009G011700 | isovaleryl-CoA-dehydrogenase | AT3G45300 | |
| 123 | NW/S | Potri.009G011800 | auxin response factor 4 | AT5G60450 | |
| 124 | NW/S | Potri.012G048400 | protein tyrosine kinase family protein | AT1G73660 | |
| 125 | NW/S | Potri.012G048800 | Tetratricopeptide repeat (TPR)-like superfamily protein | AT4G02750 | |
| 126 | NW/S | Potri.012G049200 | transducin family protein / WD-40 repeat family protein | AT3G18060 | |
| 127 | NW/S | Potri.012G049700 | peroxin 3 | AT1G48635 | |
| 128 | NW/S | Potri.012G050200 | glutathione S-transferase tau 7 | AT2G29420 | |
| 129 | NW/S | Potri.012G050300 | cyclin-dependent kinase D1;3 | AT1G18040 | |
| 130 | NW/S | Potri.012G050800 | 0 | 0 |  |
| 131 | NW/S | Potri.013G035000 | Pre-rRNA-processing protein TSR2, conserved region | AT5G27990 | |
| 132 | NW/S | Potri.013G066900 | WUSCHEL related homeobox 11 | AT3G03660 | |
| 133 | NW/S | Potri.013G084800 | LUC7 related protein | AT3G03340 | |
| 134 | NW/S | Potri.013G085000 | THO2 | AT1G24706 | |
| 135 | NW/S | Potri.014G018200 | PLAT/LH2 domain-containing lipoxygenase family protein | AT3G22400 | |
| 136 | NW/S | Potri.014G121400 | Pentatricopeptide repeat (PPR) superfamily protein | AT3G62470 | |
| 137 | NW/S | Potri.014G131000 | AAA-type ATPase family protein | AT4G02480 | |
| 138 | NW/S | Potri.015G070300 | DP-E2F-like 1 | AT3G48160 | |
| 139 | NW/S | Potri.016G012300 | Leucine-rich repeat transmembrane protein kinase | AT1G29730 | |
| 140 | NW/S | Potri.016G013400 | SWIB complex BAF60b domain-containing protein | AT4G22360 | |
| 141 | NW/S | Potri.016G013900 | DEA(D/H)-box RNA helicase family protein | AT5G60990 | |
| 142 | NW/S | Potri.016G014000 | Ubiquitin C-terminal hydrolases superfamily protein | AT4G22350 | |
| 143 | NW/S | Potri.016G014800 | Glycosyl hydrolase superfamily protein | AT2G20680 | |
| 144 | NW/S | Potri.016G016400 | UDP-glucosyl transferase 71B6 | AT3G21780 | |
| 145 | NW/S | Potri.016G016600 | UDP-Glycosyltransferase superfamily protein | AT3G21790 | |
| 146 | NW/S | Potri.016G060600 | zinc finger protein 8 | AT2G41940 | |
| 147 | NW/S | Potri.016G060700 | Disease resistance-responsive (dirigent-like protein) family protein | AT1G65870 | |
| 148 | NW/S | Potri.016G061000 | Disease resistance-responsive (dirigent-like protein) family protein | AT1G58170 | |
| 149 | NW/S | Potri.016G061100 | Translation protein SH3-like family protein | AT1G57860 | |
| 150 | NW/S | Potri.016G071600 | Late embryogenesis abundant (LEA) hydroxyproline-rich glycoprotein family | AT2G35980 | |
| 151 | NW/S | Potri.016G071700 | 0 | AT3G52480 | |
| 152 | NW/S | Potri.016G072700 | alpha/beta-Hydrolases superfamily protein | AT3G11620 | |
| 153 | NW/S | Potri.016G072900 | ovate family protein 13 | AT5G04820 | |
| 154 | NW/S | Potri.016G073100 | ubiquitin E2 variant 1D-4 | AT3G52560 | |
| 155 | NW/S | Potri.017G023200 | Ypt/Rab-GAP domain of gyp1p superfamily protein | AT2G43490 | |
| 156 | NW/S | Potri.018G029300 | Alba DNA/RNA-binding protein | AT2G34160 | |
| 157 | NW/S | Potri.018G029400 | cellulose synthase 1 | AT4G32410 | |
| 158 | NW/S | Potri.018G085200 | sugar transporter 14 | AT1G77210 | |
| 159 | NW/S | Potri.018G085400 | Exostosin family protein | AT5G19670 | |
| 160 | NW/S | Potri.018G085500 | Exostosin family protein | AT5G19670 | |
| 161 | NW/S | Potri.018G085800 | Tetratricopeptide repeat (TPR)-like superfamily protein | AT4G02750 | |

Three sub-populations from different climatic zones, the southern (S), north-western (NW), and north-eastern (NE) climatic regions

**Table S7**  Summary of the number of all significant SNP within SHRs associated with three growth traits over nine time points of *Populus*

| **Trait^a^** | **No. Sign. SNP ^b^** | **No. SHRs** | **Significance *P*-value ^c^** | **No. intergenic SNPs** | **No. SNP-noncoding RNAs** | **No. genic-SNP** | **No. gene^d^** |
| --- | --- | --- | --- | --- | --- | --- | --- |
| BD1-01 | 19 | 11 | 1.01749E-07 | 8 | 2 | 11 | 9 |
| BD1-02 | 15 | 11 | 4.23606E-07 | 5 | 0 | 10 | 9 |
| BD1-03 | 19 | 9 | 7.112E-08 | 7 | 2 | 12 | 10 |
| BD1-04 | 17 | 6 | 2.6594E-07 | 8 | 2 | 9 | 9 |
| BD1-05 | 78 | 16 | 5.52021E-10 | 16 | 12 | 62 | 48 |
| BD2 | 247 | 21 | 1.26178E-11 | 81 | 33 | 166 | 123 |
| BD3 | 604 | 20 | 1.48264E-13 | 319 | 79 | 285 | 179 |
| BD4 | 704 | 25 | 1.2806E-13 | 388 | 81 | 316 | 188 |
| BD5 | 477 | 23 | 4.07158E-14 | 265 | 36 | 212 | 136 |
| H1-01 | 17 | 10 | 6.9622E-07 | 5 | 4 | 12 | 13 |
| H1-02 | 20 | 12 | 8.42921E-08 | 3 | 3 | 17 | 16 |
| H1-03 | 15 | 9 | 5.73461E-08 | 3 | 1 | 12 | 12 |
| H1-04 | 17 | 10 | 3.14614E-08 | 2 | 1 | 15 | 11 |
| H1-05 | 40 | 13 | 1.75231E-07 | 14 | 7 | 26 | 21 |
| H2 | 82 | 21 | 1.40363E-08 | 30 | 9 | 52 | 40 |
| H3 | 161 | 19 | 7.04556E-09 | 71 | 20 | 90 | 53 |
| H4 | 385 | 24 | 7.13696E-09 | 221 | 48 | 164 | 99 |
| H5 | 543 | 23 | 5.45964E-10 | 307 | 71 | 236 | 144 |
| V1-01 | 217 | 23 | 1.24051E-17 | 122 | 24 | 95 | 66 |
| V1-02 | 155 | 18 | 1.36008E-09 | 71 | 18 | 84 | 57 |
| V1-03 | 28 | 15 | 2.71082E-07 | 5 | 4 | 23 | 22 |
| V1-04 | 30 | 13 | 1.19166E-08 | 5 | 5 | 25 | 20 |
| V1-05 | 84 | 14 | 8.48489E-08 | 33 | 17 | 51 | 44 |
| V2 | 358 | 25 | 1.03493E-10 | 196 | 33 | 162 | 118 |
| V3 | 776 | 25 | 2.78355E-14 | 419 | 102 | 357 | 204 |
| V4 | 703 | 25 | 1.79182E-15 | 362 | 85 | 341 | 200 |
| V5 | 776 | 25 | 1.47106E-16 | 405 | 100 | 371 | 210 |
| Total | 6587 | 466 | 2.30561E-06 | 3371 | 799 | 3216 | 2061 |

Segmental homology regions (SHRs), Basal diameter (BD), Tree height (H), Stem volume (V), untranslated region (UTR). ^a^ Each growth trait over nine time points of *Populus*. ^b^ a more stringent Bonferroni-adjust threshold of *P* < 9.4 × 10^−7^ (Significant *P*-value). ^c^ The most significant *P* value of the SNP (lead SNP) for each trait only. ^d^ all candidate gene in the locus or the nearest annotated gene to the significant SNP, position for the lead SNP according to the annotation of genes in the *Populus* genome V.3.0

**Table S8** Summary of pairwise SNP and gene epistasis for three traits at nine time points in association mapping panel of Populus

| **Traits** | **A**×**A** | **A**×**D** | **D**×**A** | **D**×**D** | **No. SNP-SNP** | **No. Gene-Gene** | **No. pairwise Gene-Noncoding RNA** | **No. additive & dominance SNPs** | **No. Gene (Network)** | **No. Noncoding RNA (Network)** |
| --- | --- | --- | --- | --- | --- | --- | --- | --- | --- | --- |
| BD1-01 | 128 | 142 | 152 | 198 | 620 | 91 | 20 | 35 | 91 | 9 |
| BD1-02 | 193 | 219 | 244 | 331 | 987 | 123 | 33 | 63 | 112 | 12 |
| BD1-03 | 112 | 169 | 150 | 185 | 616 | 53 | 25 | 36 | 69 | 9 |
| BD1-04 | 115 | 97 | 136 | 115 | 463 | 32 | 9 | 13 | 49 | 8 |
| BD1-05 | 178 | 190 | 157 | 204 | 729 | 56 | 48 | 24 | 83 | 9 |
| BD2 | 169 | 173 | 172 | 205 | 719 | 59 | 35 | 26 | 81 | 12 |
| BD3 | 330 | 264 | 293 | 240 | 1127 | 101 | 34 | 37 | 106 | 15 |
| BD4 | 1192 | 885 | 843 | 620 | 3540 | 285 | 57 | 71 | 164 | 17 |
| BD5 | 1868 | 1612 | 1507 | 1219 | 6206 | 459 | 97 | 99 | 188 | 21 |
| Total-BD | 4285 | 3751 | 3654 | 3317 | 15007 | 1259 | 358 | 404 | 943 | 112 |
| H1-01 | 42 | 53 | 51 | 90 | 236 | 28 | 9 | 12 | 42 | 7 |
| H1-02 | 80 | 30 | 33 | 26 | 169 | 24 | 8 | 9 | 39 | 7 |
| H1-03 | 18 | 21 | 20 | 26 | 85 | 10 | 0 | 3 | 19 | 0 |
| H1-04 | 55 | 37 | 41 | 23 | 156 | 14 | 2 | 5 | 20 | 2 |
| H1-05 | 142 | 49 | 142 | 71 | 404 | 66 | 10 | 17 | 71 | 7 |
| H2 | 461 | 513 | 444 | 340 | 1758 | 130 | 29 | 43 | 119 | 12 |
| H3 | 430 | 476 | 423 | 303 | 1632 | 158 | 42 | 32 | 118 | 13 |
| H4 | 766 | 783 | 951 | 680 | 3180 | 295 | 59 | 38 | 168 | 20 |
| H5 | 522 | 523 | 555 | 409 | 2009 | 171 | 30 | 40 | 122 | 13 |
| Total-H | 2516 | 2485 | 2660 | 1968 | 9629 | 896 | 189 | 199 | 718 | 81 |
| V1-01 | 2100 | 6997 | 7356 | 15109 | 31562 | 2692 | 758 | 165 | 282 | 29 |
| V1-02 | 341 | 1573 | 1690 | 4207 | 7811 | 700 | 235 | 120 | 216 | 18 |
| V1-03 | 7 | 5 | 14 | 25 | 51 | 7 | 1 | 4 | 12 | 1 |
| V1-04 | 0 | 0 | 0 | 0 | 0 | 0 | 0 | 0 | 0 | 0 |
| **V1-05** | 16 | 40 | 43 | 115 | 214 | 28 | 15 | 14 | 48 | 6 |
| **V2** | 103 | 90 | 85 | 123 | 401 | 24 | 8 | 26 | 39 | 4 |
| **V3** | 157 | 74 | 105 | 76 | 412 | 33 | 8 | 23 | 50 | 4 |
| **V4** | 378 | 152 | 171 | 96 | 797 | 102 | 18 | 39 | 96 | 11 |
| **V5** | 419 | 165 | 212 | 57 | 853 | 113 | 23 | 28 | 102 | 12 |
| **Total-V** | 3521 | 9096 | 9676 | 19808 | 42101 | 3699 | 1066 | 419 | 845 | 85 |

Additive × Additive (A × A), Additive × Dominance (A × D), Dominance × Additive (D × A), Dominance × Dominance (D × D), Basal diameter (BD), Tree height (H), Stem volume (V). The significant threshold of *P* < 1e-03. Number of (No.)

**Table S9**  The mean value for each trait at the nine time point in the three climatic regions of the association mapping panel

| **Trait** | **T1-01** | **T1-02** | **T1-03** | **T1-04** | **T1-05** | **T2** | **T3** | **T4** | **T5** |
| --- | --- | --- | --- | --- | --- | --- | --- | --- | --- |
| NE_H | 1.177 | 1.661 | 2.302 | 2.838 | 4.089 | 5.526 | 6.657 | 8.291 | 10.059 |
| NW_H | 1.156 | 1.698 | 2.285 | 2.780 | 3.951 | 5.278 | 6.401 | 8.044 | 10.042 |
| S_H | 1.346 | 1.901 | 2.484 | 2.957 | 4.140 | 5.482 | 6.591 | 8.137 | 10.019 |
| NE_BD | 1.132 | 1.605 | 2.487 | 3.317 | 4.372 | 5.603 | 7.386 | 8.982 | 11.309 |
| NW_BD | 1.270 | 1.717 | 2.478 | 3.233 | 4.279 | 5.481 | 7.250 | 8.746 | 10.894 |
| S_BD | 1.094 | 1.562 | 2.475 | 3.395 | 4.345 | 5.507 | 7.261 | 8.749 | 11.215 |
| NE_V | 1.79E-04 | 4.32E-04 | 1.26E-03 | 2.71E-03 | 6.51E-03 | 1.51E-02 | 3.09E-02 | 5.57E-02 | 1.07E-01 |
| NW_V | 2.49E-04 | 5.86E-04 | 1.36E-03 | 2.69E-03 | 6.42E-03 | 1.41E-02 | 2.97E-02 | 5.36E-02 | 1.04E-01 |
| S_V | 2.12E-04 | 4.84E-04 | 1.34E-03 | 2.89E-03 | 6.39E-03 | 1.41E-02 | 2.93E-02 | 5.23E-02 | 1.04E-01 |

Basal diameter (BD), Tree height (H), Stem volume (V); Southern (S), Northwestern (NW), and Northeastern (NE) climatic regions.

**Table S10** Primers used for reverse transcription quantitative PCR for the candidate genes

| **Code** | **Gene_ID** | **F_Primer (5'-3')** | **R_Primer (5'-3')** | **Product size** | **Tm** |
| --- | --- | --- | --- | --- | --- |
| 1 | *Potri.004G116700* | CTCCGTCGTCCGTTTCACCT | CCAACCACCTATTCCTCACC | 122 | 59 |
| 2 | *Potri.004G120800* | CGGAGACAGACAGGGTAGAA | GTCAGCGAAGAAACTGGAGA | 290 | 55 |
| 3 | *Potri.004G120900* | GCTGGCATGTTAGAAACTGT | TATGAAAGGCTCTTGCTCGT | 163 | 54 |
| 4 | *Potri.006G261000* | TTGAAGCAAGCAGCACGGATGT | AGGCTGGTAACTGCAAGGATAAGG | 89 | 64 |
| 5 | *Potri.008G095300* | TGGGCACTCACTGGTCATTC | CAACAGCTTGGCCTTTCTTC | 214 | 58 |
| 6 | *Potri.008G113300* | TTGTCGTCCGATTGTTTGCC | TTCGCTTTCTGATAACCATTTCC | 173 | 61 |
| 7 | *Potri.008G114200* | TCCATTAACCTGCACGCCAATA | TTCCAACCCTCATGCCGATC | 255 | 63 |
| 8 | *Potri.008G119100* | TCTGAAAGCAGCGAGGACAA | CAATTACACTACGGGAACGA | 136 | 56 |
| 9 | *Potri.008G120600* | AACACCTACGACATACGCCACG | GACCCAAACATGAATCACGAACC | 181 | 63 |
| 10 | *Potri.008G121100* | CTTCCTTGCCCTTCTTCT | TGCTTGATTGACTACCACC | 123 | 51 |
| 11 | *Potri.008G121200* | TCTTACTCTTCAACACTCCCTT | TCTTTCCCATCCTTAGCG | 170 | 53 |
| 12 | *Potri.016G012300* | AATCGGTTTGTTCGGTCAC | CAGCGTCGCAGGAATCTC | 143 | 56 |
| 13 | *Potri.016G012400* | AGCAAGAAGAAAGCCACA | TACAAAGCCGAATCGAAC | 300 | 51 |
| 14 | *Potri.016G013200* | TGGAGACTAAACTGGAGCAA | GCTGCCTAATACATCCCT | 287 | 51 |
| 15 | *Potri.016G013500* | AGAGGAGGGAGGAAGCAG | ACTGAACTAGGAACGGCA | 184 | 54 |
| 16 | *Potri.016G060600* | CCCCACATACTAATAATCCC | GAGTCTCCCTTTCAACCTTA | 183 | 51 |
| 17 | *Potri.016G073400* | GGCTGCTTGATTTCCTCCCT | GCTACTGGCACTCCGCTTTT | 265 | 60 |
| 18 | *Potri.016G073500* | ACTAGGAGATGATCCCTGTTAT | ATCTACCCACCTCTTTCACTAA | 193 | 52 |
| 19 | *TCONS_00078983* | GCTTGGGTTATCTGGGTTCAAG | AAGACGCAGCGACTGGTAG | 165 | 56 |
| 20 | *TCONS_00138008* | CAATGAATGCCACAAGGTCTAGTT | AGAGAGAGGTGCGGTAAGTTT | 255 | 63 |
| 21 | *TCONS_00146931* | GGCGCCTCATGGAAACAA | ACTAACCAACGACGACCTCT | 136 | 56 |
| 22 | *TCONS_00153166* | GTCCTGTGAAGCTCTTTGGA | ACGTAGGTCTTCACCAGCTAA | 181 | 63 |
| 23 | *TCONS_00140766* | TCCCCCACAGCCATGAAT | TGTTGAGGTAGCAAAGATTCCTCT | 123 | 51 |
| 24 | *TCONS_00141932* | TCTAGTCCAGTGAAGCTTTTTGGA | GTCTTCGCTAACTTATCGTCCT | 170 | 53 |
| 25 | *Actin* | TTCATTTCACATCTTCCCCTTTTT | TGTCTGCGGGTGTGTCTCTAG | 173 | 61 |

**Methods S1 Identification and target prediction of noncoding RNA genes**

The secondary structures of the miRNAs and lncRNAs were predicted with the Vienna RNA package RNAfold website (<http://rna.tbi.univie.ac.at/>). Two independent algorithms were used to predict the potential target genes of lncRNAs according to their *cis*- and *trans*- regulatory actions. The first algorithm searches for potential *cis* target genes that are physically close to the lncRNAs (within 10 kb) by using a genome browser. The genes transcribed within a 10-kb window upstream or downstream of lncRNAs were considered as potential *cis* target genes (Jia *et al*., 2010). The second algorithm searches for potential *trans* targets in the *Populus* mRNA database and is based on mRNA sequence complementarity and RNA duplex energy prediction, assessing the impact of lncRNA binding to complete mRNA molecules.

First, we used BLAST to select target sequences that were complementary to the lncRNA, setting E-value < 1e-5 and identity ≥ 95%. Then we used the RNAplex software to calculate the complementary energy between two sequences for further screening and to select potential *trans*-acting target genes (RNAplex-e-60) (Tafer and Hofacker, 2008). Prediction of miRNA target genes was performed by psRNATarget (<http://plantgrn.noble.org/psRNATarget/>) via complementary base pair interactions, with expectation ≤ 5.0.

**Method S2 Library construction for genome re-sequencing**

Briefly, total DNA was randomly sheared into small fragments (200–300 bp) using a Covaris E210 ultrasonicator (Covaris, Inc., Woburn, MA, USA), followed by the overhangs converting into blunt ends using T4 DNA polymerase and DNA polymerase I Klenow fragment. After adenylation of 3′ ends of DNA fragments, Illumina adaptors (Illumina, San Diego, CA, USA) were then ligated to the ends of these DNA fragments using DNA ligase. DNA fragments that have adaptor molecules on both ends were selectively enriched using Illumina PCR Primer Cocktail and PCR products were purified (AMPure XP system) and quantified using the Agilent high-sensitivity DNA assay on the Agilent Bioanalyzer 2100 system. Finally, short-read sequence data were generated for each individual tree using an Illumina Genome Analyzer. Raw sequence data of genome re-sequencing have been deposited in the Genome Sequence Archive in BIG Data Center, Beijing Institute of Genomics (BIG), Chinese Academy of Sciences, under accession number CRA000903 that is publicly accessible at <http://bigd.big.ac.cn/gsa>.

**Method S3 Statistics formulas for Fst, θ_w_, π and Tajima’s D calculations**

Fst was calculated using VCFtools with parameters ‘*--weir-fst-pop--fst-window-size --fst-window-step*’. The Fst is a measure of population differentiation estimated from the average pairwise differences between variant in each analysis panel compared to the combined samples as described in International HapMap Consortium (2005).

Tajima’s D (Tajima F, 1989) was calculated using VCFtools with parameter ‘*--TajimaD*’. Tajima’s D is computed as the difference between two measures of genetic diversity: the mean number of pairwise differences and the number of segregating sites, each scaled so that they are expected to be the same in a neutrally evolving population of constant size.


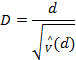


*D* is calculated by taking the difference between the two estimates of the population genetics parameter theta. This difference is called *d*, and *D* is calculated by dividing *d*, by the square root of its variance (its standard deviation, by definition).
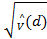


Nucleotide diversity (Nei's π, the average number of nucleotide differences per site between two DNA sequences chosen randomly from the sample population; Tajima F, 1983) was calculated using VCFtools with parameter ‘*--window-π*’.

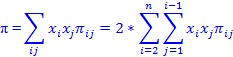


Where X_i_ and X_j_ are the respective frequencies of the i^th^ and j^th^ sequences, π_ij_ is the number of nucleotide differences per nucleotide site between the i^th^ and j^th^ sequences, and n is the number of sequences in the sample.

The Watterson estimate (θw), which is an estimation of population mutation rate (Watterson GA, 1975), was calculated on the basis of the number of segregating sites using the formula θw = S/a_n_ where S is the number of segregating sites and


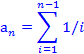


**Methods S4 Total RNA extraction, cDNA synthesis, and reverse transcription quantitative PCR**

Expression analysis was conducted to investigate the tissue or temporal expression patterns of the potentially associated genes in the population of *P. tomentosa*. The developing xylem tissues of upright stems, were collected by scraping the thin (approximately 1.0 mm) and the deep layer on the exposed xylem surface at breast height; the stem cambium were collected as described in Du *et al*., (2013). All tissues were immediately frozen in liquid nitrogen and stored at –80℃. We isolated total RNA from each fresh tissue pool (root, stem cambium, developing xylem, mature leaf, young leaf, and shoot apex) using an RNA extraction kit (TRIzol reagent; Invitrogen, Shanghai, China) according to the manufacturer’s instructions. Each pool was a mixture of the same tissue samples from ten random *P. tomentosa* individuals at a particular timepoint. Total RNA was extracted from various tissues using the Plant Qiagen RNAeasy kit (Qiagen China, Shanghai) according to the manufacturer’s instructions. Additional on-column DNase digestions were performed three times during the RNA purification using the RNase-Free DNase Set (Qiagen). RNA was then quantified and reverse transcribed into cDNA using the Super-Script First-Strand Synthesis system and the supplied polythymine primers (Invitrogen).

RT-qPCR was performed on a 7500 Fast Real-Time PCR System (ABI) using the Light Cycler-FastStart DNA master SYBR Green I kit (Roche). The PCR program included an initial denaturation at 94℃ for 5 min, and 40 cycles of 30 s at 94℃, 30 s at 58℃, and 30 s at 72℃, and a final melt-curve of 70–95℃. The specificity of the amplified fragments was checked by the melting curve. The expression values were analysed using Opticon Monitor Analysis Software v3.1 and standardized to the levels of *Actin1* using the 2^−ΔΔCt^ method. All reactions were performed with three technical replications and three biological replications.

**References:**

1. Du Q, Pan W, Tian J, Li B. Zhang D. (2013) The UDP-glucuronatedecarboxylase gene family in *Populus*: structure, expression, and association genetics, *PLoS One* **84**, e60880.
2. International HapMap Consortium (2005). A haplotype map of the human genome. *Nature* **437**: 1299–1320
3. Jia H, Osak M, Bogu G, Stanton L, Johnson R, Lipovich L (2010). Genome-wide computational identification and manual annotation of human long noncoding RNA genes. *RNA* **16:** 1478-1487.
4. Schmutz J, McClean P, Mamidi S, Wu G, Cannon S, Grimwood J, Jenkins J, Shu S, Song Q, Chavarro C (2014). A reference genome for common bean and genome-wide analysis of dual domestications. *Nature Genetics* **46**: 707-713
5. Tajima F (1983) Evolutionary relationship of DNA sequences in finite populations. *Genetics* **105**: 437–460
6. Tajima F (1989) Statistical method for testing the neutral mutation hypothesis by DNA polymorphism. *Genetics* **123**: 585–595
7. Watterson GA (1975) On the number of segregating sites in genetical models without recombination. *Theoretical Population Biology* **7:** 256-276.
8. Tafer H. and Hofacker, IL. (2008). RNAplex: a fast tool for RNA–RNA interaction search. *Bioinformatics* **24**, 2657–2663
